# A new role profile for nurses with expanded competencies promoting person-centered care in long-term care: a mixed-methods intervention development study

**DOI:** 10.1186/s12877-025-06086-2

**Published:** 2025-07-05

**Authors:** Katharina Silies, Tilman Huckle, Nadine Pohontsch, Anne-Marei Jarchow, Katrin Schütz, Martin Müller, Dagmar Lühmann, Katrin Balzer

**Affiliations:** 1 University of Lübeck, Institute for Social Medicine and Epidemiology, Nursing Research Unit, Ratzeburger Allee 160, 23562 Lübeck, Germany; 2 Department of General Practice and Primary Care, University Medical Center Hamburg-Eppendorf, Martinistr. 52, 20246 Hamburg, Germany; 3 Medical Faculty Heidelberg, Dept. Primary Care and Health Services Research, Heidelberg University, Nursing Science and Interprofessional Care, Im Neuenheimer Feld 130.3, 69115 Heidelberg, Germany

**Keywords:** Expanded nursing practice, Intervention development, Long-term care, Patient-centered care, Mixed-methods research, Patient involvement

## Abstract

**Background:**

The number of residents in nursing homes and the complexity of their care needs increase. A higher rate of nurses with higher qualification level is associated with a positive impact on patient outcomes such as quality of care, reduction of unplanned hospitalizations and emergency department use, and mortality. In Germany, defined role profiles for registered nurses with Bachelor’s degree in long-term care are lacking and only few of these nurses work in direct resident care.

**Objective:**

To develop a new role profile for nurses with expanded competencies to improve care for residents with complex care needs in long-term care.

**Methods:**

Design: Mixed-methods intervention development study following the PEPPA framework (Participatory, evidence-based, patient-focused process for advanced practice nursing role development, implementation, and evaluation).

We conducted sub-studies: 1. Three systematic literature reviews (on complex care needs, reasons for unplanned nursing home transfers to acute care, and context factors for decisions about transfers). 2. A multiple case study including five cases of nursing home residents to identify root-causes for unplanned transfers to acute care. Data collection: residents’ chart reviews and semi structured interviews with residents (*n* = 3), family (*n* = 4), and care providers (*n* = 11). Data analysis: root-cause analysis with event flow diagrams and qualitative content analysis of interviews to identify fields of action for the role profile. 3. Two participatory stakeholder workshops (*n* = 18 participants) to develop and refine intervention components and implementation strategies.

**Results:**

The new role profile comprises four competence areas: 1. Managing chronic diseases; 2. Empowerment and communication; 3. Person-centered care network; and 4. Organization. Main implementation strategy enabling nurses to fulfil the role profile is a 300-h additional qualification program. Further strategies on the organizational level are shared goal setting and allowing for adaptability of the intervention by defining mandatory and optional intervention components.

**Conclusions:**

The participatory intervention development approach resulted in a new role profile for nurses with Bachelor’s degree focusing on direct resident care. Feasibility, perceived usefulness and potential clinical effects of the intervention will be tested in a pilot trial with a cluster-randomized design and process evaluation.

**Trial registration:**

Prospectively registered on August 20th, 2021 at the German registry for clinical trials (DRKS00025773).

**Supplementary Information:**

The online version contains supplementary material available at 10.1186/s12877-025-06086-2.

## Background

In many countries worldwide, the number of older people needing professional care increases, including inpatient long-term care [1, 2]. In Germany, since 2013, the number of residents in long-term care facilities has increased by 3.8%, amounting to 793,000 (16%) of all care dependent persons in 2021 [3]. Additionally, the proportion of residents with the highest level of care dependency has increased by 5.9% in the past years [4–6]. Many residents in long-term care suffer from multiple chronic conditions such as pain, dementia, depression, diabetes mellitus or heart failure [7]. De facto, these conditions increase the risk of institutionalization into long-term care [8] and different morbidity patterns warrant individualized care plans [9]. Care needs of this target group can therefore be characterized as complex [10, 11]. These complex care needs can be addressed by person-centered care which is guided by individuals’ values and preferences and can improve patient experiences and outcomes as well as the quality of health care delivery [12, 13].

Furthermore, higher qualification levels of nurses are associated with a benefit for residents with complex needs, such as fewer unplanned nursing home transfers to acute care [14] and reduced mortality rate [15, 16]. Hospital transfers are burdensome and pose additional health risks for residents. Therefore, avoiding inappropriate hospital transfers of this target group is paramount [17, 18]. A higher proportion of registered nurses (RN) in long-term care has been associated with a lower rate of rehospitalization and emergency department visits [19]. Also, a higher skill mix in nursing teams, that is the ratio of RN staffing to total staffing, can be associated with lower incidence of pressure ulcers, urinary tract infections and lower rates of pain in nursing home residents [20]. Higher qualifications among nurses, such as academic degrees or specialized training, are therefore a relevant factor for the quality of long-term care.

In Germany, nursing education at university level is still relatively new. The majority of nurses have a vocational training of three years with a federally regulated examination. In addition, nurses can specialize in e.g. intensive care, geriatric care or psychiatric care. These specialization programs have an average duration of two years. Lower qualification levels are nurse assistants with 12 to 18 months education. With the reform of the Nursing Professions Act (PflBRefG [Pflegeberufereformgesetz]) [21], academic training for a Bachelor’s degree in general nursing has been established as an additional regular entry into the profession from 2020 on. A core task of nursing professionals with a Bachelor’s degree compared to those educated via vocational training is to strengthen evidence-based nursing and to handle the nursing process with more complex patients (PflBG [Pflegeberufegesetz] §37 (3))^22^. In 2021, the proportion of nurses with academic training was only 0.5% in long-term care in estimated full-time equivalents [3]. More than two thirds of those were employed in administrative positions rather than providing clinical nursing care, such as fundamental care and work in direct contact with residents [3]. The largest group of nursing staff providing direct care in long-term care are nurses with a three-year vocational training (43%). Thirteen percent are nursing assistants with a one-year vocational training. A substantial amount of 26% are staff without primary qualification related to nursing who have undergone a basic training to provide fundamental care [3]. The remainder are nurses in training (11%) and other health or social care professions. Advanced practice nurses with Master degree are exceptional in direct resident care.

Among the reasons for the lack of nurses with Bachelor’s degree involved in direct care is the uncertainty of roles and responsibilities for nurses with this qualification, especially in long-term care [23, 24]. Graduates feel a lack of opportunities to integrate their competencies into nursing practice and are more likely to pursue an academic career outside direct care [25]. An increasing number of graduates from 1 st cycle nursing education at university level is expected and they will provide an important human resource to meet current and future care needs in long-term care. Currently their training does not prepare them specifically for the rising complexity of elderly care in long term care. Therefore, to benefit from this resource in practice, a clear definition of the professional tasks that match their competence level and qualification concepts to support their entry in the setting are needed.

Internationally, three levels of nursing as a profession are widely established: registered nurse (RN), specialist nurse (SN) and advanced practice nurse (APN). Basic education is clearly regulated and there are definitions for advanced practice nursing [26]. Post-basic education, especially the “specialist nurse”, is less clear. In Europe, the education, certification, regulation and scope of practice of specialist nurses vary greatly. The importance to clarify competencies and the required education for specialization has therefore been emphasized [27, 28]. A policy model of nurses’ career pathway from RN to APN from Finland locates specialist nurses on Bachelor’s degree with additional education in the specialty and a role emphasis on clinical expertise, patient education and commitment to translate information into practice [29].

In the Expand-Care project, we strived to develop a role profile with expanded competencies, deepening those of nurses with a generalist Bachelor’s degree through a specialization in person-centered care for elderly residents with complex care needs. Expanded competencies can be defined as activities a) not usually carried out in this setting by a registered nurse, or b) that have formerly been undertaken by other professions, or c) that involve new technologies or procedures, or d) require a greater degree of autonomous judgement or intervention [30].

The Expand-Care project consisted of an intervention development phase and a pilot phase. Through the development phase, we aimed to 1. specify the need for expanded nursing competencies on bachelor level for evidence-based, person-centered long-term care of older people, 2. develop a role profile and tasks for nurses with expanded competencies, and 3. identify relevant contextual factors and develop strategies for a successful implementation of this role profile in long-term care.

## Methods

We used the Medical Research Council (MRC) guidance on the development and evaluation of complex interventions in health as a guiding meta framework [31]. We regarded the role and task profile of the Expand-Care nurses as a complex intervention, because it addresses more than one target group, comprises a bundle of intervention components and has to be delivered on different levels [31]. The MRC framework describes a research approach in four iterative steps: (1) Develop or identify intervention, (2) Feasibility, (3) Evaluation an (4) Implementation. Core considerations in each step should comprise context investigation, using program theory, engaging stakeholders, identifying key uncertainties, and continuous refining of the intervention.

The PEPPA framework describes a similar approach specifically for the development of an advanced role in nursing using participatory, evidence-based and patient-focused processes for guiding the development, implementation, and evaluation of new advanced practice nursing roles [32]. We employed this framework to shape the development process of the role profile. We adjusted the model slightly (“expanded nursing role” instead of “advanced nursing role”) to clarify that we do not target master qualification level in our study.

In particular, this intervention development study uses steps one to six of the PEPPA-framework with respective appropriate methods (see Table 1).

**Table 1:** PEPPA-Step and research methods in the Expand-Care study

Nr	PEPPA-Step	Methods
1	Define patient population and describe current model of care	Systematic literature searches and evidence syntheses
2	Identify stakeholders and recruit participants	Involving an external advisory board and institutions already collaborating with the nursing research unit
3	Determine need for a new model of care	Multiple case study Stakeholder workshop 1
4	Identify priority problems and goals to improve model of care	
5	Define new model of care and expanded nursing role	
6	Plan implementation strategies: Identify outcomes, outline evaluation plan, and role facilitators and barriers	Stakeholder workshop 2
*7*	*Initiate expanded nursing role implementation plan*	*Pilot study: cluster-randomized controlled trial*
*8*	*Evaluate expanded nursing role and new model of care*	*Pilot study: cluster-randomized controlled trial and process evaluation*

*PEPPA* Participatory, evidence-based, patient-focused process for advanced practice nursing role development, implementation, and evaluation [32]. Adapted: APN/Advance practice nursing replaced by Expanded Nursing for this specific study context

To plan implementation strategies for the new role (step 6 of the PEPPA framework), we used the German version of the Consolidated Framework for Implementation Research (CFIR) and Expert Recommendations for Implementing Change (ERIC) frameworks [33]. CFIR describes five domains of barriers relevant to implementation, while ERIC provides related structured strategies to overcome these barriers. Steps seven and eight of the PEPPA framework will be addressed by a pilot study testing the developed intervention (Study protocol: [34]).

The reporting of this manuscript follows guidelines for reporting intervention development studies in health research (GUIDED, [35]) and for reporting of patient and public involvement (GRIPP2-SF, [36]).

**Fig. 1:**
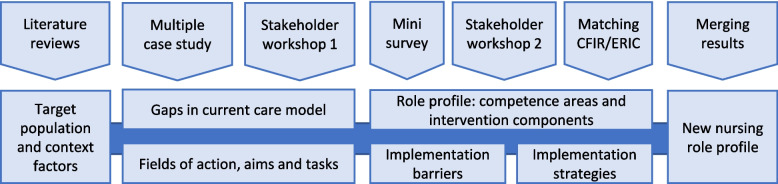
Overview of sub-studies, methods and results. CFIR: Consolidated framework for implementation research; ERIC: Expert recommendations for implementing change

To determine knowledge gaps and suitable research methods, we drafted a preliminary logic model, based on the process of the Evidence Based Practice Unit [37]. Our model contained the elements: target group, care model (intervention), outcomes of care, and contextual factors in long-term care. Based on this model we identified knowledge gaps regarding a) characteristics and needs of the target groups, b) the current care model, and especially c) gaps in care processes leading to unplanned acute care provision. We aimed to derive goals and outcomes of nursing care, and suitable tasks and competencies for a new nursing role profile. The iterative application of mixed methods required an integration of results at several points of the intervention development process (labelled as *interim work*). Sub-studies and their contribution to the final result are depicted in Fig. 1.

Involving key stakeholders in the process of role development represents an important part in the identification of needs and goals [32]. In this study, a multi-professional advisory board guided the research process through participation in workshops and board meetings. Participants were relevant stakeholders in long-term care with expertise in nursing practice and science, nursing management, health and social care insurance companies, nursing associations, social services and social affairs, medical law, general practitioners or as an advocate of residents.

### Literature reviews

#### Aim

To specify the target population, care routines (model of care, interventions) and context factors associated with acute escalation of care involving emergency medical services or hospital admission, as indicators of unmet complex care needs.

#### Population

Residents in long-term care facilities; health professionals in long-term care.

#### Outcomes

1. Characteristics and determinants of complex care needs; 2. Causes and events leading to use of emergency medical services and hospital admissions; 3. Contextual factors influencing decisions about hospitalization and emergency service use.

#### Study types (depending on outcome)

1. Systematic reviews, 2. Observational studies, 3. Systematic reviews.

See detailed overview of inclusion and exclusion criteria for all review questions in *supplement 1, table A2*.

#### Data collection

Systematic searches in MEDLINE via PubMed between May and October 2021. *See supplement 1, tables A3, A4 and A5 for detailed search strategies.*

#### Data extraction and analysis

We analyzed search results depending on the outcome: 1. categorization of results and summaries according to “definition of complex care needs”, “reasons and conditions leading to complex care needs”, and “recommendations or interventions to address complex needs”; 2. occurrence of conditions and reasons for transfers and utilization of emergency medical services by type of contact and type of reason; and 3. involved parties or agents, organizational, and system level of contextual factors influencing transfers and utilization of emergency medical services. *See supplement 1, table A7 for overview of extracted data items and chapter A, paragraph f for synthesis methods.*

### Multiple case study

#### Aims

1. To analyze care processes of residents in long-term care preceding hospitalizations and 2. to identify which structures, processes and outcomes lead to breaks or gaps in the continuity of care.

#### Participants

As cases we defined residents in long-term care who had experienced an unplanned hospital transfer or contact with emergency medical services in the previous eight weeks (key event). Through this inclusion criterion we aimed to detect processes indicating unmet complex care needs of residents. We further considered relatives or proxies of residents, registered nurses and nursing assistants, nursing managers, general practitioners and therapists if they had been involved in care processes associated with the case. We aimed to interview these stakeholders about the observed care processes.

#### Recruitment and sampling

In August 2021 we invited long-term care facilities already collaborating with the University to take part in the case study. Sampling of cases aimed at a heterogeneous sample in terms of residents’ cognitive status and type of key event. Residents meeting inclusion criteria, and proxies, if applicable, received verbal and written information about the study. We invited further stakeholders (health care professionals) to participate in interviews in writing and per telephone. Written informed consent of participants or their legal surrogates was mandatory for participation. The ethics committee of the University of Lübeck approved of the study (Nr. 21–301).

#### Data collection

We extracted data from residents’ records with standardized forms and conducted semi-structured audiotaped interviews with the people involved in care processes of the respective resident. All data belonging to a case were linked through an ID number. Data collection ended in October 2021.

#### Data analysis

We followed the steps for root cause analysis [38] and mapped an event flow diagram with communication processes, and interventions for each case. We identified causal factors for gaps in care and derived themes associated with the key event on the individual cases’ level. For this, we analyzed the content of related interviews according to themes emerging from the event flow diagram. Subsequently, we synthesized meta themes across cases and derived preliminary fields of action, goals for care and potential nursing tasks.

#### Study registration

German registry for clinical trials (DRKS00025773). A full report of the multiple case study will be published elsewhere [39].

### Stakeholder workshop 1

#### Aim

To identify potential/prototypical care problems to be addressed by the new role profile, fields of action with defined nursing tasks, and required competencies of Expand-care nurses.

#### Recruitment

We recruited experts and stakeholders from different fields of expertise relevant to the long-term care setting (e.g. nurses, physicians, resident representatives, *see supplement 1, table B15 and B17, participant overview*). We addressed persons who had already submitted a written cooperation agreement during the application process of the project and the advisory board. Further, nursing homes collaborating in the University’s Bachelor program in nursing supported the recruitment of academic nursing staff.

#### Workshop methods

The workshop took place via a video conference platform (Webex by Cisco [40]). Members of the research team moderated the workshop. We presented a case scenario based on results of the literature searches and the multiple case study. Based on this case scenario, three tasks were assigned to the participants: 1. To discuss barriers or gaps in current nursing care from their own experience, 2. To formulate necessary changes to address these gaps including potential tasks for a new nursing role profile, and 3. To prioritize and assess the feasibility of the previously discussed tasks. All questions were discussed in small groups first and then presented and discussed in plenary sessions.

#### Data collection and analysis

We documented the results using an online whiteboard (Miro [41]) during the workshop. Additionally, two researchers independently recorded discussions and results in written minutes. The research team synthesized data from the minutes and the miro board thematically after the workshop. The resulting list of nursing tasks was incorporated in the next development steps. (*See supplement 1, chapter B for detailed methods*).

### Interim work

We synthesized results from literature reviews, multiple case study and the first workshop. Through repeated discussions in our interprofessional research team (nurses, physician, psychologist), we developed and clustered a range of action fields with defined goals and nursing tasks and grouped these inductively into four competence areas. Additionally, we compared our results to existing competency frameworks on nursing specialist roles in long-term care [42]. The comparison on the level of nursing tasks led to minor adjustments of our model. The resulting role profile provided the basis for the development of implementation strategies with stakeholders in a second workshop.

### Mini-survey

To prepare the discussion in workshop 2 we launched a mini-survey with participants of the first workshop and all other members of our advisory board.

#### Aim

To rate the previously synthesized potential nursing tasks and competence areas regarding their priority for a new nursing role profile in long-term care facilities.

#### Survey recruitment and data collection

We conducted an online survey between December 2021 and January 2022 using the open source survey tool Limesurvey [43]. We sent out invitations to workshop 1 participants and advisory board members by e-mail and collected data anonymously (*see supplement 1, table B15 invited panelists*). We presented four competence areas comprising 24 fields of action and 55 nursing tasks to be rated individually by participants. An English translation of the survey is provided in *supplement 1, chapter B*, *additional material*.

#### Analysis of survey data

For each nursing task, priority could be rated on a scale of 1 to 9 in which numbers from 1 and 3 indicated a “low priority”, numbers from 4 and 6 a “general importance, but not an essential priority” and numbers from 7 to 9 the “highest priority and crucial importance” [44]. We calculated frequency, median and interquartile range using the SPSS software for statistical analysis [45] for each task rated in the survey.

### Stakeholder workshop 2

#### Aim

To define the final set of intervention components of the nursing role and identify implementation barriers and strategies.

#### Recruitment

Recruitment strategy was the same as for the first workshop.

#### Workshop methods

The workshop took place via a video conference platform (Webex by Cisco [40]). Members of the research team moderated the workshop. It consisted of three phases. In phase 1 we presented and discussed survey results. In phase 2 participants identified and rated implementation barriers. For this, we used the domains and barriers listed in the consolidated framework for advancing implementation research (CFIR) [33, 46]. With the survey tool integrated in WebEx, participants anonymously voted for barriers with the highest relevance to the implementation of a new nursing role in long-term care per domain. Each participant was allowed to award half as many points as there were barriers in a domain. In phase 3 we discussed potential strategies in the implementation of new nursing roles in long-term care. We focused on the barriers that participants had identified as most relevant in phase 2.

#### Data collection and analysis

Two researchers independently recorded discussions and results in written minutes. We calculated frequencies of the rating results and discussed and summarized data from the minutes thematically.

### Interim work

We built on the workshop results to derive implementation strategies and used participants’ rating of implementation barriers for the CFIR-ERIC matching tool (ERIC: Expert Recommendations for Implementing Change, [47]). We compared ERIC recommendations rated with more than 100% through the matching tool with suggestions participants had discussed in the second workshop. We combined these into strategies addressing the individual level (e.g. education) and the organizational level (communication and cooperation).

### Final intervention and implementation strategies

Merging results across all steps in the process, we revised our preliminary logic model with regard to the target groups, change mechanisms and context factors of the intervention and the implementation. We described intervention components of the new nursing role and implementation strategies according to the template for intervention description and replication (TIDieR, [48]).

## Results

Here, we briefly describe results of the different sub-studies and how they contributed to the intervention development (section A). More detailed information on all methods and results of the sub-studies are provided in *supplement 1*. The overall result, i.e. the intervention, comprises the newly developed role profile for nursing professionals with expanded competencies, and strategies to implement this role profile (reported in section B).

### Section A: Results of sub-studies

#### Literature reviews

##### Review 1: Characteristics and determinants of complex care needs.

We included 10 studies in this review: evidence syntheses including studies of mixed designs (*n* = 7), clinical trials (*n* = 1) and qualitative studies (*n* = 2), *see supplement 1, table A8 for details on study characteristics*. We categorized complex care needs with regard to specific groups of residents, specific care situations, and general situations perceived as complex. Specific groups with complex care needs are for example residents with dementia, obesity or palliative care needs. Specific care situations resulting in complex care needs are for example nutrition, hydration, infectious diseases and chronic wounds. General situations perceived as complex are enabling residents’ autonomy and individual care needs, or the organization, planning and management of resources. *See supplement 1, table A9 for the data synthesis of complex care situations.*

##### Review 2: Causes and events leading to use of emergency medical services and hospital admissions.

We included 35 studies all of which were observational studies reporting occurrence of conditions or causes for hospital transfers or contacts with emergency medical services, *see supplement 1, table A10 for study characteristics*. Most studies reported causes for transfer to emergency department or hospital (32/35). Conditions were summarized in different categories: orthopedic (falls, injuries), cardiovascular, neurologic, gastrointestinal, and respiratory symptoms; general deterioration of health (infections, fever, dehydration, pain); transfers due to behavior or mental status changes; and care related causes such as pressure ulcers or complications with urinary catheters. Leading conditions across all included studies were falls and traumatic injuries followed by infections and fever. *See supplement 1, tables A11 and A12 for data synthesis on prevalence and admission rate of different causes for acute medical service provision.*

##### Review 3: Contextual factors influencing decisions about hospitalization and emergency service use.

We included eight studies, all of which were systematic literature reviews including studies of different designs, *see supplement 1, table A13 for study characteristics*. Contextual factors of decisions for or against acute care measures were located on different levels: the individual level of health care professionals, the organizational level and the health system in general. Competencies regarding recognizing and managing acute changes in health, lack of confidence, fear of reprimands or litigation, inadequate care planning, or communication barriers with staff, physicians and acute care institutions are exemplary nurse-level factors.

*In summary,* results of all three literature reviews indicated 6 potential fields of action: 1. nursing assessments regarding chronic conditions, exacerbation of symptoms, management of infections and behavioral symptoms including interpreting and communicating results; 2. interprofessional communication and collaboration including a better understanding of health professionals’ roles and communication pathways and processes; 3. advance care planning and ethical reasoning; 4. comprehensive care planning and decision-making; 5. empowerment and participation of residents; 6. educational interventions for residents, family members and nursing staff. *See supplement 1, table A14 for synthesis of factors influencing the use of emergency medical services*. The summary of each of the three literature reviews’ contributions to a new nursing role profile is displayed in *supplement 1, Figure A5*.

#### Multiple case study

Within the *multiple case study*, we analyzed five cases of residents with hospitalizations due to falls, heart failure, and general health deterioration. We interviewed residents (*n* = 3), family (*n* = 4), and care providers (*n* = 11). In total, we identified seven meta themes as overarching areas relevant to expanded nursing care: 1. geriatric nursing care, 2. interprofessional collaboration, 3. empowerment of residents, 4. health promotion and prevention, 5. communication processes, 6. management, 7. values, standards and guidelines.

Each meta theme comprised fields of action to address specific goals of care and potential nursing tasks to address these goals.

As an example, the first meta theme, *Geriatric nursing care* refers to the management of chronic and geriatric diseases, especially regarding geriatric nursing assessments, detection of health deterioration, re-evaluation of care plans and support of medical therapy. Eight fields of action are summarized in this first meta theme: 1.1 Symptom control in chronic diseases; 1.2 Nursing support for medical care; 1.3 Understanding health as a life course process; 1.4 Case-management; 1.5 Evaluation of the care situation; 1.6 Reaction to nursing assessments; 1.7 Quality of care based on current scientific standards; 1.8 Nursing support in the therapy of chronic diseases.

Field of action *1.1 Symptom control in chronic diseases*, aims at the early detection of exacerbation of chronic diseases. Potential expanded nursing tasks to achieve this goal are the implementation of in-house protocols for assessment tools, carrying out these assessments and ensuring reaction in a timely manner.

In summary, the meta themes found in the multiple case study highlighted the need for improved continuous and proactive care management for residents with chronic illnesses and acute health deterioration. Furthermore, needs for improved communication within the nursing profession and the facility as well as with other health professionals and institutions became visible. Thus, results of the multiple case study supplemented and enriched results of the literature reviews. A full description of the multiple case study’s methods and results will be published elsewhere [39].

#### Stakeholder workshop 1

Ten stakeholders representing different perspectives participated in the 3.5 h workshop in November 2021 (*n* = 2 nursing managers, *n* = 2 nurses with Bachelor’s degree, *n* = 2 general practitioners, *n* = 2 representatives of residents and senior citizens board, *n* = 1 nurse scientist, *n* = 1 nursing association representative). In three steps, relevant fields of actions for nurses with expanded competencies were identified and discussed. First, participants hypothetically derived current or future care problems based on a scenario of a resident with a chronic condition. Problems were seen for example with regard to involvement of residents and family in care, documentation of medical care, or adequate allocation of tasks among staff. Second, participants discussed nursing tasks to address these problems and to prevent a worsening of the resident’s care situation. Third, these tasks were rated with regard to their relevance (usefulness) and feasibility. Relevant and feasible tasks were for example establishing communication protocols following international standards for handovers [49] and communication with general practitioners. Relevant but difficult to implement tasks were for example telemedicine (video consultation) or shared digitalized documentation of medical and nursing care. The latter were not pursued in the further development due to feasibility considerations.

In summary, highest relevance and feasibility were seen in: prospective care planning in case of current health deterioration or change of needs, implementation of new communication processes, improvement of nursing interventions in fall prevention and respiratory diseases, education of nursing staff, and management of advance directives of residents.

#### Interim work

We used results from all sub-studies conducted until this point to synthesize 24 fields of action and aims with 55 nursing tasks. Through repeated team discussions, we subsequently grouped and assigned them to four main competence areas: 1. managing chronic and geriatric diseases; 2. empowerment and communication with residents; 3. building and maintaining a person-centered care network; and 4. organizational level (*Supplement 1, chapter B, tables B19 to B23*).

#### Mini-survey

Through a *mini-survey*, 13 participants anonymously rated nursing tasks in the newly derived four main competence areas regarding their priority for the new role profile. Tasks rated with highest priority were for example: implementation and interpretation of a wider range of geriatric assessments (competence area 1, managing chronic and geriatric diseases); a short checklist and overview of residents’ core health information for hospital stays, or case conferences with residents, relatives, and health professionals (competence area 3, person-centered care network). Further highly important tasks were to offer structured advice on advance care planning to residents and family (competence area 2, empowerment and communication) or to establish training and learning opportunities on evidence-based care, expert standards and nursing guidelines in nursing homes (competence area 4, organizational level). Full results of the mini-survey are provided in *supplement 1, Chapter B, table B20 to B23*.

#### Stakeholder workshop 2

The workshop took place in January 2022 via WebEx by Cisco and lasted four hours. Eight stakeholders participated (*n* = 1 nursing home managers; *n* = 1 general practitioner; *n* = 2 nursing scientists; *n* = 1 medical lawyer; *n* = 3 representatives, one each of residents, senior citizens, and a nursing association). In the first step, participants discussed the four competence areas and associated fields of action based on the rating of the mini-survey. Importantly, participants emphasized that the new role profile should focus on direct resident care and management of chronic and geriatric diseases. In the second phase, we presented implementation barriers according to the five domains of the CFIR framework [33]. Participants rated as most important barriers: unclear relative advantage and lacking adaptability (domain innovation characteristics), lacking peer pressure and supportive external policy and incentives (domain outer setting); lack of leadership engagement and resources (domain inner setting); individual stage of change (domain characteristics of individuals), and lack of support from opinion leaders and key stakeholders (domain process). To maintain feasibility, participants emphasized that the role profile should consist of a manageable range of nursing tasks (*full results: supplement 1, Chapter B, table B29, CFIR constructs rated as important*).

#### Finalization of intervention components and implementation strategies

Following the second workshop, we synthesized nursing tasks further into core intervention components (mandatory) and optional components, with an overall focus on direct resident care. In this process of repeated discussions in the multiprofessional research team, we especially considered direct, resident related nursing tasks for intervention components as participants in the stakeholder workshops advised to prioritize these tasks.

### Section B: Intervention, intervention components and implementation strategies

#### Intervention

**Fig. 2:**
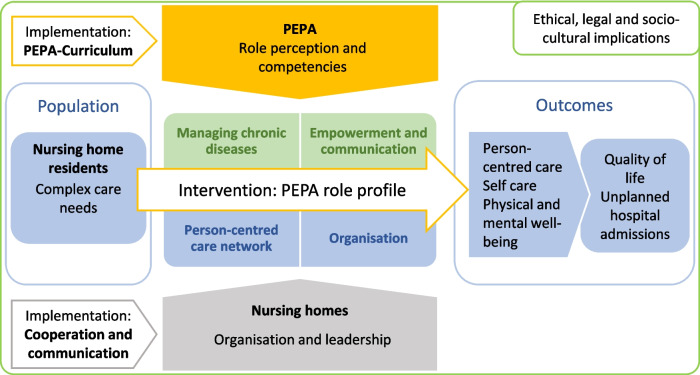
Logic model of the Expand-Care intervention. PEPA: nurse specialist [Pflegefachperson mit erweiterten Kompetenzen für personenzentrierte Pflege in der Altenpflege], adapted from: [34]

The final result of the intervention development process is a new nursing role profile to improve person-centered care in long-term care. The role profile consists of a bundle of nursing tasks (intervention components) related to four competence areas. The logic model of the role profile displays target groups, the intervention components, outcomes on a proximal and a distal level and the context on micro, meso and macro level (Fig. 2, Logic model of the Expand-Care intervention). We named the role profile “PEPA”, a German acronym for nurse specialists with expanded competencies for person-centered care in long-term care.

The aim of the PEPA role profile is to improve intermediate outcomes such as person-centered care, self-care of residents with chronic diseases, and physical and mental symptom burden. Ultimately, patient-relevant outcomes such as health-related quality of life, unplanned emergency care and hospital admissions should be addressed. Through an improved targeted care planning, the PEPA aims to support a timely observation of changes in residents’ condition. Necessary interventions/services may thus be better anticipated and initiated or adapted in a timely manner.

#### Intervention components

The PEPA role comprises core (mandatory) and optional tasks related to four competence areas: 1. managing chronic diseases; 2. empowerment and communication (with residents and relatives); 3. building and maintaining a person-centered care network; 4. (developing the) organization. Intervention components are further characterized as more resident-related tasks, such as regular structured conversations with residents and relatives about goals of care, as opposed to more organization-related tasks, such as implementing structured communication within and between professions (Table 2).

**Table 2:** Overview of intervention components

Intervention components of the nursing role profile	
*Core components*	*Optional components*
*Resident-related tasks in direct care*	
**• Structured process of care planning and evaluation** defined by events related to the individual situation of the residents based on a structured care plan (*algorithm displayed in Supplement 1, chapter C, figure C7*) **•** Regular structured conversations with residents and relatives about goals of care **•** Organization and coordination of joint visits with general practitioners **•** Initiation and organization of interdisciplinary case conferences **•** Hospital visits for hospitalized residents to obtain information on changed needs **•** Pain assessment and management **•** Regular geriatric and nursing assessments	**•** Implementation of a short information sheet describing resident´s needs for health professionals outside of the long-term care facility
*Tasks on the organizational level*	
**•** Nursing staff handover according to ISBAR communication structure **•** Implementation of ISBAR-structured (fax) communication with general practitioners **•** Providing training for nursing staff on ISBAR and emergency situations **•** Monitoring of residents’ advance care planning status	**•** Participation in evidence-based practice development in cooperation with research facility **•** Supervision and consultation for colleagues

ISBAR: structure for interprofessional communication, consisting of introduction, situation, background, assessment, recommendation

The core resident-related task ‘structured process of care planning and evaluation’ is the central intervention component for deciding on the use and linkage of further tasks in the care process, for example nursing assessments or structured conversations with residents or relatives (*Supplement 1, Chapter C, figures C6 and C7 shows the algorithm guiding this process*). Specific events, such as health deterioration or hospital admission and discharge, are defined as initiators for the structured process. First steps are gathering of information (structured conversation with resident and/or relatives, complex nursing assessment according to established tools (SIS® [50]) and specific assessments as needed. Based on these, further necessary intervention components can be chosen. For example, after a hospital stay, major changes in resident’s health status may result in the need for a case conference to agree on a revised care plan. *Supplement 1, figure C7* shows a graphic display of this process.

#### Implementation strategies

We devised several implementation strategies targeting individual education (PEPA-Curriculum), and models for communication and cooperation with nursing homes. We described all intervention components and implementation strategies according to the TIDieR template [48]. The full description is published elsewhere [34]

Key implementation strategy is a curriculum for a 300-h training for registered nurses led by lecturers of the University of Lübeck which aims to prepare participating nurses to apply the intervention components. The training encompasses 10 credit points based on the European Credit Transfer System and aligns with established continuous education programs for specialization in nursing in Germany. It consists of two modules, the contents being 1. role development, legal and ethical principles of expanded competencies, evidence-based practice, communication, coaching and 2. basic medical knowledge of chronic diseases, geriatric assessment, prevention, self-care, case management, patient-centered care, shared-decision making and advance care planning. The program is split into 100 h each of classroom/online teaching, self-regulated study time, and training on the job in the long-term care facility. The training program aims to enhance PEPAs’ knowledge of person-centered care. They shall be supported in developing understanding of the new role and in acquiring competencies to transfer the role into daily care. A handbook for documenting participation in courses and other learning activities, as well as for documenting learning objectives (reviewed through practice supervision), shall increase the commitment and show PEPAs their learning progress. Detailed learning objectives are documented in the PEPA curriculum (*supplement 2*).

On the nursing home level, strategies target communication and cooperation*.* Target setting meetings with the nursing manager should take place at the beginning and the end of the training program. The aim of these meetings is to communicate about a shared idea of good care and to set goals as to how the intervention (role of PEPA) can support this. Thereby the nursing manager shall be involved in the project and thus the implementation of the intervention supported. Hindering and supporting factors should be discussed and solutions sought if necessary.

To support implementation on the organizational level, we defined an adaptability of the intervention. The PEPA intervention comprises a bundle of components (Table 2), some of which can be implemented optionally, whereas core tasks are mandatory. The possibility to adapt the intervention to the individual circumstances and needs of the institution shall support identification with the intervention and subsequently implementation. Furthermore, a formal declaration of commitment through a cooperation agreement shall increase the binding nature of the respective responsibilities of the partners (care facility and universities) in the project. Thereby, compliance with the project plan shall be supported, in particular the release of PEPAs off regular duties, and the implementation of the curriculum.

## Discussion

In this study, we developed the role profile of nurses with expanded competencies for person-centered care for residents with complex care needs in long-term care facilities following the first six steps of the PEPPA framework [32]. Informed by results of literature reviews, a multiple case study and workshops, we identified four competence areas for nurse specialists: 1. management of chronic diseases, 2. empowerment of and communication with residents, 3. building and maintaining a person-centered care network and 4. (developing the) organization. The role profile addresses interprofessional communication through the implementation of structured communication instruments. As main implementation strategy we developed a curriculum for a 300-h training program for higher qualified nurses taking place at the university and at the workplace. To improve commitment to the implementation of the newly developed role profile, facilities will sign cooperation agreements and participate in target setting meetings. The intervention is adaptable through the integration of optional tasks, allowing participants to place a focus on their institution’s specific needs. Finally, we summarized the role profile comprising intervention components, implementation strategies, mechanisms of change and contextual factors in a logic model.

The role profile developed in this study is located at a continuum of increasing competency levels between the registered nurse as a starting point and the advanced practice nurse as highest qualification level [30].

Internationally, role profiles for graduate nurses in direct care are implemented to a varying degree. In countries with longer tradition of academic education in nursing, advanced role profiles such as nurse practitioner and clinical nurse specialist are more established and role development and specialization focuses on these roles [51]. A review of reviews portraying a global perspective of advanced practice nursing research showed that the majority of studies included originated from the United States and the United Kingdom. [52]. In countries with younger history of academic education in nursing, Master nurses are less present in direct care, especially in long-term care settings. Bachelor level nursing education is mostly generalist, specific skills that are related to the respective target group and care setting are not addressed in-depth and roles are blurred [53]. With the increasing complexity of care in long-term care, and a lack of APN with master qualification that provide clinical expertise onsite, the need for specialization and expanded competencies of bachelor qualified nurses becomes evident. This applies to the German long-term care setting, and the intervention we developed addresses this gap.

Research has shown that roles for expanded practice in long-term care are not only needed to improve quality of care, but also to retain qualified workforce in this setting. Working in long-term care seems to be rated second best in comparison to working in hospital by many nurses. Reasons are seen for example in limited opportunities to expand clinical skills, to strengthen professional identity, and to access interesting career pathways [54]. A successful way to expand these competencies can be continuous professional development combining learning opportunities on the job as well as external to the work environment [55]. In this study, we developed a comprehensive training program with blended learning elements onsite and outside of the facility. Our approach is therefore in line with current evidence and can contribute to the development of clinical leadership competencies and the retention of qualified workforce in this field.

New roles for nurses with expanded competencies are often located at hospitals where nurses work closely with physicians. Research suggests that these role profiles can enfold even greater impact in primary care or environments where a physician is not constantly available [56]. Such is the situation in German long-term care facilities that hardly have any onsite physicians. Experiences in the US and Canada showed the benefit of nurse practitioners in long-term care facilities [57]. Importantly, we want to point out that advanced practice nursing (APN) roles such as nurse practitioners require a Master’s degree according to international conventions which is not the level addressed by our intervention [26]. Still, we emphasized clinical skills such as management of chronic diseases. To support nurses in specializing and implementing their new role, we developed the comprehensive additional 300-h training program and thus aim to improve care for residents with these conditions.

### Strengths and limitations

During the intervention development, we encountered several challenges. It is difficult for health care professionals to anticipate a role profile that currently does not exist and is to be executed by a new generation of nursing professionals whose wider scope of competencies is yet mostly unknown to the majority of stakeholders. Also, to promote improvements through these new roles, it is necessary to concede to pitfalls of today’s care provision without belittling the current workforce and quality of care. Therefore, to engage all stakeholder groups in research may be challenging. Through the universities’ good local partnerships with care providers, we successfully managed to recruit at least one representative of each group in the multiple case study, advisory board meetings and stakeholder workshops. Still, a higher representation of general practitioners and nurses working at the bedside would have been desirable.

In our study, we conducted several sub studies which have some limitations. In the literature reviews, we used one relevant data base (Medline), and hand searches, which may have limited the scope of the evidence included. Also, we refrained from critically appraising the included studies. For qualitative data we applied an integrative approach [58]—more interpretative methods might have yielded more in-depth results, for example regarding concepts such as complex care. We did not apply comprehensive validated consensus methods to interim work phases that led to the synthesis of sub studies’ results, but based it instead on repeated consensus processes within our multiprofessional research team. Still, our overall research process comprised sub studies of varied methodology to address open research questions specifically. The results of these sub studies complemented and supported each other. We report overall results as well as interim steps and through this provide a transparent description of our research process.

A decisive strength in our study is the adherence to established guidance for the development of advanced nursing practice [32]. Although we did not aim to develop an ANP profile which would require a qualification on master level, the application of this guidance supported the development of roles that exceed the traditional scope of practice of registered nurses. Furthermore, we were thus able to address core elements of the state-of-the-art framework for developing and evaluating complex interventions, such as *consider context, develop and refine program theory, engage stakeholders, identify key uncertainties, and refine the intervention* [31].

The successful implementation and effects of an intervention may depend highly on context. We addressed *context* in our literature work as well as in stakeholder workshops, considering the CFIR framework [33] as basis for context-related barriers to implementation and developed implementation strategies accordingly.

To *develop and refine our program theory*, we started with a preliminary logic model of the current care model and identified knowledge gaps which we subsequently filled through the different methods we applied during the intervention development. As a result, we developed a refined logic model of the intervention expanded with detailed descriptions of our assumptions on change mechanisms in a TIDieR template (Why, how and for whom? [48]). While the logic model itself is a simplified graphic illustration of the intervention, together with the TIDieR template it provides a sound basis for a comprehensive process evaluation in a pilot trial. Thus, it will enable us to illuminate assumed mechanisms of action and interdependencies, and detect remaining *uncertainties* of the program theory and to further *refine the intervention*.

Throughout our research process, we repeatedly *engaged stakeholders*, as advisors and as participators in research through interactive stakeholder workshops. Thus, for example, we were able to refine the intervention’s main focus to resident-related tasks, whereas organizational or inter-organizational tasks were given less weight.

Experiences from different European countries further showed that role development and implementation is an evolutionary process which can take 15 to 20 years. Especially in early stages, a close liaison between practitioners, managers and educators is essential for a successful progress [59]. Through the engagement of the stakeholders, and our own role as University which provides nursing education, our study may contribute to the evolvement and professional recognition of expanded nursing roles in the future.

## Conclusion

The Expand-Care intervention is a meticulously developed new role profile for nurses with expanded competencies for person-centered care for residents with complex care needs in long-term care. It meets the need to establish specific clinical roles for nurses with Bachelor’s degree in this setting to improve quality of nursing home care. The training program can support nurses’ development of specialized skills. As a next step, feasibility and potential benefits of the intervention will be tested in a pilot trial. The results will provide evidence for the further development of expanded nursing roles and their potential effects on health, especially of vulnerable groups like older people in need of long-term care. Thus, the study presented here may serve as a first step towards implementing specific clinical roles for bachelor educated nurses in German long-term care.

## Supplementary Information

Supplementary Material 1.

Supplementary Material 2.

## Data Availability

Multiple case study and stakeholder workshop minutes: The datasets generated and analyzed during the current study are not publicly available (anonymization is not feasible due to the nature of the data (in-depth qualitative data), thus prohibiting data sharing on open access repositories), but are available from the corresponding author on reasonable request.

## Abbreviations

APN: Advance practice nursing, advanced practice nurse
CFIR: Consolidated framework for implementation research
ERIC: Expert recommendations for implementing change.
GRIPP2-SF: Guideline for reporting of patient and public involvement
GUIDED: Guidelines for reporting intervention development studies in health research
ID number: Identification number
MRC: Medical Research Council
PEPA: Nurse specialist [German acronym, Pflegefachperson mit erweiterten Kompetenzen für personenzentrierte Pflege in der Altenpflege].
PEPPA framework: Participatory, evidence-based, patient-focused process for advanced practice nursing role development, implementation, and evaluation
RN: Registered nurse
SN: Specialist nurse
TIDieR: Template for intervention description and replication
