# Supplementary material for: A new role profile for nurses with expanded competencies promoting person-centered care in long-term care: a mixed-methods intervention development study
Source: BMC Geriatr. 2025 Jul 5;25:492. doi: 10.1186/s12877-025-06086-2 (PMC12228253; doi:10.1186/s12877-025-06086-2)
Supplement: Supplementary file 1 — Supplementary Material 1. [file 12877_2025_6086_MOESM1_ESM.pdf]

Supplement 1 to

A new role profile for nurses with expanded competencies promoting person-centered care in long-term care: a mixed methods intervention development study.

Silies K, Huckle T, Pohontsch N, Jarchow AM, Schütz K, Müller M, Lühmann D, Balzer K

Table of contents

Directory of tables and figures 2

Chapter A: Literature reviews 4

1. Methods 4
   a. Information sources and time frame 4
   b. Eligibility criteria 4
   c. Search strategies 6
   d. Selection and data extraction process 8
   e. Data items and effect measures 9
   f. Synthesis methods 10
   g. Bias 11
   h. Reporting of results 11
2. Results RQ1 - complex care situations 12
   a. PRISMA Flowchart RQ1 12
   b. Study characteristics RQ1 13
   c. Synthesis RQ1 19
3. Results RQ2 - causes for initiation of emergency medical care 20
   a. PRISMA Flowchart RQ2 20
   b. Study characteristics RQ2 21
   c. Synthesis RQ2 44
4. Results RQ3 –factors influencing the use of emergency medical care 46
   a. PRISMA Flowchart RQ3 46
   b. Study characteristics RQ3 47
   c. Synthesis RQ3 50
5. Summary of results regarding a new nursing role in long-term care 51
6. Literature 54
   a. Complex care situations 54
   b. Causes for emergency services 55

c. Factors influencing the use of emergency medical services 57
Chapter B: Stakeholder workshops and survey 59
1. Aims 59
2. Methods 59
a. Eligibility criteria and recruitment 59
b. Data collection 60
c. Description of workshop 1 60
d. Results of workshop 1 60
e. Mini-survey 62
f. Results of survey data 63
g. Description of workshop 2 68
h. Results of workshop day 2 69
3. Literature 71
Additional material to chapter B: Translated mini-survey questionnaire 72
Chapter C: Algorithm for decisions on intervention components 85

Directory of tables and figures

Table A1: Literature reviews: research questions (RQ) 4
Table A2: Inclusion and exclusion criteria according to each research question 5
Table A3: Search strategy RQ1: Complex care situations 6
Table A4: Search strategy RQ2: Causes for the initiation of emergency medical care 7
Table A5: Search strategy RQ3: Factors influencing the initiation of emergency medical care 8
Table A6: Researchers involved in selection and data extraction process 8
Table A7: Extracted data items according to research question 9
Table A8: Study characteristics complex care situations 13
Table A9: Data synthesis complex care situations 19
Table A10: Study characteristics causes for emergency medical services 21
Table A11: Data synthesis of causes for the initiation of medical services (prevalence) 44
Table A12: Data synthesis of main causes for the initiation of medical services (admission rate) 45
Table A13: Study characteristics factors influencing use of emergency medical services 47
Table A14: Data synthesis factors influencing use of emergency medical services 50
Table B15: Background and number of panellists invited to take part in the workshops 59
Table B16: Content of workshop 1 60
Table B17: Participants in workshop 1 60
Table B18: Tasks or competencies discussed in workshop 1 61
Table B19: Areas of competencies and fields of action 62
Table B20: Results for “Development and maintenance of a person-centered care network” (n=13) 63
Table B21: Results of “Management of chronic and geriatric diseases” (n=12) 64

Table B22: Results of “Empowerment and communication with residents” (n=12) 65
Table B23: Results “Organisation and nursing home facility” (n=11) 66
Table B24: Number of tasks rated with the highest importance per area of competency 67
Table B25: Themes emerging from panellists’ comments 67
Table B26: Contents of workshop 2 68
Table B27: Domains and constructs of CFIR framework (Damschroeder et al., 2009) 68
Table B28: Participants at workshop 2 69
Table B29: CFIR constructs rated as important by participants 69

Figure A1: PRISMA Flowchart complex care situations... 12
Figure A2: PRISMA flow chart causes for emergency medical services 20
Figure A3: PRISMA flowchart factors influencing use of emergency medical services........................ 46
Figure A4: Domains of complex needs 51
Figure A5: Synthesis of all three literature reviews regarding a new nursing role in long-term care .. 53
Figure C6 Key events for care planning and evaluation 85
Figure C7 Planning and evaluation algorithm 86

Chapter A: Literature reviews

Table A1: Literature reviews: research questions (RQ)

**RQ1**	To identify characteristics and determinants of complex care situations and needs for residents of long-term care facilities
**RQ2**	To identify causes and events that lead to the initiation and contact of emergency medical care and hospital admissions in residents of long-term care facilities
**RQ3**	To identify contextual factors which influence decisions on the use of emergency medical care for residents of long-term care facilities

1. Methods

The following is a description of methods and results of the three literature reviews in accordance with the PRISMA statement for reporting systematic reviews and meta-analyses of studies that evaluate healthcare interventions (Page et al., 2021).

a. Information sources and time frame

All three literature searches took place during the time period of May 2021 to October 2021 (table A2) in the database MEDLINE via PubMed. Additionally, for objective 3 a hand search via google scholar was conducted in May 2021.

b. Eligibility criteria

Criteria and other characteristics of the three literature searches are displayed in table A2. Only published studies were eligible with limitation to the last 10 years. Searches (1) and (3) included systematic reviews whereas for search (2) only observational studies were eligible. All three searches excluded respite care, the population and setting being residents in long-term care in nursing homes. Outcomes in (1) and (3) were of a qualitative nature, the aim being descriptions and aspects of complex care situations respectively contextual factors associated with contacts with emergency medical care. Outcomes in (2) were frequencies of causes and events that lead to the initiation of emergency medical care. All studies addressing the outcomes to any degree were eligible to be included in the review.

Table A2: Inclusion and exclusion criteria according to each research question

	Characteristics and determinants of complex care needs	Causes and events leading to use of emergency medical services and hospital admissions	Contextual factors influencing decisions about hospitalization and emergency service use
**Population**	Residents in long-term care		
**Setting**	Long-term care/nursing home		
**Outcomes**	Descriptions of causes and aspects of care based on assumed criteria of the complex care situation:<br>– a particularly high resource requirement or burden for carers<br>– unmet needs of the person in need of care<br>– the need for intensive multi-professional care<br>– the presence of multi-morbidity or a combination and interaction of various health and care aspects	Outcomes for naming and identifying the causes, diseases, events or conditions and their frequencies that lead to the initiation of medical services such as contacts with the rescue service, the on-call service, hospital admission, unplanned general practitioner contacts	Description of contextual factors, determinants, conditions, structures, processes that are associated with unplanned contacts with emergency services, departments, out-of-hour primary care, or hospitalisation of nursing home residents
**Types of studies included**	Systematic reviews	Observational studies	Systematic reviews
**Language**	English and German	English and German	English and German
**Years considered**	31.10.2011 to 31.10.2021	21.05.2011 to 21.05.2021	25.05.2011 to 25.05.2021
**Exclusion criteria**	– Respite care<br>– Umbrella Review<br>– Review of reviews	– Respite care<br>– Studies, that solely report on economic outcomes<br>– Studies, in which only one outcome is analysed<br>– Events that occur during the use of emergency medical care	– Respite care

c. Search strategies

The PubMed search strategies for each objective are listed below (tables 3, 4 & 5).

Table A3: Search strategy RQ1: Complex care situations

PICO-D	#	Keywords	Hits
**P**	1	long term care [tw]	39.974
	2	nursing home [tw]	23.329
	3	residential facilities [tw]	6.238
	4	residential facilities [MeSH]	55.592
	5	Long term care [MeSH]	27.148
	6	Nursing home [MeSH]	41.918
	**7**	**1 OR 2 OR 3 OR 4 OR 5 OR 6**	**93.801**
**O**	8	complex care need* [tw]	480
	9	complex need* [tw]	1.886
	10	complexity of care [tw]	467
	11	complexity of nursing [tw]	99
	12	patient complexity [tw]	398
	13	complex [tw]	1.479.409
	14	Nurses Improving Care for Health System Elders [MeSH]	1
	15	health services needs and demand [MeSH]	61.933
	16	complex needs [MeSH]	2.769
	**17**	**8 OR 9 OR 10 OR 11 OR 12 OR 13 OR 14 OR 15 OR 16**	**1.539.893**
**Design**	19	meta-analysis [Publication Type] OR meta analysis [Title/Abstract] OR meta analysis [MeSH Terms] OR review [Publication Type] OR search* [Title/Abstract]	3.328.660
	**20**	**7 AND 17 AND 19**	**680**
	21	Filter: Abstract	631
**Language**	22	Filter: English, German	613
**Time**	23	Filter: Last 10 years (31.10.2011 to 31.10.2021)	284
**Results**			**284**

Table A4: Search strategy RQ2: Causes for the initiation of emergency medical care

PICO	#	Keywords	Hits
**P**	1	long term care [tw]	39.974
	2	nursing home [tw]	23.329
	3	residential facilities [tw]	6.238
	4	residential facilities [MeSH]	55.592
	5	Long term care [MeSH]	27.148
	6	Nursing home [MeSH]	41.918
	**7**	**1 OR 2 OR 3 OR 4 OR 5 OR 6**	**92.232**
**O1**	8	incident* [tw]	157.246
	9	cause* [tw]	2.448.412
	10	indication* [tw]	297.369
	11	event* [tw]	1.053.892
	12	condition* [tw]	2.298.082
	13	reason* [tw]	465.690
	14	diagnosis [MeSH Terms]	8.933.916
	15	signs and symptoms [MeSH Terms]	2.213.266
	16	symptom* [tw]	1.250.782
	17	diagnos*[tw]	5.367.685
	**18**	**8 OR 9 OR 10 OR 11 OR 12 OR 13 OR 14 OR 15 OR 16 OR 17**	**15.551.794**
**O2**	19	referral [tw]	157.128
	20	transition [tw]	365.747
	21	admission [tw]	218.097
	22	transfer [tw]	545.265
	23	patient transfer [MeSH Terms]	9.196
	24	patient transfer [tw]	9.888
	**25**	**19 OR 20 OR 21 OR 22 OR 23 OR 24**	**1.235.342**
**O3**	26	hospital [MeSH Terms]	294.755
	27	emergencies [MeSH Terms]	42.060
	28	emergency medical service [MeSH Terms]	155.299
	29	emergency medical service [tw]	4.657
	30	emergency medicine [MeSH Terms]	14.705
	31	emergency medicine [tw]	26.979
	32	emergenc* [tw]	481.987
	33	Unplanned[tw] AND (general practitioners [MeSH] OR general practitioners [tw])	110
	**34**	**26 OR 27 OR 28 OR 29 OR 30 OR 31 OR 32 OR 33**	**779.172**
	**35**	**7 AND 18 AND 25 AND 34**	**891**
	36	Filter: Abstract	839
**Language**	37	Filter: English and German	805
**Time**	38	Filter: Last 10 years (21.05.2011 to 21.05.2021)	429
**Results**			**429**

Table A5: Search strategy RQ3: Factors influencing the initiation of emergency medical care

PICO-D	#	Keywords	Hits
**P**	1	long term care [tw]	39.974
	2	nursing home [tw]	23.329
	3	residential facilities [tw]	6.238
	4	residential facilities [MeSH]	55.592
	5	Long term care [MeSH]	27.148
	6	Nursing home [MeSH]	41.918
	**7**	**1 OR 2 OR 3 OR 4 OR 5 OR 6**	**92.232**
**O1**	8	contextual factor* [tw]	7.960
	9	Context [tw]	456.471
	10	Process [tw]	1.360.257
	11	Structur* [tw]	2.693.999
	12	Condition [tw]	569.103
	13	Determinants [tw]	172.103
	**14**	**8 OR 9 OR 11 OR 12 OR 13**	**4.801.915**
**O2**	15	Unplanned[tw] AND (general practitioners [MeSH] OR general practitioners [tw])	110
	16	Hospital [tw]	1.334.076
	17	emergenc* [tw]	482.373
	18	emergency medicine [tw]	26.998
	19	emergency [MeSH]	42.069
	20	Emergency medical service [tw]	4.659
	21	Hospital [MeSH]	294.897
	22	Emergency medical service [MeSH]	155.387
	23	Emergency medicine [MeSH]	14.708
	**24**	**15 OR 16 OR 17 OR 18 OR 19 OR 20 OR 21 OR 22 OR 23**	**1.787.188**
	**25**	**7 AND 14 AND 24**	**2.504**
**Design**	26	Systematic [sb]	73
**Language**	27	Filter: English and German	73
**Time**	28	Filter: Last 10 years (25.05.2011 to 25.05.2021)	62
**Results**			**62**

d. Selection and data extraction process

For each literature review, two researchers (overall: KS, TH, AMJ, KSc) independently reviewed titles and abstracts. Studies were assessed for eligibility using the criteria displayed in table 2. Before starting the screening, all four researchers met and discussed relevant aspects of the eligibility criteria as well as the screening method. Disagreements in title and abstract screening were resolved between reviewers by consensus and if necessary, a third researcher was consulted. Subsequently each full-text screening was done by two independent reviewers, with the same procedure in case of disagreement. The process and results of screening were displayed in flow charts for each objective accordingly.

All researchers extracted data of included studies independently (table 6). The extracted data was checked by at least one other reviewer and discussed. Disagreements between researchers were resolved by discussion in order to reach consensus.

Table A6: Researchers involved in selection and data extraction process

	RQ1	RQ2	RQ3
Title and abstract screening	TH, KS, KSc	TH, KS, AMJ	KS
Full-text screening	TH, KS, KSc	TH, KS, AMJ	KS, KSc
Data extraction process	KSc, TH	TH, AMJ	KS, TH

RQ: Research question

Data extraction and study characteristics templates were developed according to each objective and eligibility criteria. The templates were developed by two researchers with any discrepancies being resolved through discussions (TH, KS).

e. Data items and effect measures

Data items are displayed in table A7. Outcomes for the objectives (RQ1) and (RQ3) were of a qualitative nature. Any data eligible to be included in the data synthesis was extracted. Outcomes for objective (RQ2) were eligible, if causes or reasons for emergency medical care were displayed with absolute and relative frequencies within a variety of different frequencies of causes or reasons for hospitalization.

Table A7: Extracted data items according to research question

Data item	RQ1	RQ2	RQ3
Study characteristics			
Authors	X	X	X
Year	X	X	X
Design	X	X	X
Aim	X	X	X
Setting		X	
Sample size		X	
Target groups			X
Search resources and time of search	X		X
Methods of data synthesis / statistical analysis	X	X	X
Study types and number of studies included in review	X		X
Outcomes	X	X	X
Data extraction			
Description of complex care situations:			
• Complex care needs	X		
• Condition and causes for complexity	X		
• Recommendations and/or interventions	X		
Description and prevalence of causes leading to emergency medical care:			
• Description of type of emergency medical care		X	
• Description of analysed condition, event, reason, indication, cause, symptom or incident		X	
• Prevalence of conditions, events, reasons, indication, causes, symptoms or incidents		X	
Description of contextual factors, determinants, conditions, structures, processes that are associated with contacts or influence the use of emergency medical services			X

RQ: Research question

f. Synthesis methods

RQ1 Description of complex care situations:

Data was extracted according to (1) the description of the complex care need, (2) the condition and underlying causes for the complexity and if reported or discussed (3) recommendations or interventions addressing the complex care need. Subsequently, data was summarized within the three categories and reviewed within the research group (TH, KSc).

RQ2 Description and prevalence of causes leading to emergency medical care:

Data extraction from included studies was the description of the type of emergency medical care reported (hospital admission, admission to emergency department only, contact with on-call medical services, contact with emergency medical service), the description of the condition or reason for the emergency medical care and frequency of analysed condition or reason. The frequency of the condition or the reason within a study population was extracted if it was reported in absolute and relative frequencies or admission rates per residents. Both types of frequencies were synthesised separately.

Extracted reason or conditions were summarized within nine main categories following the method of thematic content analysis:

- Orthopaedic reason/condition
- Cardiovascular reason/condition
- Neurologic reason/condition
- Respiratory reason/condition
- Deterioration of health including fever, dehydration and infections
- Care related reasons
- Gastrointestinal reason/condition
- Behaviour change
- Other conditions

Reasons or conditions were included in the synthesis, if they accounted for at least 10 % of admissions or contacts within the study population. Frequencies were not synthesized mathematically. Extraction and synthesis process were reviewed within the research group (TH, AMJ).

RQ3 Description of factors influencing the use of emergency medical care:

Descriptions of any contextual factors, determinants, conditions, structures, processes that are associated with decisions for unplanned contacts with emergency medical services were extracted. Data was subsequently synthesised by the research team (KS, TH) into six different categories regarding factors within different groups of stakeholders or institutions in long-term care:

- Physician factors
- Nurse factors
- Resident factors
- Family factors
- Organisational / nursing home factors
- Health system factors

g. Bias

Since the purpose of this literature review was to gather information to support and inform the next steps in the development of the intervention, no risk of bias assessment was conducted or extracted.

h. Reporting of results

Results for all three research questions are reported by a display of the PRISMA flowchart, a description of the characteristics of included studies and a synthesis of extracted data.

2. Results RQ1 - complex care situations

a. PRISMA Flowchart RQ1

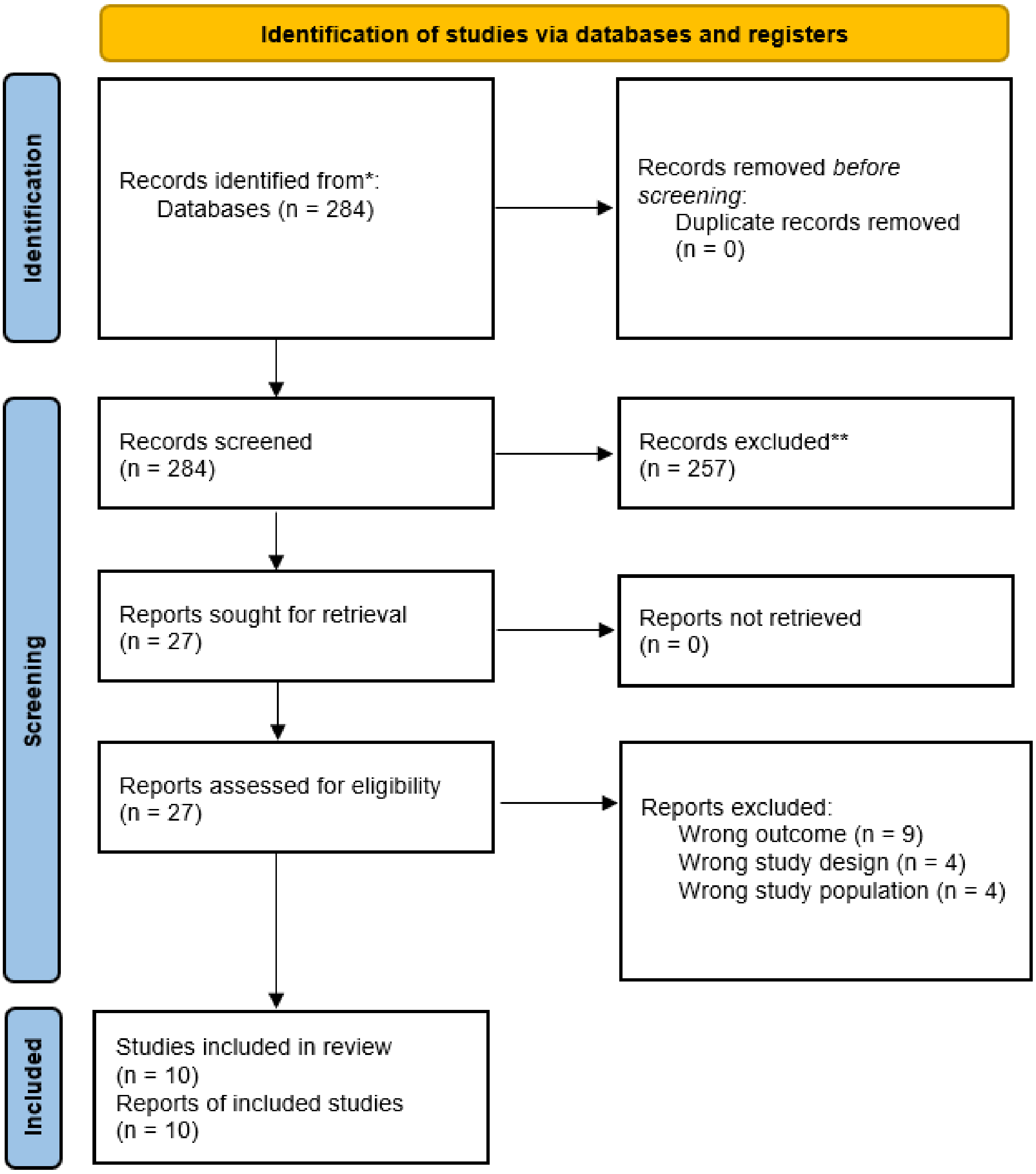

Figure A1: PRISMA Flowchart complex care situations

b. Study characteristics RQ1

Table A8: Study characteristics complex care situations

Authors (year)	Study design	Aim	Search resources and time of search	Methods of data synthesis	Study types and countries: number of studies	Findings
Bolt, S. R., van der Steen, J. T., Schols, J. M. G. A., Zwakhalen, S. M. G., Pieters, S., & Meijers, J. M. M. (2019)	Scoping review	To establish an integrated conceptualization of needs regarding<br>• needs in providing palliative dementia care<br>• dementia care as perceived by nursing staff in home care or in long-term care facilities	Cinahl, PubMed, PsycINFO;<br>search finished December, 22nd, 2017	Qualitative studies: data charting form adapted from Joanna Briggs Institute (JBI) recommendations (Peters et al., 2015, Methodology for JBI Scoping Reviews)<br>Quantitative study: according to findings of the authors of the article	qualitative study n= 13<br>quantitative study n= 1<br>Mixed methods n= 1<br>• Europe n= 8<br>• USA n= 4<br>• Australia n= 1<br>• Asia n=2	Nursing staff needs on a direct care-level recognizing and addressing palliative needs, verbal and nonverbal communication, challenging behaviour and familiarity.

Authors (year)	Study design	Aim	Search resources and time of search	Methods of data synthesis	Study types and countries: number of studies	Findings
Cadieux, M. A., Garcia, L. J., & Patrick, J. (2013)	Systematic review	To determine care needs of people with dementia living in long-term care by using the best evidence possible	Medline, Cinahl, Health-Star, Embase, PsycINFO, Cochrane library;<br>January 2000 to September 2010	Identification of a list of needs; categorization all needs into 19 categories	Quantitative design n= 50 (of which 14 randomized controlled trials)<br>Qualitative design n= 15<br>Mixed design n= 3<br>• USA n = 37<br>• United Kingdom n= 8<br>• Canada n = 7<br>• other countries n= 16	Care needs most discussed by people with dementia.
Cook, G., Hodgson, P., Thompson, J., Bainbridge, L., Johnson, A., & Storey, P. (2019)	Review	To provide an overview of the literature related to hydration interventions to support care home residents to drink sufficient fluid;<br>to highlight gaps in knowledge and important areas for future research	Applied Social Sciences Index and Abstracts (ASSIA), CINAHL, Medline, ProQuest Hospital Premium Collection, Cochrane Library and RCN databases;<br>2013 to 2019	Data extraction was completed using PICO	• cross over study, United Kingdom n=1<br>• trial, United Kingdom n=1<br>• historically controlled study, Japan n=1<br>Complementary:<br>• day project, Germany n= 1<br>• RCT, United Kingdom n= 1<br>• protocol of a multi-centre cross-sectional study, Canada n=1	Hydration levels as an outcome measure alongside other factors or descriptive measures to assess effectiveness of interventions, such as changes to overall hydration consumption in the care facility.

Authors (year)	Study design	Aim	Search resources and time of search	Methods of data synthesis	Study types and countries: number of studies	Findings
Crosbie, B., Ferguson, M., Wong, G., Walker, D. M., Vanhegan, S., & Dening, T. (2019)	Realist synthesis	To explore how effective care is provided for residents in long-term care settings and to approach specific areas of hearing-related communication needs of care home residents living with dementia	Medline, Embase, PsycINFO, Cinahl, ISI Web of Science, British Nursing Index (BNI) Cochrane Library, U.S. National Library of Medicine Clinical trials.gov<br>(1980 to March 2018)	Theory-driven approach including using expert opinion to understand complex health situations and developing context-mechanism-outome (CMOC) configurations	Various different study designs including grey literature<br>• USA n= 13<br>• United Kingdom n= 12<br>• Canada n= 10<br>• Australia n= 5<br>• New Zealand n= 1<br>• Norway n= 1	Context-mechanism-outcome configurations (CMOC).
Fasullo, K., McIntosh, E., Buchholz, S. W., Ruppar, T., & Ailey, S. (2022)	Integrative review	To synthesize literature about lesbian, gay, bisexual, transgender, and queer (LGBTQ) older adults in long-term care facilities and to recommendations to inform best practices in caring for LGBTQ older adults within long-term care settings	PubMed, Cinahl, Scopus, LGBT Life;<br>2000 to 2019	Whittemore & Knafl's framework for data analysis as an integrative review methodology: data reduction, data display, data comparison, conclusion drawing, verification.	Quantitative design n= 8<br>Qualitative design n= 9<br>Mixed-methods n= 3<br>• USA n=12<br>• Europe n= 6<br>• Oceania n= 2	Two categories of themes:<br>• perspective of LGBTQ participants<br>• perspective of long-term care staff

Authors (year)	Study design	Aim	Search resources and time of search	Methods of data synthesis	Study types and countries: number of studies	Findings
Fleming, A., Bradley, C., Cullinan, S., & Byrne, S. (2015)	Meta-Synthesis of qualitative research	To synthesize the findings of qualitative studies investigating the factors influencing antibiotic prescribing in long-term care facilities	Embase, PubMed, PsycInfo, Social Science Citations Index, Google Scholar;<br><br>Up to July 2014	Thematic synthesis following the ENTREQ (enhancing Transparency in Reporting the Synthesis of Qualitative Research) guidelines.	Qualitative design n= 8<br>• Canada n= 4<br>• Netherland & USA n= 1<br>• Australia n= 1<br>• England n= 1<br>• Northern Ireland n= 1	Identification of prominent or recurring themes in the literature and collation under thematic headings.
Harris, J. A., & Castle, N. G. (2019)	Systematic Review	To describe the scope and depth of evidence regarding the impact of obesity among nursing home residents in the United States	PubMed, Embase, Cinahl, Web of Science;<br><br>1997 to march 2017	Data synthesis with standardized tables in accordance with the systematic search principles from the Preferred Reporting Items for Systematic Reviews and Meta-Analyses (PRISMA), Guidelines	• cohort study n= 14<br>• cross-sectional n= 9<br>• case study n= 4<br>• simulation n= 1<br><br>• USA: n= 28	• comorbid medical and functional conditions<br>• costs<br>• health system effects<br>• mortality<br>• nursing practices<br>• risk of admission to long-term care
Kiljunen, O., Välimäki, T., Kankkunen, P., & Partanen, P. (2017)	Integrative review	To identify the competence needed for older people (with complex needs) nursing in licensed practical nurses' and registered nurses' work in care and nursing homes	Cinahl, Ovid Medline, PsycInfo, SocIndex, Scopus;<br><br>2006 to April 2016	Analysis using qualitative content analysis (Whittemore and Knafl's method)	• quantitative design n= 2<br>• qualitative design n= 8<br><br>• Sweden n= 4<br>• Norway n= 2<br>• United Kingdom n= 1<br>• Switzerland n= 1<br>• USA n= 1<br>• Netherlands n= 1	Five competence areas for registered nurses:<br>• attitudinal<br>• ethical<br>• interactional<br>• evidence-based care<br>• pedagogical<br>• leadership<br>• development competence

Authors (year)	Study design	Aim	Search resources and time of search	Methods of data synthesis	Study types and countries: number of studies	Findings
Means T. (2016)	Systematic review	To obtain evidence-based information that will assist in identifying beneficial training to raise quality standards and reduce avoidable hospital admissions as part of the new care home support team project for frail older adults residing in residential care.	British Nursing Index, Medline and Cumulative Index of Nursing and Allied Health Literature (CINAHL) databases;<br><br>2001 to 2016	Not reported	• independent studies n= 9<br>• literature review n = 5<br><br>• United Kingdom n= 14	• education, teaching and training<br>• competencies and skills<br>• care home, residential home, nursing home<br>• tissue viability, dementia awareness, palliative care, falls prevention, continence management, long-term conditions,<br>• nutrition and hydration and diabetes management<br>• care staff, nurses, nursing assistants, health care assistants<br>• residents care, quality care
Watkins, R., Goodwin, V. A., Abbott, R. A., Backhouse, A., Moore, D., & Tarrant, M. (2017)	Systematic review of qualitative literature	To better understand factors that may contribute to malnutrition by examining the attitudes, perceptions and experiences of mealtimes among care home residents and staff	Medline, Embase, PsycInfo, AMED, Cochrane; search for grey literature: Health Management Information Consortium (HMIC), Social policy and practice (SPP);	Thematic analysis	All studies had qualitative components in order to be included:<br>• comparison studies n= 5<br>• cross sectional studies n= 10<br><br>• Denmark n= 3<br>• United Kingdom n= 3<br>• Australia n= 2	• organizational and staff support<br>• resident agency<br>• mealtime culture<br>• meal quality and enjoyment

Authors (year)	Study design	Aim	Search resources and time of search	Methods of data synthesis	Study types and countries: number of studies	Findings
			From inception to November 2015		• Canada n= 2<br>• USA n= 1<br>• Sweden n= 1<br>• Guyana n= 1<br>• Spain n= 1<br>• Netherlands n= 1	

NH: Nursing home; ED: Emergency department, LGBTQ: Lesbian gay bisexual transsexual and queer

c. Synthesis RQ1

Table A9: Data synthesis complex care situations

Complex care needs	Underlying causes and conditions	Recommendations regarding nursing skills and competencies
**Specific care situations:**<br>• nutrition and hydration management<br>• ethical dilemmas<br>• palliative and end-of-life care<br>• infection and antibiotic management<br>• management of long-term conditions<br>• management of chronic wounds<br>• management of falls and continence problems<br>• supervising and educating residents, their family members, student nurses and nursing staff<br>**Specific groups of long-term care residents with complex needs:**<br>• residents with dementia<br>• residents receiving palliative care<br>• residents with obesity<br>• members of the LGBTQ community<br>• residents suffering from hearing loss<br>**General situations that are perceived as complex:**<br>• enabling autonomy, supporting individuality and integrity<br>• organising, planning and managing resources<br>• planning, assessment, health promotion, risk management	**Causes related to illness and health:**<br>• often a number of different, inter-related medical conditions prevalent<br>• multimorbid, vulnerable, seriously ill and dying older people<br>• antimicrobial resistance<br>• severe cognitive deficits, such as memory loss, apraxia and aphasia, comorbid disease, infections and symptoms such as dyspnoea, pain and agitation<br>• limited ability to communicate and difficulties in understanding<br>• ageing, dependency and high levels of frailty<br>**Causes regarding stakeholders involved in care:**<br>• social factors influencing prescribing of antibiotics<br>• distrust between doctors and nurses<br>• ethical challenges due to involvement of family<br>**Causes regarding nurses' skills and resources:**<br>• lack of awareness of the early signs and symptoms<br>• lack of awareness of the challenges faced by LGBTQ older adults<br>• lack of resources in general<br>• inadequate physical space in the nursing home	**Skills, competencies and knowledge regarding:**<br>• pain recognition and management<br>• the use of assessment tools and choice of medication<br>• hydration management<br>• competence in communication impaired by hearing-related reasons<br>• sensitivity for needs regarding members of the LGBTQ community<br>• multidisciplinary collaborative strategies<br>• attitudinal, ethical, interactional, evidence-based care, pedagogical and leadership and development competence<br>**Furthermore**:<br>• clear identification of competencies required in practical nurses' work in care and nursing homes<br>• need for staff to have clear specific goals

3. Results RQ2 - causes for initiation of emergency medical care

a. PRISMA Flowchart RQ2

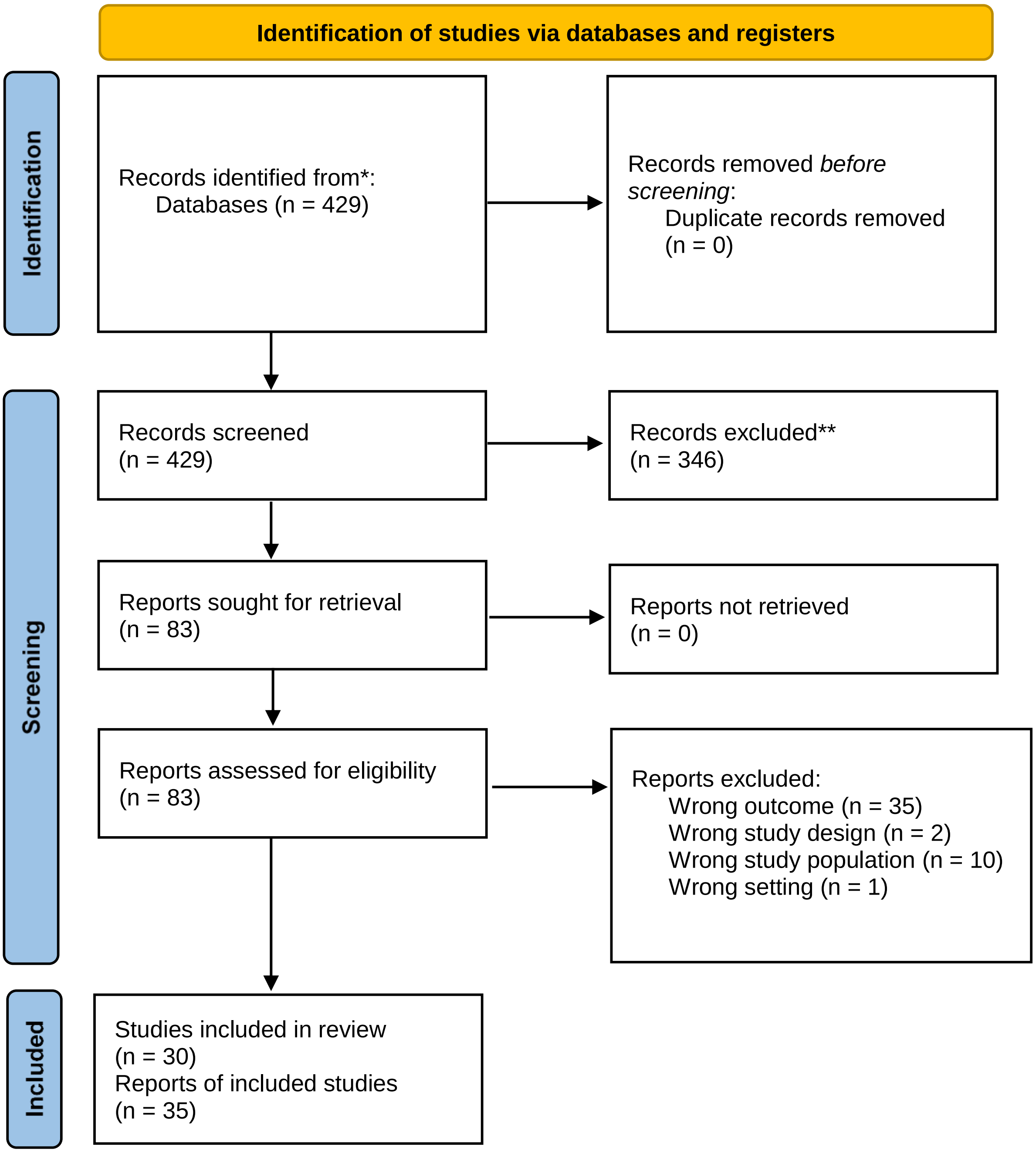

Figure A2: PRISMA flow chart causes for emergency medical services

b. Study characteristics RQ2

Table A10: Study characteristics causes for emergency medical services

Authors (year)	Study design	Aim of the study	Setting	Sample size	Statistical analysis	Prevalence of conditions, events, reasons, indication, causes, symptoms or incidents with sample size of study population, n
Guion, V., De Souto Barreto, P., & Rolland, Y. (2020)	Observational multicenter study	To describe the trajectories of pain, dyspnoea, fever, confusion, agitation and fatigue of nursing home residents' (NHRs) after a transfer to the emergency department	Emergency departments	751 nursing home residents	Group-based multi-trajectory modelling	**Nursing home residents n = 751**<br>Orthopedics (without fracture) 214 (28.5 %)<br>Breathlessness 135 (18.0 %)<br>Complex polypathology 104 (13.9 %)<br>Sepsis 103 (13.7 %)<br>Orthopedics (with fracture) 75 (10.0 %)<br>Cardiology 40 (5.3 %)<br>Other 37 (4.9 %)<br>Stroke 29 (3.9 %)<br>Biology 14 (1.9 %)
Guion, V., De Souto Barreto, P., & Rolland, Y. (2021)	Case-control observational multicenter study	To describe nursing home residents' (NHRs) functional trajectories and mortality after a transfer to the emergency department (ED).	Emergency departments	751 nursing home residents	Cox regressions	**Nursing home residents n = 1,037**<br>Orthopedics (without fracture) 260 (25.1 %)<br>Breathlessness 214 (20.6 %)<br>Complex polypathology 161 (15.5 %)<br>GI or GU sepsis 143 (13.8 %)<br>Orthopedics (with fracture) 82 (7.9 %)<br>Cardiology 54 (5.2 %)<br>Other 53 (5.1 %)<br>Stroke 50 (4.8 %)<br>Biology 20 (1.9 %)

Authors (year)	Study design	Aim of the study	Setting	Sample size	Statistical analysis	Prevalence of conditions, events, reasons, indication, causes, symptoms or incidents with sample size of study population, n
Briggs, R., Coughlan, T., Collins, R., O'neill, D., Kennelly, S.P. (2013)	Observational study	To prospectively profile and characterize all NH residents presenting to an urban hospital ED in order to clarify some of the current and future challenges of providing emergency care to this group	Emergency Department of a university teaching hospital	155 visits by 116 NH residents	Frequencies and percentages; Mean and standard deviation; calculation of proportionate comparisons between different groups using chi square test; relative risk; differences in the length of stay were measured using t-test	**ED visits n = 155**<br>Pneumonia 45 (29.0 %)<br>Falls 26 (16.8 %)<br>UTI 13 (8.4 %)
Brownstein, H., Hayes, B., Simadri, A., Tacey, M. and Holbeach, E. (2021)	Observational study	To explore the characteristics of Aged Care Facility residents transferred to hospital in the last 24hours of life and factors that may influence this decision	Metropolitan Emergency Department	149 patients who died within 24 hours of arrival	Descriptive statistical analysis; chi-squared tests and Fisher exact tests to test for differences; continuous variables assessed for normality using parametric and/or non-parametric tests	**Residents n = 149**<br>Shortness of breath 68 (45.6 %)<br>Altered conscious state 47 (31.5 %)<br>Poor oral intake 5 (3.4 %)<br>Abdominal pain 10 (6.7 %)<br>Fall 7 (4.7 %)<br>Vomiting 5 (3.4 %)<br>Other 7 (4.7 %)

Authors (year)	Study design	Aim of the study	Setting	Sample size	Statistical analysis	Prevalence of conditions, events, reasons, indication, causes, symptoms or incidents with sample size of study population, n
Kirsebom, M., Hedstrom, M., Wadensten, B., Poder, U. (2013)	Retrospective, descriptive study	To examine the frequency of and reason for transfer from nursing homes to the emergency department (ED), whether these transfers led to admission to a hospital ward	Municipality hospital	594 transfers among a total of 431 residents	Descriptive statistics and non-parametric analyses	**Transfers to the ED n = 594**<br>Falls and/or injuries incl. fractures 147 (24.7 %)<br>Respiratory symptoms 67 (11.3 %)<br>GI Pain 60 (10.1 %)<br>CNS symptom 55 (9.3 %)<br>General deterioration: weakness 51 (8.6 %)<br>Infection UVI, fever, sepsis, pneumonia 50 (8.4 %)<br>Pain, not specified 46 (7.7 %)<br>Cardiovascular symptoms 37 (6.2 %)<br>Urinary problems (not UVI related) 32 (5.4 %)<br>Miscellaneous 26 (4.4 %)<br>Chest pain 21 (3.5 %)<br>Cardiac arrest 2 (0.3%)
Amador, S., Goodman, C., King, D. et al. (2014)	Observational study	To examine the frequency, reasons, outcomes and factors associated with emergency ambulance service use in people with dementia resident in residential care homes	Residential care homes	133 residents across the six care homes	Logistic regression analyses	**Nursing home residents n = 144**<br>Trauma 60 (41.7 %)<br>Respiratory 13 (9.0 %)<br>Cardiovascular complaint 10 (6.9 %)<br>GI complaint 8 (5.6 %)<br>Genitourinary complaint 8 (5.6 %)<br>Altered mental status 7 (4.9 %)<br>Non-specific complaint 6 (4.2 %)<br>Cerebrovascular complaint 5 (3.5 %)<br>Muscoskeletal, no trauma 4 (2.8 %)<br>Seizure 3 (2.1 %)<br>Circulatory complaint 1 (0.7 %)<br>Ear, nose & throat problem 1 (0.7 %)<br>Unknown 18 (12.5 %)

Authors (year)	Study design	Aim of the study	Setting	Sample size	Statistical analysis	Prevalence of conditions, events, reasons, indication, causes, symptoms or incidents with sample size of study population, n
Carron, P. N., Mabire, C., Yersin, B., & Büla, C. (2017)	Retrospective monocentric study	To investigate the evolution over time of the number of ED visits by NH residents in an academic medical centre, and to describe these ED visits as well as their outcomes	Emergency department at University Hospital	3.590 ED visits by NH residents over six years	Descriptive statistics; differences in continuous variables were assessed using Spearman test and Kruskal–Wallis test; differences in proportions were analysed using Chi-squared test	**[n not reported in original paper]**<br>Injury 32.0 %<br>Respiratory problems 12.9 %<br>Cardiovascular problems 11.9 %<br>Digestive problems 9.6 %<br>Neurological problems 8.8 %<br>Others 24.8 %
Fassmer, A. M., Pulst, A., Schmiemann, G., & Hoffmann, F. (2020)	Part of the HOspitalisations and eMERgency department visits of Nursing home residents (HOMERN) project, a multicenter prospective study	To investigate sex-specific differences in characteristics of transferred residents and reasons for hospital transfers	Nursing homes	626 hospital transfers in 14 NHs	Descriptive statistics; logistic regression analyses	**Unplanned Transfers n = 535**<br>Deterioration of health status (e.g., fever, infection) 188 (35.1 %)<br>Fall, accident, injury 179 (33.5 %)<br>Psychiatric/neurologic conditions 38 (7.1 %)<br>Complications with catheter/tube (blood in urine) 38 (7.1 %)<br>Pain, not fall-induced 33 (6.2 %)<br>Others 59 (11.0 %)
Walker, R. W., Palmer, J., Stancliffe, J., Wood, B. H., Hand, A., & Gray, W. K. (2014)	Retrospective study	To investigate the factors that precipitate entry to institutional care, and on-going care needs once in care, within a representative cohort	Nursing home	90 people with PD living in care homes	The Wilcoxon signed rank test was used to compare participants before and during care home stay; Mann–Whitney U-test (emergency	**Residents n = 90**<br>Patient or spouse inability to cope 47 (52.2 %)<br>Repeated falls 19 (21.1 %)<br>Cognitive impairment 18 (20.0 %)<br>Decreased mobility 18 (20.0 %)<br>Hallucinations 10 (11.1 %)<br>Delirium/confusion 7 (7.8 %)<br>Impaired swallow 3 (3.3 %)<br>Stroke 2 (2.2 %)

Authors (year)	Study design	Aim of the study	Setting	Sample size	Statistical analysis	Prevalence of conditions, events, reasons, indication, causes, symptoms or incidents with sample size of study population, n
		of community-dwelling people with Parkinson disease (PD)			department attendances, hospital admissions; length of hospital stay); t-test (age) and χ2 -test (Hoehn and Yahr stage, sex, PD subtypes)	
Fan, C. W., Keating, T., Brazil, E., Power, D., & Duggan, J. (2016)	Retrospective study	To provide a descriptive study of the number of NH transfers to the ED and the patient-oriented outcomes over one year	Emergency department	465 NHRs attending for 802 cases	Student's t test to compare means of continuous variables, Chi-square statistics and one-way ANOVA to compare between groups with different frequencies of attendance	**Clinical presentation n = 802**<br>Unwell adult 235 (29.3 %)<br>Fall 125 (15.5 %)<br>Shortness of breath 91 (11.3 %)<br>Limb problem 72 (9.0 %)<br>Collapsed adult 59 (7.4 %)<br>Urinary problem 42 (5.2 %)<br>Abdominal pain 37 (4.6 %)<br>Chest pain 30 (3.7 %)<br>GI bleeding 21 (2.6 %)<br>Back pain 11 (1.4 %)<br>Diarrhoea and vomiting 10 (1.2 %)<br>Seizure 10 (1.2 %)<br>Miscellaneous 49 (6.1 %)<br>Unknown 10 (1.2 %)
Hillen, J. B., Reed, R. L., Woodman, R. J., Law, D., Hakendorf, P. H., & Fleming, B. J. (2011)	Retrospective longitudinal study	To describe admissions patterns of residential aged care facility (RACF) residents admitted to a major public hospital.	Public general hospital	3.310 admissions of 2.130 individuals	Admission rate ratios, descriptive statistics	**Most prevalent conditions -Admissions n = 1,570**<br>Fractured femur 326 (9.9 %)<br>Ischaemic heart disease (mostly acute) 158 (4.8 %)<br>CHF 154 (4.7 %)<br>Cerebrovascular 91 (3.2 %)<br>Pneumonia 186 (5.6 %)

Authors (year)	Study design	Aim of the study	Setting	Sample size	Statistical analysis	Prevalence of conditions, events, reasons, indication, causes, symptoms or incidents with sample size of study population, n
						UTI 113 (3.4 %)<br>Paralytic ileus with intestinal obstruction 61 (1.8 %)<br>Gastric/duodenal ulcer 24 (0.7 %)<br>Gastro esophageal reflux disease 23 (0.7 %)<br>COPD 150 (4.5 %)<br>Pneumonitis because of solids or liquids 60 (1.8 %)<br>Type 2 diabetes mellitus 78 (2.4 %)<br>Volume depletion 31 (1 0.9 %)<br>Dementia 58 (1.8 %)<br>Delirium (not drug-induced) 30 (0.9 %)<br>Alzheimer's disease 27 (0.8 %)<br><br>**Main categories of conditions - Admissions n = 2,562**<br>Injuries 520 (20.3 %)<br>Cardiovascular disease 420 (16.4 %)<br>Infections 336 (13.1 %)<br>Digestive system disorders/disorders 295 (11.5 %)<br>Respiratory disease 205 (8.0 %)<br>Mental and behavioral disease/disorders 90 (3.5 %)
Givens, J. L., Selby, K., Goldfeld, K. S., & Mitchell, S. L. (2012)	Prospective cohort study	To describe diagnoses and factors associated with hospital transfer in nursing home (NH) residents with advanced dementia	Nursing home	323 NHRs experiencing 74 hospitalizations and 60 ED visits	Descriptive statistics, logistic regression to examine whether or not a hospital transfer occurred for that event	**Hospitalization n = 74**<br>Pneumonia 30 (40.5 %)<br>Other infections 14 (18.9 %)<br>GI bleed 6 (8.1 %)<br>Respiratory distress 5 (6.8 %)<br>Fracture 4 (5.4 %)

Authors (year)	Study design	Aim of the study	Setting	Sample size	Statistical analysis	Prevalence of conditions, events, reasons, indication, causes, symptoms or incidents with sample size of study population, n
						Other 13 (17.6 %)<br><br>**ED visits n = 60**<br>Feeding tube complication 28 (46.7 %)<br>Infections 16 (26.7 %)<br>Fall 8 (13.3 %)<br>Other 8 (13.3 %)
Burke, R. E., Rooks, S. P., Levy, C., Schwartz, R., & Ginde, A. A. (2015)	Retrospective analysis	To identify and describe potentially preventable emergency department (ED) visits by nursing home (NH) residents	Hospitals	3.857 ED visits	Descriptive statistics, comparison of groups using t-tests or Chi-Square tests	**Discharged n = 2,025**<br>Injury (overall) 907 (44.8 %)<br>Infection (overall) 225 (11.1 %)<br><br>Specific diagnoses:<br>Superficial injury, contusion 213 (10.5 %)<br>Open wound 97 (4.8 %)<br>UTI 89 (4.4 %)<br>Other injury 89 (4.1 %)<br>Other GI disorders 81 (4.0 %)<br>Residual codes; unclassified 67 (3.3 %)<br>Other lower respiratory disease 53 (2.6 %)<br>COPD 53 (2.6 %)<br>Abdominal pain 53 (2.6 %)<br>Pneumonia, not caused by TB 45 (2.2 %)<br><br>**Admitted n = 1,832**<br>Injury (overall) 464 (25.3 %)<br>Infection (overall) 420 (22.9 %)<br><br>Specific diagnoses:<br>Pneumonia, not caused by TB 170 (9.3 %)<br>UTI 132 (7.2 %)<br>Residual codes; unclassified 103 (5.6 %)

Authors (year)	Study design	Aim of the study	Setting	Sample size	Statistical analysis	Prevalence of conditions, events, reasons, indication, causes, symptoms or incidents with sample size of study population, n
						Fluid and electrolyte disorder 92 (5.0 %)<br>CHF; non-hypertensive 81 (4.4 %)<br>Nonspecific chest pain 75 (4.1 %)<br>Other lower respiratory disease 66 (3.6 %)<br>GI hemorrhage 60 (3.3 %)<br>Septicemia 57 (3.1 %)<br>Syncope 53 (2.9 %)
Björck, M., & Wijk, H. (2018)	Retrospective descriptive survey	To survey the most ill and frail older persons with cognitive impairment who were transferred from nursing homes to the emergency department, considering reasons for referral, symptoms and actions taken at the hospital	Hospital	588 transfers related to 366 individuals	Descriptive statistics	**Hospital transfers n = 588**<br>Problems with mobility and neuromusculoskeletal and movement-related functions 179 (30.4 %)<br>Sensation of pain 151 (25.7 %)<br>Problems with respiration functions 108 (18.4 %)<br>Problems with functions related to the digestive system 95 (16.2 %)<br>Problems with mental functions 90 (15.3%)<br>Problems with thermoregulatory functions 84 (14.3 %)<br>Problems with cardiovascular functions 51 (8.7 %)<br>Other causes 151 (25.7 %)<br>Referral note missing 85 (14.5 %)

Authors (year)	Study design	Aim of the study	Setting	Sample size	Statistical analysis	Prevalence of conditions, events, reasons, indication, causes, symptoms or incidents with sample size of study population, n
Seeger, I. & Luque R. A. & Hoffmann, F. (2017)	Retrospective cohort study	To compare the utilisation and the underlying diagnoses and characteristics of outpatient emergency care one year before and one year after hospitalisation, separated according to hospital emergency and medical on-call service	Nursing home	1.175 persons	Incidence and proportions of transfers	**On-call service billing code n = 1,140**<br>Cardiovascular diseases 160 (14.0 %)<br>Injuries and poisonings 133 (11.7 %)<br>Infections 80 (7.0 %)<br>Endocrine and metabolic diseases 71 (6.2 %)<br>Diseases of the digestive tract 67 (5.9 %)<br>Psychiatric diseases 59 (5.2 %)<br>Musculoskeletal diseases 58 (5.1 %)<br>Respiratory diseases 56 (4.9 %)<br>Cancer 49 (4.3 %)<br>Cerebrovascular diseases 29 (2.5 %)<br>Diseases of the nervous system 23 (2.0 %)<br>Other 355 (31.1 %)<br><br>**ED visit n = 494**<br>Injuries and poisonings 369 (74.5 %)<br>Musculoskeletal diseases 22 (4.5 %)<br>Endocrine and metabolic diseases 9 (1.8 %)<br>Cardiovascular diseases 9 (1.8 %)<br>Diseases of the digestive tract 9 (1.8 %)<br>Psychiatric diseases 8 (1.6 %)<br>Infections 7 (1.4 %)<br>Respiratory diseases 5 (1.0 %)<br>Diseases of the nervous system 4 (0.8 %)<br>Cerebrovascular diseases 2 (0.4 %)<br>Cancer 1 (0.2 %)<br>Other 50 (10.1 %)

Authors (year)	Study design	Aim of the study	Setting	Sample size	Statistical analysis	Prevalence of conditions, events, reasons, indication, causes, symptoms or incidents with sample size of study population, n
Alrawi, Y. A., Parker, R. A., Harvey, R. C., Sultanzadeh, S. J., Patel, J., Mallinson, R., Potter, J. F., Trepte, N. J., & Myint, P. K. (2013)	Prospective observational study	To identify easily available clinical and laboratory predictors of early death in NH residents admitted to hospital as medical emergencies.	District General Hospital	314 patients constituting 410 emergency episodes	Descriptive statistics, GEE analysis	**Nursing home patients n = 314**<br>Respiratory 117 (37.3 %)<br>GI 52 (16.6 %)<br>Stroke/neurological 51 (16.2 %)<br>Cardiological 38 (12.1 %)<br>UTI 37 (11.8 %)<br>Falls 18 (5.7 %)
Laffon de Mazières, C., Romain, M., Hermabessière, S., Abellan, G., Gerard, S., Castex, A., Krams, T., Vellas, B., & Rolland, Y. (2018)	Retrospective descriptive study	To present the organization of the responsive day hospital dedicated to NH residents and the main characteristics and reasons for sending residents	Responsive day hospital dedicated to NH residents	1.306 NHRs	Descriptive statistics	**Reasons for transfer n = 1,918**<br>Cognitive disorders 336 (17.5 %)<br>Behavioral disorders 297 (15.5 %)<br>Bedsores and slow wound healing 223 (11.6 %)<br>Nutritional status 197 (10.3 %)<br>Neurology 193 (10.1 %)<br>Cardiology 152 (7.9 %)<br>Psychiatry 71 (3.7 %)<br>Assessment of fall 65 (3.4 %)<br>Internal medicine 57 (3.0 %)<br>Transfusion 49 (2.6 %)<br>Urology 44 (2.3 %)<br>Pain and palliative care 33 (1.7 %)<br>Botulinum toxin injection and monitoring 25 (1.3 %)<br>Hematology 22 (1.2 %)<br>Occupational therapy 19 (1.0 %)<br>Physical therapy 15 (0.8 %)<br>Pneumology 14 (0.7 %)<br>Reassessment after acute episode 12 (0.6 %)

Authors (year)	Study design	Aim of the study	Setting	Sample size	Statistical analysis	Prevalence of conditions, events, reasons, indication, causes, symptoms or incidents with sample size of study population, n
						Rheumatology 12 (0.6 %)<br>Dentist 12 (0.6 %)<br>Speech therapist 10 (0.5 %)<br>Endocrinology 9 (0.5 %)<br>Geriatric oncology 7 (0.4 %)<br>Gastroenterology 7 (0.4 %)<br>Other reason 37 (19 %)
Dubucs, X., de Souto Barreto, P., Laffon de Mazieres, C., Lauque, D., Azema, O., Charpentier, S., & Rolland, Y. (2019)	Observational, descriptive, retrospective study	To describe the temporal variability of transfers of NH residents aged 65 years to<br>the ED for traumatic injuries; to characterize their trauma; and<br>to specify the mode of transport to the ED	ED of University Hospital Centre	20.741 patients were included in the study.<br>- 11.879 (57.3 %) were community-dwelling with family<br>- 5.077 (24.5 %) lived in NHs<br>- 3.785 (18.2 %) were community-dwelling alone	Descriptive statistics, comparisons<br>of cumulative frequency were conducted by the chi2 test	**Nursing home residents admitted n = 5,077**<br><br><u>Head and neck trauma n = 2,707</u><br>Head injury 2,245 (82.9 %)<br>Cutaneo-mucous wound 376 (13.9 %)<br>Nose and/or jaw and/or teeth 42 (1.6 %)<br>Eye and/or margin of the eye 29 (1.1 %)<br>Cervical spine fracture 14 (0.5 %)<br>Nerve, muscle or ligament lesion 1 (0.0 %)<br><br><u>Lower extremity trauma n = 1,429</u><br>Limb fracture 871 (60.9 %)<br>Cutaneo-mucous wound 487 (34.1 %)<br>Sprains, dislocations 63 (4.4 %)<br>Nerve, muscle or ligament lesion 8 (0.6 %)<br><br><u>Upper extremity trauma n = 625</u><br>Limb fracture 372 (59.5 %)<br>Cutaneo-mucous wound 204 (32.6 %)<br>Sprain, dislocation 42 (6.7 %)<br>Nerve, muscle or ligament lesion 7 (1.2 %)<br><br><u>Trunk trauma n = 316</u><br>Cutaneo-mucous wound 127 (40.2 %)

Authors (year)	Study design	Aim of the study	Setting	Sample size	Statistical analysis	Prevalence of conditions, events, reasons, indication, causes, symptoms or incidents with sample size of study population, n
						Spine or pelvic fracture 151 (47.8 %)<br>Costovertebral fracture 34 (10.8 %)<br>Nerve, muscle or ligament lesion 4 (1.2 %)
Manckoundia, P., Menu, D., Turcu, A., Honnart, D., Rossignol, S., Alixant, J. C., Sylvestre, F. H., Bailly, V., Dion, M., & Putot, A. (2016)	Prospective multicenter observational study	To determine the rate of inappropriate admissions to emergency departments (EDs) and to identify determinants of these admissions	Emergency departments and nursing homes	1.000 NHRs admitted to EDs	Descriptive statistics, chi-square test or Fisher exact test for bivariate analysis	**Nursing home residents n = 1,000**<br>Symptoms, signs, and results not classified elsewhere 390 (39.0 %)<br>External causes of morbidity 245 (24.5 %)<br>Disease of the circulatory system 97 (9.7 %)<br>Traumatic lesions, poisoning, and other external causes 64 (6.4 %)<br>Disease of the digestive system 54 (5.4 %)<br>Disease of the respiratory system 29 (2.9 %)<br>Mental and behavior disorders 23 (2.3 %)<br>Disease of the blood and of the immune system 22 (2.2 %)<br>Disease of the nervous system 15 (1.5 %)<br>Certain infectious and parasitic disease 11 (1.1 %)<br>Disease of the osteoarticular system, muscles 11 (1.1 %)<br>Disease of the skin 10 (1.0 %)<br>Disease of the genitourinary system 8 (0.8 %)<br>Factors affecting the state of health 8 (0.8 %)<br>Endocrine, nutritional, and metabolic diseases 6 (0.6 %)<br>Disease of the eye 3 (0.3 %)<br>Disease of the ear 2 (0.2 %)<br>Congenital malformations and chromosomal anomalies 1 (0.1 %)<br>Certain disorders originating in the perinatal period 1 (0.1 %)

Authors (year)	Study design	Aim of the study	Setting	Sample size	Statistical analysis	Prevalence of conditions, events, reasons, indication, causes, symptoms or incidents with sample size of study population, n
						Tumors 0 (0 %)
Unroe, K. T., Caterino, J. M., Stump, T. E., Tu, W., Carnahan, J. L., Vest, J. R., Sachs, G. A., & Hickman, S. E. (2020)	OPTIMISTIC clinical demonstration project	To describe differences in transfer events that result in treatment in the hospital versus emergency department (ED) only	Emergency department and long-stay nursing facility	867 residents	Transfer events that resulted in treatment in ED versus hospitalization were compared using t-tests and chi-square tests. A regression model was used to assess associations between hospital admission and transfer characteristics	**Patients n = 686**<br>Fall with or without injury 98 (14.3 %)<br>Pneumonia 65 (9.5 %)<br>Sepsis/urosepsis 55 (8.0 %)<br>UTI without sepsis 39 (5.7 %)<br>Dementia-related behaviors 34 (5.0 %)<br>Hip fracture/other fracture 34 (5.0 %)<br>Heart failure 21 (3.1 %)<br>Seizures 14 (2.0 %)<br>Stroke or TIA 14 (2.0 %)<br>COPD exacerbation 12 (1.7 %)<br>Laceration 12 (1.7 %)<br>Other 224 (32.7 %)
Harrison, J. M., Agarwal, M., Stone, P. W., Gracner, T., Sorbero, M., & Dick, A. W. (2021)	Cross-sectional observational study	To evaluate the association between integration and (1) all-cause hospital transfers and (2) hospital transfers due to infection.	Hospital and nursing home	143,223 NH residents	Logistic regression models were used to estimate relationships between integration intensity and all-cause hospital transfer and transfer due to infection	**All residents n = 143,223**<br>Hospital transfers per resident per year 0.348<br>Percent of transfers due to infection (any) 39.8 %<br>Percent of transfers due to respiratory infection 9.4 %<br>Percent of transfers due to UTI 6.7 %<br>Percent of transfers due to sepsis 21.9 %
Griffey, R. T., Schneider, R. M., Adler, L., & Todorov, A. (2021)	Retrospective observational study	To describe all-cause harm among patients from PA/LTC settings seen in the ED	Emergency department	423 patients from PA/LTC	Descriptive statistics	**adverse events n = 189**<br>Patient Care 73 (38.6 %)<br>Medication 64 (33.9 %)<br>Healthcare-associated infections 30 (15.9 %)<br>Device 10 (5.3 %)<br>Surgery/procedural 8 (4.2 %)

Authors (year)	Study design	Aim of the study	Setting	Sample size	Statistical analysis	Prevalence of conditions, events, reasons, indication, causes, symptoms or incidents with sample size of study population, n
						Care coordination 4 (2.1 %)<br><br>Patient Care n = 73<br>Fall 35 (47.9 %)<br>Pressure ulcer 9 (12.3 %)<br>Other 8 (11 %)<br>Traumatic injury 8 (11 %)<br>Aspiration 4 (5.5 %)<br>Hypoxia 3 (4.1 %)<br>Glycemic event 3 (4.1 %)<br>MI 1 (1.4 %)<br>Airway-related event 1 (1.4 %)<br>Transfusion reaction 1 (1.4 %)
Pulst, A., Fassmer, A. M., & Schmiemann, G. (2021)	Multi-center observational study	To analyze the characteristics of hospital transfers from nursing homes (NHs) focused on contacts to physicians, family members and legal guardians prior to a transfer	Nursing homes	535 unplanned hospital transfers from 802 NH residents	Descriptive statistics	**Total unplanned transfers n = 535**<br>Deterioration of health (fever, infection, exsiccosis) 188 (35.1 %)<br>Fall/accident/injury 179 (33.5 %)<br>Others (GI symptoms) 59 (11.0 %)<br>Psychiatric/neurologic disorders (challenging behavior) 38 (7.1 %)<br>Complications with catheter/tube (blood in urine) 38 (7.1 %)<br>Pain, not fall-induced 33 (6.2 %)<br><br>**hospital admissions n = 334**<br>Deterioration of health (fever, infection, exsiccosis) 16 (48.5 %)<br>Fall/accident/injury 76 (22.8 %)<br>Others (GI symptom) 39 (11.7 %)

Authors (year)	Study design	Aim of the study	Setting	Sample size	Statistical analysis	Prevalence of conditions, events, reasons, indication, causes, symptoms or incidents with sample size of study population, n
						Psychiatric/neurologic disorders (challenging behavior) 31 (9.3 %)<br>Complications with catheter/tube (blood in urine) 6 (1.8 %)<br>Pain, not fall-induced 20 (6.0 %)<br><br>**ED visits n = 195**<br>Fall/accident/injury 101 (51.8 %)<br>Complications with catheter/tube (blood in urine) 32 (16.4 %)<br>Deterioration of health (fever, infection, exsiccosis) 22 (11.3 %)<br>Others (e.g. gastrointestinal symptoms, bleedings) 20 (10.3 %)<br>Pain, not fall-induced 13 (6.7 %)<br>Psychiatric/neurologic disorders (challenging behavior) 7 (3.6 %)
Nemiroff, L., Marshall, E. G., Jensen, J. L., Clarke, B., & Andrew, M. K. (2019)	Mixed methods study	To estimate the prevalence of, and adherence to, "no transfer to hospital" ADs in LTC, and to explore the circumstances leading to transfers against previously expressed directives	Nursing home	748 resident charts and Emergency Health Services (EHS) database notes	Descriptive statistics, qualitative data	**Emergency calls for residents n = 284**<br>Transfers to hospital n = 210<br>Injury 85 (40.6 %)<br>Respiratory 31 (14.8 %)<br>Sick – other 29 (13.8 %)<br>GI 22 (10.5 %)<br>Neurologic 19 (9.1 %)<br>Cardiac 12 (5.7 %)<br>Scheduled/planned 6 (2.9 %)<br>Epistaxis 3 (1.4 %)<br>Psychiatric 3 (1.4 %)<br><br>**Management on-site n = 74**

Authors (year)	Study design	Aim of the study	Setting	Sample size	Statistical analysis	Prevalence of conditions, events, reasons, indication, causes, symptoms or incidents with sample size of study population, n
						Sick – other 25 (33.8 %)<br>Injury 18 (24.3 %)<br>Respiratory 10 (13.5 %)<br>Not recorded 6 (8.1 %)<br>Cardiac 5 (6.8 %)<br>Neurologic 5 (6.8 %)<br>GI 3 (4.1 %)<br>Epistaxis 1 (1.4 %)<br>Scheduled/planned 1 (1.4 %)
Heinold, S., Fassmer, A. M., Schmiemann, G., & Hoffmann, F. (2021)	Multicenter observational study	To analyze underlying diagnoses, characteristics and performed medical procedures	Emergency department, nursing home	161 unplanned ED visits	Descriptive statistics	**Nursing home residents n = 161**<br>Trauma 95 (59.0 %)<br>Catheter and probe problems 17 (10.6 %)<br>Altered mental state 16 (9.9 %)<br>Other 9 (5.6 %)<br>GI system 6 (3.7 %)<br>Cardiovascular system 6 (3.7 %)<br>Infection 5 (3.1 %)<br>Urogenital system 5 (3.1 %)<br>Central nervous system 1 (0.6 %)<br>Respiratory system 1 (0.6 %)

Authors (year)	Study design	Aim of the study	Setting	Sample size	Statistical analysis	Prevalence of conditions, events, reasons, indication, causes, symptoms or incidents with sample size of study population, n
Rolland, Y., Mathieu, C., Tavassoli, N., Berard, E., Laffon de Mazières, C. et al. (2021)	Multicenter, observational, case-control study	To determine the factors associated with the potentially inappropriate transfer of nursing home (NH) residents to emergency departments (EDs) and to compare hospitalization costs before and after transfer of individuals addressed inappropriately vs those addressed appropriately	Emergency department	1037 NH residents	Mixed logistic regression to determine factors associated independently with potentially inappropriate transfers	**Nursing home residents n = 1,037**<br>Fall with suspicion of fracture (n = 1,037) 388 (37.4 %)<br>Other reason (n = 1,037) 309 (29.8 %)<br>Dyspnea (n = 1,036) 241 (23.3 %)<br>Deterioration of general condition (n = 1,035) 212 (20.5 %)<br>Neurologic symptoms (n = 1,035) 157 (15.2 %)<br>Abdominal pain (n = 1,037) 126 (12.2 %)<br>Fever (n = 1,034) 124 (12.0 %)<br>Bleeding (n = 1,035) 83 (8.0 %)<br>Behavior disturbances (n = 1,035) 77 (7.4 %)<br>Thoracic pain (n = 1,036) 63 (6.1 %)<br>[Deviating n due to missings]
Grimm, F., Hodgson, K., Brine, R., & Deeny, S. R. (2021)	Retrospective analysis	To examine trends in the number of hospital admissions for care home residents during the first months of the COVID-19 outbreak	Hospital	257,843 residents between 20 January 2020 and 28 June 2020 compared to admissions during the corresponding period in 2019 252,432 residents	Descriptive statistics	**Admissions per 100 residents per year**<br>COVID-19 13.18<br>Respiratory system 9.45<br>Injury, poisoning 7.52<br>Not elsewhere classified 4.28<br>Infectious, parasitic 2.95<br>Genitourinary system 2.87<br>Circulatory system 2.53<br>Digestive system 2.47<br>Endocrine, nutritional, metabolic 1.29<br>Musculoskeletal, connective 1.15<br>Nervous system 1.03<br>Mental, behavioral 0.82<br>Skin, subcutaneous tissue 0.75<br>Other/unknown 0.63<br>Blood, blood-forming organs 0.3

Authors (year)	Study design	Aim of the study	Setting	Sample size	Statistical analysis	Prevalence of conditions, events, reasons, indication, causes, symptoms or incidents with sample size of study population, n
						Neoplasms 0.24<br>Factors influencing health status 0.22<br><br>**Admission rate per 100 residents per year potentially avoidable**<br>Pneumonia 4.86<br>Fractures and sprains 3.31<br>Food and liquid pneumonitis 1.98<br>Urinary tract infections 1.81<br>Acute LRTIs 1.37<br>Chronic LRTIs 0.82<br>Intestinal infections 0.31<br>Diabetes 0.44<br>Pressure sores 0.07<br>Food and drink issues 0.06
Axon, R. N., Gebregziabher, M., Craig, J., Zhang, J., Mauldin, P., & Moran, W. P. (2015)	Retrospective cohort study	To describe the frequency of NH transfers for ambulatory care-sensitive conditions (ACSCs) and estimates associated expenditures	Emergency department	20,867 NH patients	Descriptive statistics, generalized linear models were used to estimate the costs attributable to treating ACSCs	**Patients n = 4,680**<br>Kidney/urinary infection 2,118 (45.3 %)<br>Dehydration/volume depletion 1,159 (24.8 %)<br>Bacterial pneumonia 428 (9.1 %)<br>Cellulitis 334 (7.1 %)<br>Other conditions 200 (4.3 %)<br>Convulsions 188 (4.0 %)<br>Iron deficiency anemia 128 (2.7 %)<br>Gastroenteritis 56 (1.2 %)<br>Severe ear, nose, & throat infections 37 (0.8 %)<br>Hypoglycemia 32 (0.7 %)

Authors (year)	Study design	Aim of the study	Setting	Sample size	Statistical analysis	Prevalence of conditions, events, reasons, indication, causes, symptoms or incidents with sample size of study population, n
Cummings, G. G., McLane, P., Reid, R. C., Tate, K., Cooper, S. L., Rowe, B. H., Estabrooks, C. A., Cummings, G. E., Abel, S. L., Lee, J. S., Robinson, C. A., & Wagg, A. (2020)	Prospective observational study	To describe characteristics of residents transferred, factors related to decisions during transfer, care received in emergency medical services (EMS), ED settings, outcomes on return to LTC, and times of transfer segments along the transition	Emergency department	637 transitions from residents to an ED	Descriptive statistics	**Transfers n = 637**<br>Falls 171 (26.8 %)<br>Sudden change in condition 150 (23.5 %)<br>Shortness of breath 126 (19.8 %)<br>Nausea / vomiting / diarrhea 45 (7.1 %)<br>General malaise (weakness) 35 (5.5 %)<br>Cough with congestion 32 (5.0 %)<br>Constipation (bloating/abdominal pain) 30 (4.7 %)<br>Family / friend caregiver request 29 (4.6 %)<br>GI bleed 27 (4.2 %)<br>Leg pain /cramps / swelling 24 (3.8 %)<br>Missing n=11
Hsiao, C. J., & Hing, E. (2014)	Observational study	To examine emergency department (ED) visits by nursing home (NH) residents aged 65 and over	Emergency department	4,970 ED visits were made by NH residents aged 65 and over	Cross-sectional analyses were conducted on patient characteristics, diagnosis, procedures received, and triage status, descriptive statistics	**ED visits n = 4,970**<br>Any ACSC 14.6 %<br>Kidney/UTI 4.9 %<br>CHF 3.2 %<br>Dehydration 2.2 %<br>COPD 1.8 %<br>Cellulitis 1.4 %<br>Hypertension 1.0 %<br>Hypoglycemia 0.8 %<br>Angina 0.7 %<br>Grand mal seizure disorders 0.7 %<br>Diabetes 0.6 %<br>Severe ear, nose, and throat infections 0.4 %<br>Bacterial pneumonia 0.2 %<br>Asthma 0.1 %<br>[Absolute numbers not reported in original paper]

Authors (year)	Study design	Aim of the study	Setting	Sample size	Statistical analysis	Prevalence of conditions, events, reasons, indication, causes, symptoms or incidents with sample size of study population, n
Morphet, J., Innes, K., Griffiths, D. L., Crawford, K., & Williams, A. (2015)	Retrospective review of ED records	To describe the characteristics of residents transferred from residential aged care facilities to EDs, and to evaluate the appropriateness and cost of these presentations	Emergency department	408 resident transfers analysed	Descriptive statistics	**Resident transfers n = 408**<br>Falls 74 (18.1 %)<br>Shortness of breath 56 (13.7 %)<br>Cardiac complaints (including chest pain and arrhythmias) 38 (9.3 %)<br>Altered conscious state 33 (8.1 %)<br>Being generally unwell 33 (8.1 %)<br>Abdominal pain 24 (5.9 %)<br>Renal problem 19 (4.6 %)<br>Pain 11 (2.7 %)
Hathaway, E. E., Carnahan, J. L., Unroe, K. T., Stump, T. E., O'Kelly Phillips, E., Hickman, S. E., Fowler, N. R., Sachs, G. A., & Bateman, D. R. (2021)	Secondary data analysis of OPTIMISTIC study	To characterize pretransfer on-site nursing home (NH) management, transfer disposition, and hospital discharge diagnoses of long-stay residents transferred for behavioral concerns	Emergency department	355 transfers of residents	Descriptive statistics	**Transfers n = 355**<br>Change in mental status 148 (41.6 %)<br>Behavioral symptoms 122 (34.4 %)<br>Confusion or worsening cognitive function 101 (28.5 %)<br>Fever 32 (9.0 %)<br>Shortness of breath/high respiratory rate 31 (8.7 %)<br>Tachycardia 26 (7.3 %)<br>Infection 26 (7.3 %)<br>Vomiting 18 (5.1 %)<br>Malaise 17 (4.8 %)<br>Change in appetite 17 (4.8 %)<br>Hypotension 16 (4.5 %)<br>Hypertension 16 (4.5 %)<br>Unresponsiveness 15 (4.2 %)<br>Critical lab value 15 (4.2 %)<br>Pain 14 (3.9%)<br>Hypoxia 14 (3.9%)<br>Falls with injury 14 (3.9 %)

Authors (year)	Study design	Aim of the study	Setting	Sample size	Statistical analysis	Prevalence of conditions, events, reasons, indication, causes, symptoms or incidents with sample size of study population, n
						New neurologic weakness 12 (3.4 %)<br>Suicidal ideation 11 (3.1 %)<br>Falls without obvious injury 11 (3.1 %)<br>Seizure 8 (2.3 %)<br>Cough 7 (2.0 %)<br>Urinary symptoms or incontinence 6 (1.7 %)<br>Fluid imbalance 6 (1.7 %)<br>Abdominal pain 5 (1.4 %)<br>Limb swelling 4 (1.1 %)<br>Depressive affect 4 (1.1 %)<br>Diarrhea 2 (0.6 %)<br>Chest pain 2 (0.6 %)<br>Suspected soft tissue injury 1 (0.3 %)<br>Loss of consciousness 1 (0.3 %)<br>Head trauma 1 (0.3 %)<br>Dizziness 1 (0.3 %)<br>Bradycardia 1 (0.3 %)<br>Bloody stool1 (0.3 %)<br>Bleeding (other than GI) 1 (0.3 %)<br>Anemia 1 (0.3 %)<br>Other (please specify) 15 (4.2 %)

Authors (year)	Study design	Aim of the study	Setting	Sample size	Statistical analysis	Prevalence of conditions, events, reasons, indication, causes, symptoms or incidents with sample size of study population, n
Vossius, C. E., Ydstebø, A. E., Testad, I., & Lurås, H. (2013)	Retrospective observational study	To investigate hospital referrals of nursing home patients in the municipality of Stavanger, Norway and identify the number of inappropriate referrals and costs	Hospital referrals from nursing homes	359 referrals	Descriptive statistics	**Referrals n = 359**<br>Falls 81 (22.6 %)<br>Infections 56 (15.6 %)<br>Respiratory problems 43 (12.0 %)<br>Cerebral symptoms 42 (11.7 %)<br>GI tract 35 (9.7 %)<br>Cardiac symptoms 26 (7.2 %)<br>General decline 16 (4.5 %)<br>Psychiatric problems 9 (2.5 %)<br>Procedures (urinary catheterization, care of stomach probes) 23 (6.4 %)<br>Other medical conditions 28 (7.8 %)
Ayaz, S. I., Haque, N., Pearson, C., Medado, P., Robinson, D., Wahl, R., Zervos, M., & O'Neil, B. J. (2014)	Retrospective chart review	To investigate the ED presentation, course, management and outcomes in patients admitted through the ED with nursing home-acquired pneumonia	Emergency department	296 NH residents with pneumonia	Descriptive statistics, univariate analysis	**Patients admitted n = 296**<br>(missing absolute numbers not reported in original paper)<br><u>Admission diagnosis</u><br>Pneumonia 237 (80.1 %)<br>Sepsis 20.9 %<br>UTI 17.2 %<br>Dehydration 10.1 %<br>CHF 11.8 %<br>Acute renal failure/renal insufficiency 9.5 %<br>Acute respiratory failure 7.1 %<br>COPD 6.8 %<br>Atrial fibrillation 4.5 %<br><br><u>Comorbidities</u><br>Hypertension 82.8 %<br>Dementia 55.1 %<br>CHF 42.2 %<br>Stroke/TIA 39.2 %

Authors (year)	Study design	Aim of the study	Setting	Sample size	Statistical analysis	Prevalence of conditions, events, reasons, indication, causes, symptoms or incidents with sample size of study population, n
						COPD 30.7 %<br>Renal disease30.7 %<br>Diabetes mellitus 26.4 %<br>Alzheimer's 22.3 %<br>Liver disease 1.7 %
Kim, K., Lee, D. H., Yune, H. Y., Wee, J. H., Kim, D. H., Kim, E. C., Lim, J. Y., & Choi, S. P. (2019)	Retrospective multicentre, observational study	To investigate the reasons of transfers from long-term care hospitals (LTCHs) to emergency departments (EDs) of university hospitals in geriatric patients and to categorize the avoidable causes of these transfers	Emergency department	1,131 NH residents	Descriptive statistics	**Transfers from NHs n = 1,131**<br>Cerebrovascular disorders 462 (40.8 %)<br>Dementia 169 (14.9 %)<br>Orthopedic injuries 212 (10.7 %)<br>Malignancies 91 (8.1 %)<br>End-stage kidney disease 74 (6.5 %)<br>Generalized weakness 79 (7.0 %)<br>Other trauma 38 (3.4 %)<br>CHF 24 (2.1 %)<br>COPD 16 (1.4 %)<br>Vegetative state 15 (1.3 %)<br>Others 40 (3.5 %)

ACSC Ambulatory care sensitive condition, CHF congestive heart failure, CLABSI central line associated blood stream infection, CNS [not explained in original research], COPD chronic obstructive pulmonary disease, COVID-19 Coronavirus disease 2019, ED Emergency department, ERV emergency resuscitation vehicle, GI gastrointestinal, GU [not explained in original research], LRTI lower respiratory tract infection, MI myocardial infarct, NH Nursing home, TIA transient ischemic attack, UTI urinary tract infection, UVI [not explained in original research].

c. Synthesis RQ2

Table A11: Data synthesis of causes for the initiation of medical services (prevalence)

		Type of emergency medical service			
	**Main categories of reason/condition**	**Contact with on-call medical service**	**Contact with emergency medical service**	**Transfer to Emergency Department**	**Transfer to hospital**
Reason for transfer with a prevalence of over 10 % of contacts within a study population (n=number of studies reporting condition)	Orthopaedic	Injuries and poisonings (1)	Injury (1)<br>Injury (1)*	Fall with/ without injury (11)<br>Traumatic injury (7)<br>Ortho. without fracture (2)<br>Ortho. with fracture (2)<br>Problems with mobility (1)	Injury (3)<br>Fall with/without injury (2)<br>Fall with/ without injury (1)**
	Cardiovascular	Cardiovascular symptom (1)	-	Congestive heart failure (1)<br>Cardiovascular symptoms (1)	Cardiovascular symptoms (2)
	Neurologic	-	-	Neurologic symptoms (3)	Stroke (1)<br>Neurologic symptoms (1)
	Respiratory	-	Respiratory symptoms (1)	Respiratory symptoms (11)<br>Pneumonia (2)	Respiratory symptoms (2)<br>Pneumonia (1)
	Deterioration of health including fever, dehydration and infections		Fever or exsiccosis (1)	Urinary tract infection (4)<br>Sepsis (3)<br>Sick, fever or exsiccosis (3)<br>Any infections (3)<br>Dehydration (2)<br>Fever (1)<br>Sudden change in condition (1)<br>Pain (1)<br>Problems with thermoregulatory functions (1)	Any infection (3)<br>Sick, fever or exsiccosis (3)<br>Sepsis (1)<br>Any infection (1)*<br>Sepsis (1)*
	Care related reasons	-	-	Complications with urinary catheter or feeding tube (3)<br>Pressure ulcer (1)<br>Ambulatory care sensitive condition (1)***	Pressure ulcer (1)<br>Nutritional status (1)

	Gastrointestinal		-	Gastrointestinal symptoms (3)	Gastrointestinal symptoms (2)
	Behaviour change	-	-	Worsening cognitive function (2)*<br>Change in mental status (1)<br>Change in mental status (1)*<br>Behavioural symptoms (1)*<br>Dementia (1)	Cognitive disorders (1)<br>Behavioural disorders (1)
	Other conditions	-	-	Complex poly-pathology (2)	-

*: Study population consisting solely of residents with dementia

**: Study population consisting solely of residents with Parkinson disease

***: Ambulatory care sensitive condition: health conditions for which adequate management, treatment and interventions delivered in the ambulatory care setting could potentially prevent hospitalization (Axon et al., 2015).

Table A12: Data synthesis of main causes for the initiation of medical services (admission rate)

Type of transfer	Type of statistic	Conditions/ reason (rate)	Number of studies
Potentially avoidable hospital transfer	Admission rate per 100 residents per year	Pneumonia (4.86)<br>Fractures and sprains (3.31)<br>Urinary tract infections (1.81)<br>Acute lower respiratory tract infections (1.37)<br>Food and liquid pneumonitis (1.98)<br>Chronic lower respiratory tract infections (0.82)<br>Intestinal infections (0.31)<br>Diabetes (0.44)<br>Food and drink issues (0.06)<br>Pressure sores (0.07)	1

4. Results RQ3 –factors influencing the use of emergency medical care

a. PRISMA Flowchart RQ3

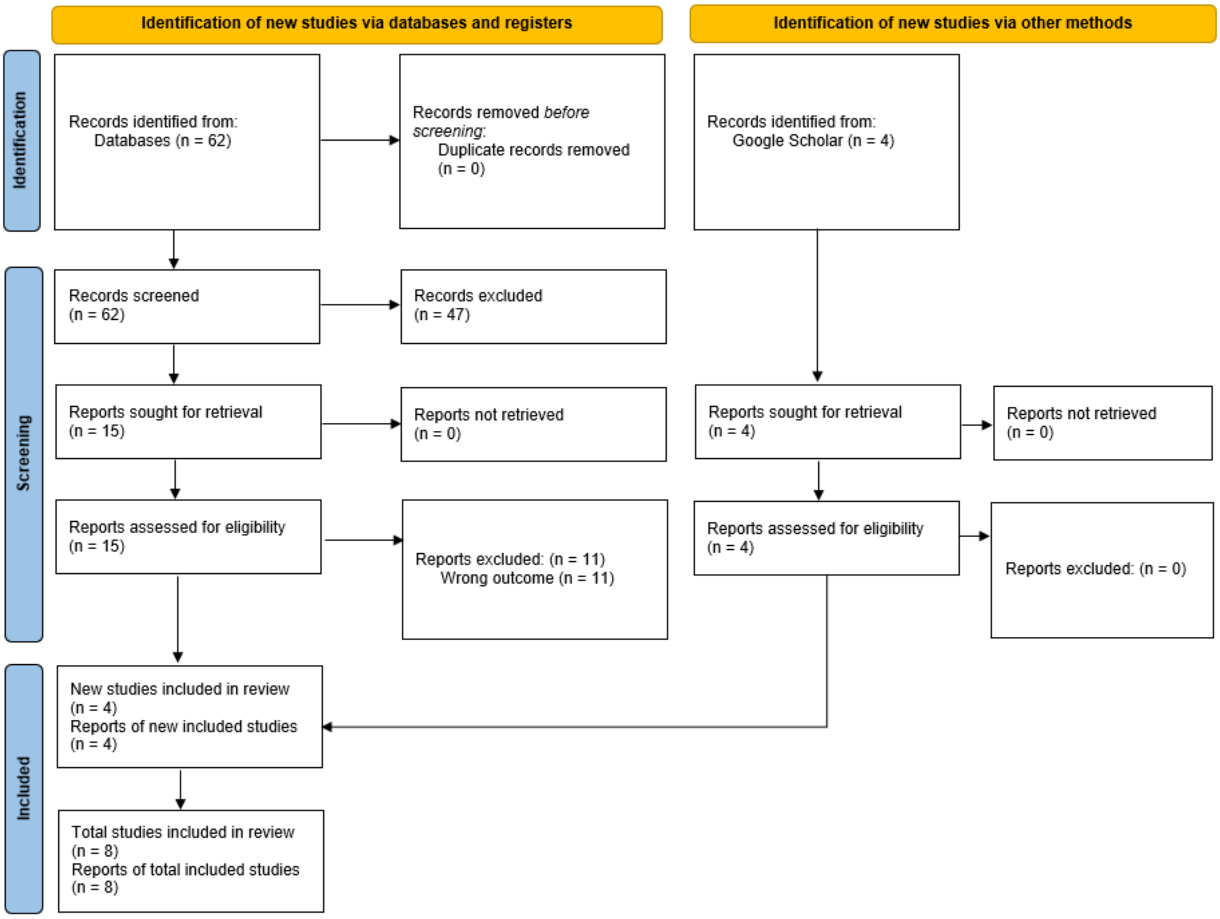

Figure A3: PRISMA flowchart factors influencing use of emergency medical services

b. Study characteristics RQ3

Table A13: Study characteristics factors influencing use of emergency medical services

First author, year, country	Research question	Methods of synthesis	Nr and type of included studies	Main results/outcomes
Arendts et al., 2013, Australia	To systematically review the qualitative literature regarding decisions to transfer people from RACF to ED, and examine the major influences on that decision and how the decision is made.	Systematic literature review with development of key domains which were further grouped into major themes. (No reference given for synthesis methodology)	Primary qualitative studies (n = 11)	**Two major themes with seven domains in total:**<br>a. Transfer with an expectation of better outcomes for the resident - "resident dominant" theme<br>b. Transfer with no expectation of better outcomes for the resident - "resident subordinate" theme
Brucksch et al., 2018, Germany	To estimate the incidence and prevalence of ED visits in nursing home residents (NHRs), focusing on age-specific and sex-specific patterns.	Systematic literature review with descriptive synthesis (no reference given for synthesis methodology).	Cross-sectional studies, cross-sectional follow-up studies, retrospective cohort studies, retrospective chart review and survey (n = 7)	• Influence of sex<br>• Influence of age<br>• Reasons for admission
Dwyer et al., 2014, Australia	To summarise current evidence relating to patterns of presentation, clinical consequences and health system utilisation surrounding emergency hospital transfer of RACF residents.	Systematic literature review with synthesis based on systematic description and analysis of key concepts (Slavin, ref. 18, 19 in appendix.)	Medical record reviews combined with hospital administrative data, patient interviews and studies based on data from national health agencies (n = 83)	• Common reasons for transfer of RACF residents<br>• Comorbidities of transferred residents<br>• Hours of arrival<br>• Clinical consequences<br>• Health system utilisation
Laging et al., 2015, Australia	To provide a substantial description of the decision-making processes that take place prior to the transfer of a NH resident to the ED, specifically from a NH staff perspective.	Systematic literature review with meta-synthesis following the Joanna Briggs Institute's guidelines	Primary qualitative studies (n = 17)	• Lack of consensus of the nursing home role<br>• Limited skills and confidence to manage the resident onsite<br>• Limited access to multidisciplinary services and resource<br>• Barriers to NH staff participation in the decision process<br>• Challenges advocating on behalf of the resident

First author, year, country	Research question	Methods of synthesis	Nr and type of included studies	Main results/outcomes
Lemoyne et al., 2019, Belgium	To define the characteristics of ED transfers of NH residents, to describe definitions of appropriateness and to identify factors associated with a reduction in inappropriate transfers.	Systematic literature review with data extraction on three categories (outcomes of transfers, appropriateness, factors influencing the proportion of appropriate transfers).	RCT, narrative reviews, systematic reviews, experimental studies, qualitative studies, observational studies (n = 77)	• Outcomes of transfer<br>• Appropriateness of transfer<br>• Common reasons for transfer<br>• Factors influencing the proportion of appropriate transfers
O'Neill et al., 2015, Australia	To obtain a greater understanding of the perspectives of the nurses involved with resident transfers, and to describe nursing home nurses' experiences and overall perceptions around ED transfers.	Systematic literature review following the Joanna Briggs Institute approach to qualitative systematic review, using meta-aggregation.	Primary qualitative studies (n = 7)	**Three meta-synthesis statements:**<br>1. The decision to transfer is complex; nurses require clinical knowledge, skills and resources to assess and manage the deteriorating resident.<br>2. Families maintaining a position of power and this underlies nurses' actions and interactions. Communication issues can cause delays and problems. Nursing home nurses use persuasive and targeted communication techniques to manage and direct possible transfer situations.<br>3. Ambiguity, strained relationships and nursing home nurses' negative perceptions of residents' experiences around hospitalisation create conflict and uncertainty.
Pulst et al., 2019, Germany	To summarise family members' experience and perceived involvement in the decision to transfer a nursing home resident (NHR) to hospital.	Systematic literature review following the Joanna Briggs Institute approach to qualitative systematic review. (Lockwood 2017)	Qualitative and mixed methods primary studies (n = 10)	**Five synthesised findings:**<br>1. Transfer decision is affected by family members' judgement of quality of NH care (nursing home related factors)<br>2. Transfer decision is affected by family members' judgement on quality of hospital care (hospital related factors)<br>3. Perceived severity of clinical situation effects the transfer decision (family related and resident related factors)<br>4. Knowing, accepting and upholding resident wishes are challenges for family members (family-related and resident-related factors)

First author, year, country	Research question	Methods of synthesis	Nr and type of included studies	Main results/outcomes
				5. The extent of family members' involvement in treatment and transfer decisions vary (forms of family involvement).
Trahan et al., 2016, Canada	To identify characteristics of avoidable or unnecessary transitions of nursing home (NH) residents to Eds, and factors influencing decision-making by NH clinicians (including nurses and physicians), NH residents and their family members when applicable, to transfer residents to EDs for care.	Systematic literature review with data synthesis using content analysis to build dimensions of factors contributing to transfer decisions and finding commonalities in definitions and descriptions of "avoidable" transfers to ED. No reference given for synthesis methodology.	Quantitative, qualitative and mixed methods primary studies, reviews (n = 19)	**Results regarding factors influencing decision-making by NH clinicians:**<br>• Nursing factors<br>• Physician/NP factors<br>• Facility/resource factors<br>• NH residents / family factors<br>• Health system factors

ED: Emergency department; NH: Nursing home; RACF: Residential aged care facilities

c. Synthesis RQ3

Table A14: Data synthesis factors influencing use of emergency medical services

Physician factors	Nurse factors	Resident factors	Family factors	Organisation	Health System factors
• Availability<br>• End-of-life decision-making / lack of knowledge of ACP or residents' wishes / surrendering to families wishes<br>• Liability related to decision-making and treatment / fear of litigation<br>• Specialised skill and knowledge of geriatric medicine<br>• Positive communication and professional relationships with NH staff<br>• Quality care-related physician visits in the NH	• Knowledge/competence related to recognizing and managing acute changes in the NH resident<br>• Lack of confidence in own competencies or other staff<br>• Quality of care, inadequate planning of care<br>• Communication barriers between nursing and care staff and physicians<br>• Positive relationship with NH resident, family and physician<br>• Knowledge / awareness of NH residents' needs and preferences or care plans<br>• Lack of knowledge of outpatient care options<br>• Overall attitude towards care in hospital versus care in nursing home<br>• Fear of reprimands of ED staff<br>• Personal liability, fear of taking over responsibility, risk-adverse behaviour of staff<br>• Perceived and real workload and safety<br>• Lack of formal role of NH nurses in decision-making process	• Advance directives not in place or not followed<br>• Residents' wishes (alter) in acute situation<br>• Characteristics of residents' condition or the acute situation (co-morbidities and reasons for admission, acuity/severity)<br>• Previous admissions to ED or hospital<br>• Anticipated adverse effects of hospital stay on resident	• Families preferences differ from residents' or health care professionals.<br>• Families exert pressure to achieve the desired decision.<br>• Families believe care in hospital is superior to NH and in residents' best interest.<br>• Nature or the relationship with NH staff and/or physician influences preferences and decision-making.<br>• Families do not accept ACPs or are unaware of residents' wishes and feel insecure in their role as decision-maker, prefer that decision is made by health care professionals.<br>• Families do not understand residents' condition (e.g. end-of-life).	• Role perception of NH in provision of (medical) care unclear or policy to treat/not-treat.<br>• Lack of resources and treatment options onsite (competent and sufficient staff, equipment)<br>• Medical care impairs care for other residents.<br>• Institutional liability issues<br>• Financial burden<br>• (Structured) communication between organisations/facilities<br>• Availability / access to primary care onsite or in outpatient facilities, vicinity to ED<br>• Advance care planning policy in the NH	• Bureaucratic issues/ requirements limiting care in NH /defining NH role<br>• Government restricting nature and scope of practice of NH staff<br>• Relationships between NH and acute care facilities and their personnel<br>• Access / availability to community resources (depending on the structure or the health care system)

NH: Nursing home; ED: Emergency department; ACP: Advance care planning

5. Summary of results regarding a new nursing role in long-term care

Complex care situations were reported for three different domains (Figure A4). Whereas specific conditions or care needs as in wound care or infections may only target a smaller group of residents in long-term care or a smaller time frame, the enablement of autonomy and the management of resources were also perceived as complex care situations. The latter situations suggest they are possibly always there, include all residents in nursing homes and are dependent on factors in nursing education as well as the health care system, making these needs less likely to be addressed by implementing a new nursing role in long-term care.

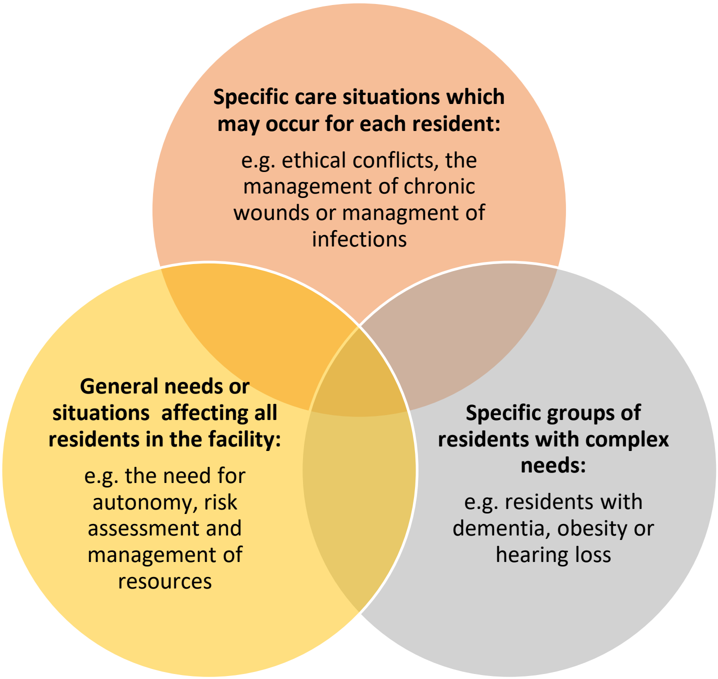

Figure A4: Domains of complex needs

Causes for complexity are mainly due to illnesses, multimorbidity or cognitive limitations. Furthermore, aspects of interprofessional collaboration between physicians or the involvement of family members were perceived as causes for the complex nature. Relevant to the new nursing role are especially the causes originating in nurses' skills in the awareness and detection of early signs and symptoms of conditions.

Most included studies discussed recommendations and interventions addressing the complex situation or need, resulting in possible nursing skills and competencies in e.g. pain management, nursing assessments or communication. These recommendations are highly relevant for the development of a nursing role with expanded competencies.

Due to the heterogeneity of definition of outcomes in included studies, synthesising the **reasons or conditions for the initiation of emergency medical services** proved difficult. In most studies, orthopaedic reasons were reported as one of the main reasons as in falls or injuries. Another cause reported in most studies was pneumonia, sepsis or infections in general. This coincides with results of the first research question, in which infection management was identified as being a complex care situation. In addition, causes for emergency medical services associated with dementia e.g. worsening of cognitive function or behavioural symptoms can be associated with residents with dementia innately presenting a higher degree of complexity in long-term care. The new nursing role could play

a part especially in the assessment of symptoms as well as adequately responding to those symptoms in order to reduce avoidable contacts with emergency medical services.

Contextual factors influencing the use of emergency medical services and informing the development of the new nursing role centred around the management of acute symptoms, empowerment of residents and person-centred approach to care as well as interprofessional collaboration and communication. Fields of action for nurses with expanded competencies in reducing hospital transfers could also mean improving the management of advance directives in nursing homes, regularly including family members and residents in care planning and implementing processes to improve interprofessional communication with physicians and hospitals.

Figure A5 displays nursing related results of all three literature searches synthesised regarding possible fields of actions for a new nursing role in long-term care.

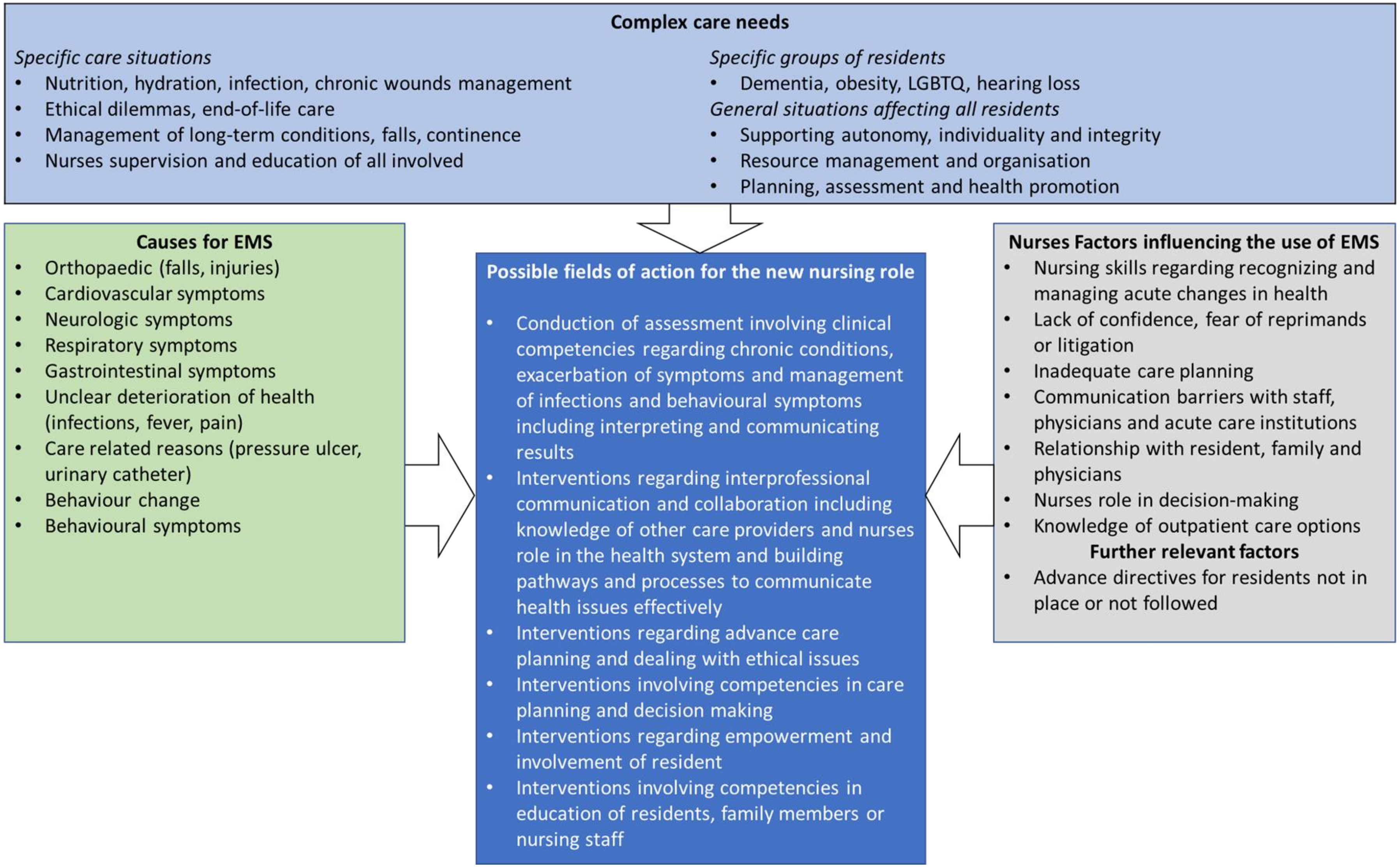

Figure A5: Synthesis of all three literature reviews regarding a new nursing role in long-term care

EMS: Emergency medical services

6. Literature

- Dwyer, R., Gabbe, B., Stoelwinder, J. U., & Lowthian, J. (2014). A systematic review of outcomes following emergency transfer to hospital for residents of aged care facilities. Age ageing, 43(6), 759–766. https://doi.org/10.1093/ageing/afu117Jeon, B., Tamiya, N., Yoshie, S., Iijima, K., & Ishizaki, T. (2018). Potentially avoidable hospitalizations, non-potentially avoidable hospitalizations and in-hospital deaths among residents of long-term care facilities. Geriatrics & gerontology international, 18(8), 1272–1279. https://doi.org/10.1111/ggi.13458
- Page, M. J., McKenzie, J. E., Bossuyt, P. M., Boutron, I., Hoffmann, T. C., Mulrow, C. D. et al. (2021). The PRISMA 2020 statement: an updated guideline for reporting systematic reviews. BMJ (Clinical research ed.), 372, n71. https://doi.org/10.1136/bmj.n71

a. Complex care situations

- Bolt, S. R., van der Steen, J. T., Schols, J. M. G. A., Zwakhalen, S. M. G., Pieters, S., & Meijers, J. M. M. (2019). Nursing staff needs in providing palliative care for people with dementia at home or in long-term care facilities: A scoping review. International journal of nursing studies, 96, 143–152. https://doi.org/10.1016/j.ijnurstu.2018.12.011
- Cadieux, M. A., Garcia, L. J., & Patrick, J. (2013). Needs of people with dementia in long-term care: a systematic review. American journal of Alzheimer's disease and other dementias, 28(8), 723–733. https://doi.org/10.1177/1533317513500840
- Cook, G., Hodgson, P., Thompson, J., Bainbridge, L., Johnson, A., & Storey, P. (2019). Hydration Interventions for older people living in residential and nursing care homes: overview of the literature. British medical bulletin, 131(1), 71–79. https://doi.org/10.1093/bmb/ldz027
- Crosbie, B., Ferguson, M., Wong, G., Walker, D. M., Vanhegan, S., & Dening, T. (2019). Giving permission to care for people with dementia in residential homes: learning from a realist synthesis of hearing-related communication. BMC medicine, 17(1), 54. https://doi.org/10.1186/s12916-019-1286-9
- Fasullo, K., McIntosh, E., Buchholz, S. W., Ruppar, T., & Ailey, S. (2022). LGBTQ Older Adults in Long-Term Care Settings: An Integrative Review to Inform Best Practices. Clinical gerontologist, 45(5), 1087–1102. https://doi.org/10.1080/07317115.2021.1947428
- Fleming, A., Bradley, C., Cullinan, S., & Byrne, S. (2015). Antibiotic prescribing in long-term care facilities: a meta-synthesis of qualitative research. Drugs & aging, 32(4), 295–303. https://doi.org/10.1007/s40266-015-0252-2
- Harris, J. A., & Castle, N. G. (2019). Obesity and Nursing Home Care in the United States: A Systematic Review. The Gerontologist, 59(3), e196–e206. https://doi.org/10.1093/geront/gnx128
- Kiljunen, O., Välimäki, T., Kankkunen, P., & Partanen, P. (2017). Competence for older people nursing in care and nursing homes: An integrative review. International journal of older people nursing, 12(3), 10.1111/opn.12146. https://doi.org/10.1111/opn.12146
- Means T. (2016). Improving quality of care and reducing unnecessary hospital admissions: a literature review. British journal of community nursing, 21(6), 284–291. https://doi.org/10.12968/bjcn.2016.21.6.284
- Watkins, R., Goodwin, V. A., Abbott, R. A., Backhouse, A., Moore, D., & Tarrant, M. (2017). Attitudes, perceptions and experiences of mealtimes among residents and staff in care homes for older adults: A systematic review of the qualitative literature. Geriatric nursing (New York, N.Y.), 38(4), 325–333. https://doi.org/10.1016/j.gerinurse.2016.12.002

b. Causes for emergency services

- Alrawi, Y. A., Parker, R. A., Harvey, R. C., Sultanzadeh, S. J., Patel, J., Mallinson, R., Potter, J. F., Trepte, N. J., & Myint, P. K. (2013). Predictors of early mortality among hospitalized nursing home residents. QJM : monthly journal of the Association of Physicians, 106(1), 51–57. https://doi.org/10.1093/qjmed/hcs188
- Amador, S., Goodman, C., King, D., Machen, I., Elmore, N., Mathie, E., & Iliffe, S. (2014). Emergency ambulance service involvement with residential care homes in the support of older people with dementia: an observational study. BMC geriatrics, 14, 95. https://doi.org/10.1186/1471-2318-14-95
- Axon, R. N., Gebregziabher, M., Craig, J., Zhang, J., Mauldin, P., & Moran, W. P. (2015). Frequency and costs of hospital transfers for ambulatory care-sensitive conditions. The American journal of managed care, 21(1), 51–59.
- Ayaz, S. I., Haque, N., Pearson, C., Medado, P., Robinson, D., Wahl, R., Zervos, M., & O'Neil, B. J. (2014). Nursing home-acquired pneumonia: course and management in the emergency department. International journal of emergency medicine, 7, 19. https://doi.org/10.1186/1865-1380-7-19
- Björck, M., & Wijk, H. (2018). Is hospitalisation necessary? A survey of frail older persons with cognitive impairment transferred from nursing homes to the emergency department. Scandinavian journal of caring sciences, 32(3), 1138–1147. https://doi.org/10.1111/scs.12559
- Briggs, R., Coughlan, T., Collins, R., O'Neill, D., & Kennelly, S. P. (2013). Nursing home residents attending the emergency department: clinical characteristics and outcomes. QJM: monthly journal of the Association of Physicians, 106(9), 803–808. https://doi.org/10.1093/qjmed/hct136
- Brownstein, H., Hayes, B., Simadri, A., Tacey, M., & Holbeach, E. (2021). Care to the end: a retrospective observational study of aged care facility residents transferred to hospital in the last day of life. Internal medicine journal, 51(1), 27–32. https://doi.org/10.1111/imj.15084
- Burke, R. E., Rooks, S. P., Levy, C., Schwartz, R., & Ginde, A. A. (2015). Identifying Potentially Preventable Emergency Department Visits by Nursing Home Residents in the United States. Journal of the American Medical Directors Association, 16(5), 395–399. https://doi.org/10.1016/j.jamda.2015.01.076
- Carron, P. N., Mabire, C., Yersin, B., & Büla, C. (2017). Nursing home residents at the Emergency Department: a 6-year retrospective analysis in a Swiss academic hospital. Internal and emergency medicine, 12(2), 229–237. https://doi.org/10.1007/s11739-016-1459-x
- Cummings, G. G., McLane, P., Reid, R. C., Tate, K., Cooper, S. L., Rowe, B. H., Estabrooks, C. A., Cummings, G. E., Abel, S. L., Lee, J. S., Robinson, C. A., & Wagg, A. (2020). Fractured Care: A Window Into Emergency Transitions in Care for LTC Residents With Complex Health Needs. Journal of aging and health, 32(3-4), 119–133. https://doi.org/10.1177/0898264318808908
- Dubucs, X., de Souto Barreto, P., Laffon de Mazieres, C., Lauque, D., Azema, O., Charpentier, S., & Rolland, Y. (2019). The Temporal Trend in the Transfer of Older Adults to the Emergency Department for Traumatic Injuries: A Retrospective Analysis According to Their Place of Residence. Journal of the American Medical Directors Association, 20(11), 1462–1466. https://doi.org/10.1016/j.jamda.2019.07.013
- Fan, C. W., Keating, T., Brazil, E., Power, D., & Duggan, J. (2016). Impact of season, weekends and bank holidays on emergency department transfers of nursing home residents. Irish journal of medical science, 185(3), 655–661. https://doi.org/10.1007/s11845-015-1332-3

- Fassmer, A. M., Pulst, A., Schmiemann, G., & Hoffmann, F. (2020). Sex-Specific Differences in Hospital Transfers of Nursing Home Residents: Results from the HOspitalizations and eMERgency Department Visits of Nursing Home Residents (HOMERN) Project. International journal of environmental research and public health, 17(11), 3915. https://doi.org/10.3390/ijerph17113915
- Givens, J. L., Selby, K., Goldfeld, K. S., & Mitchell, S. L. (2012). Hospital transfers of nursing home residents with advanced dementia. Journal of the American Geriatrics Society, 60(5), 905–909. https://doi.org/10.1111/j.1532-5415.2012.03919.x
- Griffey, R. T., Schneider, R. M., Adler, L., & Todorov, A. (2021). Post-Acute and Long-Term Care Patients Account for a Disproportionately High Number of Adverse Events in the Emergency Department. Journal of the American Medical Directors Association, 22(4), 907–912.e1. https://doi.org/10.1016/j.jamda.2020.06.043
- Grimm, F., Hodgson, K., Brine, R., & Deeny, S. R. (2021). Hospital admissions from care homes in England during the COVID-19 pandemic: a retrospective, cross-sectional analysis using linked administrative data. International journal of population data science, 5(4), 1663. https://doi.org/10.23889/ijpds.v5i4.1663
- Guion, V., de Souto Barreto, P., & Rolland, Y. (2021). Trajectories of Symptoms in Nursing Home Residents after a Transfer to the Emergency Department. The journal of nutrition, health & aging, 25(3), 318–324. https://doi.org/10.1007/s12603-020-1476-3
- Guion, V., De Souto Barreto, P., & Rolland, Y. (2021). Nursing Home Residents' Functional Trajectories and Mortality After a Transfer to the Emergency Department. Journal of the American Medical Directors Association, 22(2), 393–398.e3. https://doi.org/10.1016/j.jamda.2020.05.033
- Harrison, J. M., Agarwal, M., Stone, P. W., Gracner, T., Sorbero, M., & Dick, A. W. (2021). Does Integration of Palliative Care and Infection Management Reduce Hospital Transfers among Nursing Home Residents?. Journal of palliative medicine, 24(9), 1334–1341. https://doi.org/10.1089/jpm.2020.0577
- Hathaway, E. E., Carnahan, J. L., Unroe, K. T., Stump, T. E., O'Kelly Phillips, E., Hickman, S. E., Fowler, N. R., Sachs, G. A., & Bateman, D. R. (2021). Nursing Home Transfers for Behavioral Concerns: Findings from the OPTIMISTIC Demonstration Project. Journal of the American Geriatrics Society, 69(2), 415–423. https://doi.org/10.1111/jgs.16920
- Heinold, S., Fassmer, A. M., Schmiemann, G., & Hoffmann, F. (2021). Characteristics of outpatient emergency department visits of nursing home residents: an analysis of discharge letters. Aging clinical and experimental research, 33(12), 3343–3351. https://doi.org/10.1007/s40520-021-01863-6
- Hillen, J. B., Reed, R. L., Woodman, R. J., Law, D., Hakendorf, P. H., & Fleming, B. J. (2011). Hospital admissions from residential aged care facilities to a major public hospital in South Australia (1999-2005). Australasian journal on ageing, 30(4), 202–207. https://doi.org/10.1111/j.1741-6612.2010.00479.x
- Hsiao, C. J., & Hing, E. (2014). Emergency department visits and resulting hospitalizations by elderly nursing home residents, 2001-2008. Research on aging, 36(2), 207–227. https://doi.org/10.1177/0164027512473488
- Kim, K., Lee, D. H., Yune, H. Y., Wee, J. H., Kim, D. H., Kim, E. C., Lim, J. Y., & Choi, S. P. (2019). Identifying Potentially Avoidable Emergency Department Visits of Long-Term Care Hospital Residents in Korea: A Multicenter Retrospective Cohort Study. BioMed research international, 2019, 7041607. https://doi.org/10.1155/2019/7041607

- Kirsebom, M., Hedström, M., Wadensten, B., & Pöder, U. (2014). The frequency of and reasons for acute hospital transfers of older nursing home residents. Archives of gerontology and geriatrics, 58(1), 115–120. https://doi.org/10.1016/j.archger.2013.08.002
- Laffon de Mazières, C., Romain, M., Hermabessière, S., Abellan, G., Gerard, S., Castex, A., Krams, T., Vellas, B., & Rolland, Y. (2018). An Innovative Day Hospital Dedicated to Nursing Home Resident: A Descriptive Study of 1306 Residents Referred by their Physicians. The journal of nutrition, health & aging, 22(9), 1138–1143. https://doi.org/10.1007/s12603-018-1106-5
- Manckoundia, P., Menu, D., Turcu, A., Honnart, D., Rossignol, S., Alixant, J. C., Sylvestre, F. H., Bailly, V., Dion, M., & Putot, A. (2016). Analysis of Inappropriate Admissions of Residents of Medicalized Nursing Homes to Emergency Departments: A Prospective Multicenter Study in Burgundy. Journal of the American Medical Directors Association, 17(7), 671.e1–671.e6717. https://doi.org/10.1016/j.jamda.2016.04.017
- Morphet, J., Innes, K., Griffiths, D. L., Crawford, K., & Williams, A. (2015). Resident transfers from aged care facilities to emergency departments: can they be avoided?. Emergency medicine Australasia : EMA, 27(5), 412–418. https://doi.org/10.1111/1742-6723.12433
- Nemiroff, L., Marshall, E. G., Jensen, J. L., Clarke, B., & Andrew, M. K. (2019). Adherence to "No Transfer to Hospital" Advance Directives Among Nursing Home Residents. Journal of the American Medical Directors Association, 20(11), 1373–1381. https://doi.org/10.1016/j.jamda.2019.03.034
- Pulst, A., Fassmer, A. M., & Schmiemann, G. (2021). Unplanned hospital transfers from nursing homes: who is involved in the transfer decision? Results from the HOMERN study. Aging clinical and experimental research, 33(8), 2231–2241. https://doi.org/10.1007/s40520-020-01751-5
- Rolland, Y., Mathieu, C., Tavassoli, N., Berard, E., Laffon de Mazières, C., Hermabessière, S., Houles, M. et al. (2021). Factors Associated with Potentially Inappropriate Transfer to the Emergency Department among Nursing Home Residents. Journal of the American Medical Directors Association, 22(12), 2579–2586.e7. https://doi.org/10.1016/j.jamda.2021.04.002
- Seeger, I., Luque Ramos, A., & Hoffmann, F. (2018). Ambulante Notfallversorgung von Pflegeheimbewohnern: Auswertung von GKV-Routinedaten [Outpatient emergency treatment of nursing home residents : Analysis of insurance claims data]. Zeitschrift fur Gerontologie und Geriatrie, 51(6), 650–655. https://doi.org/10.1007/s00391-017-1293-4
- Unroe, K. T., Caterino, J. M., Stump, T. E., Tu, W., Carnahan, J. L., Vest, J. R., Sachs, G. A., & Hickman, S. E. (2020). Long-Stay Nursing Facility Resident Transfers: Who Gets Admitted to the Hospital?. Journal of the American Geriatrics Society, 68(9), 2082–2089. https://doi.org/10.1111/jgs.16633
- Walker, R. W., Palmer, J., Stancliffe, J., Wood, B. H., Hand, A., & Gray, W. K. (2014). Experience of care home residents with Parkinson's disease: Reason for admission and service use. Geriatrics & gerontology international, 14(4), 947–953. https://doi.org/10.1111/ggi.12204
- Vossius, C. E., Ydstebø, A. E., Testad, I., & Lurås, H. (2013). Referrals from nursing home to hospital: reasons, appropriateness and costs. Scandinavian journal of public health, 41(4), 366–373. https://doi.org/10.1177/1403494813484398

c. Factors influencing the use of emergency medical services

- Arendts, G., Quine, S., & Howard, K. (2013). Decision to transfer to an emergency department from residential aged care: a systematic review of qualitative research. Geriatrics & gerontology international, 13(4), 825–833. https://doi.org/10.1111/ggi.12053

- Brucksch, A., Hoffmann, F., & Allers, K. (2018). Age and sex differences in emergency department visits of nursing home residents: a systematic review. BMC geriatrics, 18(1), 151. https://doi.org/10.1186/s12877-018-0848-6
- Dwyer, R., Gabbe, B., Stoelwinder, J. U., & Lowthian, J. (2014). A systematic review of outcomes following emergency transfer to hospital for residents of aged care facilities. Age and ageing, 43(6), 759–766. https://doi.org/10.1093/ageing/afu117
- Lemoyne, S. E., Herbots, H. H., De Blick, D., Remmen, R., Monsieurs, K. G., & Van Bogaert, P. (2019). Appropriateness of transferring nursing home residents to emergency departments: a systematic review. BMC geriatrics, 19(1), 17. https://doi.org/10.1186/s12877-019-1028-z
- Marincowitz, C., Preston, L., Cantrell, A., Tonkins, M., Sabir, L., & Mason, S. (2022). What influences decisions to transfer older care-home residents to the emergency department? A synthesis of qualitative reviews. Age and ageing, 51(11), afac257. https://doi.org/10.1093/ageing/afac257
- O'Neill, B., Parkinson, L., Dwyer, T., & Reid-Searl, K. (2015). Nursing home nurses' perceptions of emergency transfers from nursing homes to hospital: A review of qualitative studies using systematic methods. Geriatric nursing (New York, N.Y.), 36(6), 423–430. https://doi.org/10.1016/j.gerinurse.2015.06.001
- Pulst, A., Fassmer, A. M., & Schmiemann, G. (2019). Experiences and involvement of family members in transfer decisions from nursing home to hospital: a systematic review of qualitative research. BMC geriatrics, 19(1), 155. https://doi.org/10.1186/s12877-019-1170-7
- Trahan, L. M., Spiers, J. A., & Cummings, G. G. (2016). Decisions to Transfer Nursing Home Residents to Emergency Departments: A Scoping Review of Contributing Factors and Staff Perspectives. Journal of the American Medical Directors Association, 17(11), 994–1005. https://doi.org/10.1016/j.jamda.2016.05.012

Chapter B: Stakeholder workshops and survey

1. Aims

We conducted two workshops with stakeholders, to collect their views regarding the relevance of problem areas as well as the priority and feasibility of nursing interventions. Thus, we aimed to gain a clearer understanding of the role of nurses with expanded competencies in long-term care and of barriers and implementation strategies when implementing the role within a randomized controlled trial.

Aims of the workshops were:

1. to discuss and identify priority problems and potential competencies and fields of actions for the new model of care
2. to prioritise competencies of the new nursing role and to identify implementation strategies and potential barriers in the implementation of new nursing roles in long-term care.

2. Methods

a. Eligibility criteria and recruitment

Potential participants of the workshops were primarily experts and stakeholders who had already submitted a written cooperation agreement for participation in interviews, focus groups and expert advice as part of the application of the project. When inviting participants, the aim was to recruit at least one participant from their fields of expertise or background. The background of the study advisory board panellists and further relevant experts (n=25) are depicted in Table B15.

Table B15: Background and number of panellists invited to take part in the workshops

Background	Number of panellists
Care / Health Management, nursing home care manager	5
Palliative Medicine, general practitioners	5
Nursing sciences, research in primary care	4
Nurse, B.Sc.	3
Elderly advisory board, advocacy for nursing home residents	2
Association of providers of social services	1
Medical association	1
Ministry of social affairs	1
Health insurance company	1
Medical law attorney	1
Nursing association	1
**Total**	**25**

We recruited stakeholders via e-mail and provided information about the workshop aims and data management. Participants received 50 Euro compensation for each workshop. Participation in only one of the two workshops was possible. The aim was to recruit around 15 participants per workshop. Participants gave written consent for their contributions to be used in the current study and for further research.

We sent the invitation to complete the online survey for the rating of relevant nursing competencies to all experts and stakeholders affiliated with the project regardless of participation in the workshops.

b. Data collection

We collected no personal data during the workshops, but how many representatives of the targeted fields participated. The study team documented participants' statements in minutes. We collected data by using creative techniques like mind maps and brain storming. W conducted the workshops using WebEx video conferencing tool (WebEx by Cisco, 2021), and collected and displayed results with an online whiteboard (Miro-board, miro, 2021). Qualitative data from protocols and the survey were analysed thematically and summarized by category.

We conducted the online survey via the open source survey tool Limesurvey (Limesurvey GmbH, 2021). Invitations were sent out by E-Mail. The data was collected anonymously. Participation was voluntary. We did not seek ethical approval, since the survey was conducted as part of the roles of the Expand-Care advisory board members as well as the duties within workshop participation.

c. Description of workshop 1

The first workshop took place in the end of November 2021. The aim was to discuss priority problems in long-term care and identify relevant fields of actions for nurses with expanded competencies. The workshop lasted three and a half hours covering different aspects and interactive components (Table B16). We conducted working phases in small groups of participants with subsequent presentation and discussion of results with the whole group.

Table B16: Content of workshop 1

1.	Welcome and introduction of participants
2.	Introduction of aims of workshop in the context of intervention development
3.	**First working phase:**<br>Participants were given a short case scenario based on care situations found in the multiple case study. The groups were then asked to discuss potential current or future care problems, gaps or breaks.
4.	**Second working phase:**<br>Participants discussed all potential kinds of interventions (based on the problems/gaps/breaks identified) they would implement they could think of without considering restrictions on finance or other resources.
5.	**Third working phase:**<br>Participants discussed the previously collected changes or interventions according to relevance and feasibility.
6.	Summary, feedback and outlook on second workshop

d. Results of workshop 1

Ten Stakeholders participated in the first workshop. Background of participants is depicted in Table B17.

Table B17: Participants in workshop 1

Background	Number of participants
Care / Health Management, nursing home care manager	2
Palliative Medicine, general practitioners	2
Nursing sciences, research in primary care	1
Nurse, B.Sc.	2

Elderly advisory board, advocacy for nursing home residents	2
Nursing association	1
**Total**	**10**

The tasks or competencies depicted in Table B18 emerged from the discussion and rating process in workshop 1. There were no tasks rated as of low relevance.

Table B18: Tasks or competencies discussed in workshop 1

**High relevance & high feasibility**
• Implement and/or evaluate adequate forms for communication via fax machine<br>• Improve and evaluate fall prevention<br>• Improve management of respiratory diseases<br>• Education of nursing team in symptoms of chronic diseases<br>• Ensure a prospective care planning in case of health deterioration or change of needs<br>• Implement emergency plans for residents<br>• Improve management of on demand medication in collaboration with general practitioners<br>• Improve communication in nurses' handovers<br>• Priorities are clear in communication with other service providers<br>• Advance directives and wishes for the end of life are known to all involved in care and are at hand<br>• Ensure the skills and competency mix in facility is clear and differentiated regarding tasks<br>• Nurses act as advocates for residents in all aspects of care and communication
**High relevance but low feasibility**
• Organise and coordinate weekly visits by general practitioners<br>• Discuss use of emergency telephone numbers with general practitioners<br>• Improve competencies in case and care management similar to community health nurse<br>• Discuss and implement the use of consultations and communication processes per video or telephone<br>• Implement regular case conferences with all stakeholders involved in care as well as residents<br>• Implement and improve use of digital media in communication processes and documentation<br>• Nurse visit residents in hospital after hospitalisation in order to discuss care changes before return to facility<br>• Implement person of trust for residents in facilities<br>• Implement processes to integrate relatives and social network into care processes<br>• Limit the number of general practitioners responsible for residents in facility<br>• Increase time spent with residents, decrease time spent documenting<br>• Discuss implementation of an emergency number with emergency medical services in case of acute exacerbation of symptoms out of hours<br>• Nurse visits residents living situation, needs and social network before moving into facility to ensure nurses are prepared for new residents<br>• Nurses have the competencies to use electrocardiograms in long-term care facilities

e. Mini-survey

Following the first workshop, we synthesised relevant competencies and tasks and expanded them with results of the literature reviews (chapter A), and the multiple case study (Pohontsch et al. [under preparation]). We further reviewed the final list and complemented it with relevant competencies for registered nurses in expanded roles as identified in a swiss Delphi study (Basinska et al., 2020). We merged all tasks and competencies subsequently through repeated team discussions into four areas of competencies, in which various fields of action were categorized and described (Table B19).

Table B19: Areas of competencies and fields of action

Establishment and maintenance of a person-centered care network	Management of chronic and geriatric diseases	Empowerment and communication with residents	Organisation / Facility
• Maintenance of a medical care network<br>• Participation of relatives<br>• Discharge and transition management with acute care providers<br>• Management of therapeutic measures<br>• Timely prescription of services	• Symptom control in chronic diseases<br>• Dealing with acute symptoms<br>• Nursing support for medical care<br>• Evaluation of the care situation<br>• Management of medical aids<br>• Health promotion and prevention	• Communication with (relatives and) residents<br>• Advocacy<br>• Prioritisation of care issues with resident preferences<br>• Psychosocial inclusion<br>• Advance Care Planning	• Quality of care based on current scientific standards<br>• Care level management<br>• Dealing with rules and standards<br>• Internal distribution of tasks in the care process: organising skill mix<br>• Designing learning processes<br>• Internal communication/ documentation<br>• Values and norms of the facility<br>• Social network as a resource

The research team devised 55 tasks within the fields of action. We asked participants to rate clinical and practical tasks for nurses with expanded competencies in long-term care facilities regarding relevance and feasibility. We used a rating scale based on "The RAND/UCL Appropriateness Method User's Manual" (Fitch et al., 2001). For each task, participants could rate priority on a scale of 1 to 9 in which numbers from 1 and 3 indicated a "low priority", numbers from 4 and 6 a "general importance, but not an essential priority" and numbers from 7 to 9 the "highest priority and crucial importance". Additionally, we asked participants to express whether they felt that specific tasks were missing, not appropriate or feasible in optional text questions. The complete survey questionnaire is provided as additional information (at the end of this chapter). The original German version has been translated into English by the research team for the purpose of reporting in this manuscript.

We calculated Median and interquartile range using the SPSS software for statistical analysis (IBM, 2020) for each task and competency rated in the survey, and the percentage of ratings below a value of 7. We categorized tasks and competencies as crucially important with a median between 7 and 9, important, but not essential with a median between 4 and 7 and unimportant for the Expand-Care study if the median was below 4.

Participants received invitation to complete the survey between December 2021 and beginning of January 2022. We discussed results in preparation of the second workshop with particular focus on diverging (interquartile range over 3) or surprising results.

f. Results of survey data

13 panellists (52 %) participated in the online survey. Not all participants completed the survey, missing data was not imputed. Due to technical difficulties, two items missed in the survey and participants in workshop 2 following the survey rated these during the workshop (*items marked in italics*). The median, interquartile range and percentage of ratings below 7 of each task are displayed in Table B20, Table B21, Table B22 and Table B23.

Table B20: Results for "Development and maintenance of a person-centered care network" (n=13)

Task	Median (scale 1 to 9)	Interquartile range	Percentage of ratings below 7 (%)
**Rating: highest priority**			
Agree on a checklist for hospital stays with all service providers, for example:<br>1) what does a resident need for the hospital stay<br>2) are all prerequisites for the planned procedure met (e.g. medication or medication change)<br>3) what does the resident need for relocation to the NH	8	1.5	15
Ensure that GP and specialists visits to the nursing home are accompanied by a nurse who has previously collected relevant information and concerns.	8	2	23
Create and maintain a resident-related quick overview of the most important aspects on one sheet: e.g. living will, hospital admission desired/rejected, emergency contact (accessible in the resident's room).	8	5.5	46
Carry out case conferences by telephone / video with residents or patients and Expand-Care experts, GPs and specialists.	7	1	15
Initiate, plan and implement multi-professional cooperation with service providers (e.g. with physiotherapists).	7	2	46
Visit residents who are hospitalized prior to discharge and speak to the nursing team to prepare for important adjustments to health care at the NH. Alternatively, arrange structured telephone consultation.	7	2.5	46
Establish cooperation with GPs to delegate medical activities: e.g. prescription of medication	7	3	42
Establish cooperation with GPs to delegate medical activities: e.g. prescription of therapeutic measures	7	3	42
If necessary, conduct case discussions or additional visits with residents, relatives, nursing staff and GPs and specialists and, if necessary, other stakeholders involved.	7	3	38
**Rating: important, but not crucially essential**			
Make an agreement with clinics that prescriptions for follow-up medication (including oxygen) can be prepared or requested as part of the discharge management.	6	2	69
Agreement with GPs and specialists for which topics ward rounds and specialist communication should be used for and create communication guidelines for ward rounds.	6	2.5	69

Task	Median (scale 1 to 9)	Interquartile range	Percentage of ratings below 7 (%)
Structured communication with residents and relatives about the distribution of tasks in medical care: defining responsibilities and the flow of information.	6	3	54
Establish cooperation with GPs to delegate medical activities: e.g. prescribing patient transport	6	3	46
Actively obtain the assessment of relatives and surrogates in a structured manner and integrate them into the documentation as part of the care planning and the care process	6	3.5	54
Arrangements with residents or carers so that staff members of the NH are authorized to obtain information from the clinic or hospital.	6	4.5	62
Agreement with GPs and specialists that residents can receive appointments at a time appropriate to their needs.	5	3	69
Agreement with GPs and specialists on structured communication, e.g. developing pre-structured fax templates, supporting assessment of urgency levels.	5	3	54
Develop a central documentation form within the NH for use by all medical disciplines involved: What changes (e.g. new diagnosis, medication) are made by whom.	5	3	54

GP: General practitioner; NH: Nursing home.

Table B21: Results of "Management of chronic and geriatric diseases" (n=12)

Task	Median (scale 1 to 9)	Interquartile range	Percentage of ratings below 7 (%)
**Rating: highest importance**			
Implementation and interpretation of geriatric assessments, e.g.: mobility, falls, cognition, delirium, nutritional status, pain assessment in the case of cognitive impairments, skin condition, continence, wound assessment, if necessary	8	1.75	17
Comprehensive pain management for chronic pain: identification of needs, monitoring and initiation of pain therapy	8	1.75	8
Establishment of decision-making paths for key symptoms: Assess clinical relevance and need for action and initiate appropriate measures (e.g. in the case of abdominal pain, dyspnoea, fever, oedema, itching ...)	8	1.75	17
Independent treatment of chronic wounds and wound care in cooperation with certified wound experts.	8	1.75	17
Promote safe medication administration: e.g. training of employees, development of information material, evaluation of the handling of medication (e.g. special administration).	8	2.5	25
Evaluate complex care situations through regular care visits while taking the care and health related history of residents into account (care anamnesis, care report, re-assessment...) and initiate appropriate measures.	8	2.75	17
Targeted observation of residents when there is a change in medication.	7.5	1.75	25
Lead multi-professional case reviews, in which the development processes of undesired or adverse events are reflected upon and preventive measures are initiated.	7.5	3	33
Initiate medication review based on defined events and clinical assessment.	7	0	17

Task	Median (scale 1 to 9)	Interquartile range	Percentage of ratings below 7 (%)
Identify and plan nursing interventions to support individual health behaviour and support their implementation, for example through instruction, training, education, information and advice.	7	1	42
Derive recommendations and measures from the nursing visits beyond the nursing care service (risk factor-related measures of primary, secondary and tertiary prevention: e.g. prophylaxis).	7	1	42
Hold regular meetings with other care providers (GPs, therapists) about the status and further progress.	7	1.75	42
Check the need for mobility and communication aids and initiate supply: Use of the catalogue of aids, formulation of recommendations for aids, initiating provision (e.g. application for cost absorption, purchase).	7	2	42
Establishment of standards specific to the NH (e.g. checklist or adaptation of existing instruments) for symptom control of chronic diseases.	7.5	2.75	42
Design and distribute information material and training courses on common side effects of medication for all nursing staff (e.g. posters on the subject of dizziness as a risk factor for falls).	7	2.75	33
Intravenous administration of medication (e.g. for fluid substitution)	7	2.75	42
Determine / formulate individual health values and realistic goals in a structured manner in dialogue with the residents.	7	2	42
**Rating: important, but not crucially essential**			
Apply nursing strategies to change health conditions, for example by promoting supportive social networks or health promotion measures related to the setting of the NH and for all people living and working there.	6	1.75	67

GP: General practitioner; NH: Nursing home.

Table B22: Results of "Empowerment and communication with residents" (n=12)

Task	Median (scale 1 to 9)	Interquartile range	Percentage of ratings below 7 (%)
**Rating: highest importance**			
Offer structured advice on advance care planning for residents and their relatives and document the results of the advice (contents, decisions, powers of attorney).	9	2	8
Regular nursing visits and case discussions involving residents, relatives or persons of trust. Clarify conflicting assessments of care problems with resident preferences.	8	3	33
Structured observation of psychosocial well-being after moving in. Individual support for integration into the NH and everyday life.	8	2	17
Inform the residents about nursing assessments as well as the benefits and risks of interventions in order to enable them to make an informed decision (= informed consent).	7.5	1.75	25
Use person-centred communication in nursing visits or case discussions: Encourage residents to communicate. Design communication as a trialogue (equal cooperation between residents, relatives and professionals).	7	2.75	42

Task	Median (scale 1 to 9)	Interquartile range	Percentage of ratings below 7 (%)
Transparently present responsibilities in NH area:<br>1. Legible nameplates, current staff wall, laminated overviews with photos and names of employees per residential unit in the resident's room,<br>2. Information letter: Overview of the "most important" contact person for relatives, including responsibilities.	7	5.25	42
**Rating: important, but not crucially essential**			
Ensure that the presumed or documented will to initiate life-prolonging measures is known in the medical and nursing team and promote its consideration in decisions.	*Task rated during workshop 2 (n=7)*		
	6	-	57

GP: General practitioner; NH: Nursing home.

Table B23: Results "Organisation and nursing home facility" (n=11)

Task	Median (scale 1 to 9)	Interquartile range	Percentage of ratings below 7 (%)
**Rating: highest importance**			
Establish training and learning opportunities on evidence-based care, expert standards and nursing guidelines in NH	9	2	18
Development of decision-making questions/aids for common phenomena that result in hospital admission (fall, dehydration, change of behavior).	9	2	9
Structure handover of care to ensure priority information transfer (e.g. "SBAR" concept)	8	2	9
Work with expert standards and nursing care guidelines:<br>1. Adapt and prepare to the context of the NH<br>2. Evaluate application	8	3	27
Management of the reflection on decision-making with the nursing team within:<br>• Structured case reviews<br>• Collegial advice<br>• Staff supervision	8	3	27
Reflect on nursing practice, identify problem areas and formulate research questions. Search for evidence, evaluate and prepare it for practice, and communicate it within the NH	8	3	27
Regular evaluation of the use of standards in the NH. Regular adjustment of standards based on current evidence.	*Task rated in workshop 2 (n=7)*		
	8		29
Working with social resources: Analysis of the social network, communication with the social network, maintaining and documenting contacts.	7	2	45
Description of tasks, responsibilities and information distribution:<br>• Within job descriptions<br>• Through the appointment of the persons responsible for the support in the respective process.<br>Application and implementation:<br>• Concepts for training of new nursing staff<br>• Structured reflection and communication about teamwork with the team	7	3	45

Task	Median (scale 1 to 9)	Interquartile range	Percentage of ratings below 7 (%)
Reflection on values that guide nursing and development of a "good care" model together with the NH team.	7	3	45
Determine changes in the care needs of residents and initiate the need for a review of the degree of provided and refinanced care (if necessary, cooperation with relatives or carers).	7	3	27
**Rating: important, but not crucially essential**			
Communicate educational and networking offers from professional nurse associations in the NH.	6	3	55

GP: General practitioner; NH: Nursing home.

The number of tasks rated within the highest importance (median 7 through 9) overall per area of competency are depicted in Table B24.

Table B24: Number of tasks rated with the highest importance per area of competency

Area of competency	Number of tasks	Tasks rated with highest priority	
	n	n	%
Development and maintenance of a person-centered care network	18	8	44
Management of chronic and geriatric diseases	18	17	94
Empowerment and communication with residents	7	6	86
Organisation and nursing home facility	12	8	67
**Total**	**55**	**39**	**71**

Five participants answered the optional text input questions. Two major themes emerged in the analysis of the text (Table B25).

Table B25: Themes emerging from panellists' comments

Themes	Description
Some tasks don't call for the need for expanded competency	• Some tasks were not rated as essential for nurses with expanded competencies, since nurses with standard qualification are already able to complete these tasks or the tasks were seen as responsibilities of nursing home management.<br>• Nurses with expanded competencies could initiate and oversee certain tasks, without doing them themselves
Closeness to direct care of resident important	• Expanded Nurse should primarily be involved in tasks close to the residents (e.g. clinical assessments, coaching and guidance of residents), less so in tasks completed in offices

The areas of competency with the highest rated tasks were the management of chronic and geriatric diseases and empowerment and communication with residents. Tasks within these two areas are primarily fulfilled close to residents (e.g. carrying out clinical assessments, coaching, enabling informed consent) whereas tasks within the two other areas mostly target interprofessional cooperation or processes in nursing homes (e.g. agreements with GPs and specialists on structured communication, organize skill mix and responsibilities, establish in-house training). The results of the survey were discussed with panellists in workshop 2.

g. Description of workshop 2

The second workshop took place in the beginning of January 2022. The aim was to identify implementation barriers and strategies. The workshop lasted four hours covering different aspects and interactive components (Table B26). Working phases were conducted in small groups of participants with subsequent presentation and discussions of results with the whole group.

Table B26: Contents of workshop 2

1.	Welcome and introduction of participants
2.	Presentation of results of workshop 1 and online survey
3.	**First working phase:**<br>Discussion of the rating of tasks and competencies and diverging outcomes of the online survey
4.	Presentation and explanation of CFIR tool
5.	**Second working phase:**<br>Rating of potential barriers within the CFIR framework when implementing a new nursing role in long-term care facilities.
6.	Discussion of key barriers, facilitating factors and strategies for implementation
7.	Summary and feedback

CFIR: Consolidated Framework for Implementation Research (Damschroder et al., 2009)

We used the CFIR framework (Consolidated Framework for Implementation Research, Damschroder et al., 2009; Regauer et al., 2021) to categorize and discuss implementation barriers in the workshop. The framework contains five domains, in which different barriers hindering implementation processes are described in 37 different constructs (Table B27).

Table B27: Domains and constructs of CFIR framework (Damschroeder et al., 2009)

	Domain	Constructs	
**1.**	Intervention characteristics	• Intervention Source<br>• Evidence strength & quality<br>• Relative advantage<br>• Adaptability	• Trialability<br>• Complexity<br>• Design quality & packaging<br>• Cost
**2.**	Outer setting	• Patient needs and resources<br>• Cosmopolitanism<br>• Peer pressure	• External policy & incentives
**3.**	Inner setting	• Implementation Climate<br>o Tension for change<br>o Compatibility<br>o Relative priority<br>o Organizational incentives & rewards<br>o Goals & feedback<br>o Learning climate	• Structural Characteristics<br>• Networks & Communications<br>• Culture<br>• Readiness for Implementation<br>o Leadership Engagement<br>o Available Resources<br>o Access to knowledge & information
**4.**	Characteristics of individuals	• Knowledge & beliefs about the intervention<br>• Self-efficacy<br>• Individual stage of change	• Individual identification with organization<br>• Other personal attributes
**5.**	Process	• Engaging	• Planning

		○ Opinion leaders<br>○ Formally appointed internal implementation leaders<br>○ Champions<br>○ External change agents	• Executing<br>• Reflecting & evaluating

In the second working phase of the workshop, participants rated the relevancy of barriers within each domain. We first presented and explained the framework. We used the survey tool integrated in WebEx by Cisco for the rating procedure. Participants anonymously chose constructs with the highest relevance to the implementation of a new nursing role in long-term care per domain. Each participant was allowed to award half as many points as there were constructs in a category. We synthesised and discussed results after a break.

h. Results of workshop day 2

Eight stakeholders participated in the second workshop. The professional background of participants is depicted in *Table B28*.

Table B28: Participants at workshop 2

**Background**	**Number of participants**
Care / Health Management, nursing home manager	1
Palliative Medicine, general practitioners	1
Nursing sciences, research in primary care	2
Medical law	1
Elderly advisory board, advocacy for nursing home residents	2
Nursing association	1
**Total**	**8**

In the first part, we discussed results of the online survey, in particular items with a diverging rating (interquartile range over 3). In the second part, participants rated the relevance of implementation barriers as described in the framework. The constructs rated as important by over 66 % of participants are depicted in Table B29.

Table B29: CFIR constructs rated as important by participants

	**Domain**	**Construct**	**Proportion of participants rating this important (n=8)**
**1.**	Innovation characteristics	Relative advantage<br>Adaptability	75 %<br>88 %
**2.**	Outer setting	Peer pressure<br>External policy and incentives	75 %<br>75 %
**3.**	Inner setting	Leadership engagement<br>Available resources	75 %<br>75 %

**4.**	Characteristics of individuals	Individual Stage of Change	88 %
**5.**	Process	Opinion leaders<br>Key stakeholders	88 %<br>88 %

Participants perceived a barrier in nurses and other stakeholders not seeing an advantage in a new nursing role in long-term care compared to usual care as well as the new role not being adaptable to individual facilities when tested in a trial. Participants perceived a lack of pressure to implement an intervention or innovation in order to ensure quality care and to be an attractive facility both for residents as well as employees as external barriers. Also, they perceived current policies, financial regulations and trans-sectoral care processes as barriers.

Further important barriers were both managerial as well as nursing commitment and enthusiasm in implementing and compliance in the intervention. Associated with this, the individuals who have formal or informal influence on the attitudes and beliefs of their colleagues with respect to implementing the intervention might pose a barrier.

Lastly, participants rated the lack of resources in particular nursing staff and time as an important barrier.

3. Literature

- Damschroder, L. J., Aron, D. C., Keith, R. E., Kirsh, S. R., Alexander, J. A., & Lowery, J. C. (2009). Fostering implementation of health services research findings into practice: a consolidated framework for advancing implementation science. Implementation science : IS, 4, 50. https://doi.org/10.1186/1748-5908-4-50
- Fitch, K., Bernstein, S. J., Aguilar, M. S., Burnand, B., LaCalle, J. R. & Lazaro, P. et al. (2001). The RAND/UCLA Appropriateness Method User's Manual. RAND corporation 2001. Verfügbar unter: http://www.rand.org/pubs/monograph_reports/MR1269.
- IBM Corp. (2020). IBM SPSS Statistics for Windows Version 27.0. Armonk, NY: IBM Corp. https://www.ibm.com/analytics/spss-statistics-software
- Limesurvey GmbH (2021). LimeSurvey: An Open Source survey tool. LimeSurvey GmbH, Hamburg, Germany. http://www.limesurvey.org
- Miro (2021) [Software]. Miro. https://miro.com/de/
- Regauer, V., Seckler, E., Campbell, C., Phillips, A., Rotter, T., Bauer, P. & Müller, M. (2021). German translation and pre-testing of Consolidated Framework for Implementation Research (CFIR) and Expert Recommendations for Implementing Change (ERIC). Implement Sci Commun. 2021 Oct 19;2(1):120. https://doi.org/10.1186/s43058-021-00222-w
- WebEx by Cisco (42.1.0.21190) [Software]. (2021). Cisco. https://www.webex.com/de/index.html
- Pohontsch et al. Fields of action for nurses with expanded competencies in long-term care: a multiple case study with root-cause analysis. (under preparation)

Additional material to chapter B: Translated mini-survey questionnaire

Translation for the purpose of reporting in this manuscript

Presentation of competency areas and tasks as role of the Expand-Care nursing professional

Dear Sir or Madam,

We would like to thank you for your diverse support as a member of the advisory board, participants in the multiple case study and in the first workshop! We have now combined results from all previous parts of the study into a structure of competency areas and tasks for the role of the Expand-Care nursing professional. Potential tasks that could be assigned to the role will be prioritised and specified in a set of mandatory and additional tasks in the next step.

With this survey, we are asking you for your assessment of which of the tasks listed below you consider most important for implementation in the context of the Expand-Care pilot study.

The tasks are presented to you according to five competency areas. You can rate each task on a scale of 1 to 9. You also have the opportunity to make a free comment. Your assessment will be the basis for the discussion of the final role profile in the next workshop on January 10, 2022.

We therefore ask you to take part in the survey by January 7th, 2022. We expect it to take around 20 minutes, but this can of course vary from person to person.

The Expand-Care team would like to say a big thank you and wishes you a wonderful and relaxing Christmas!

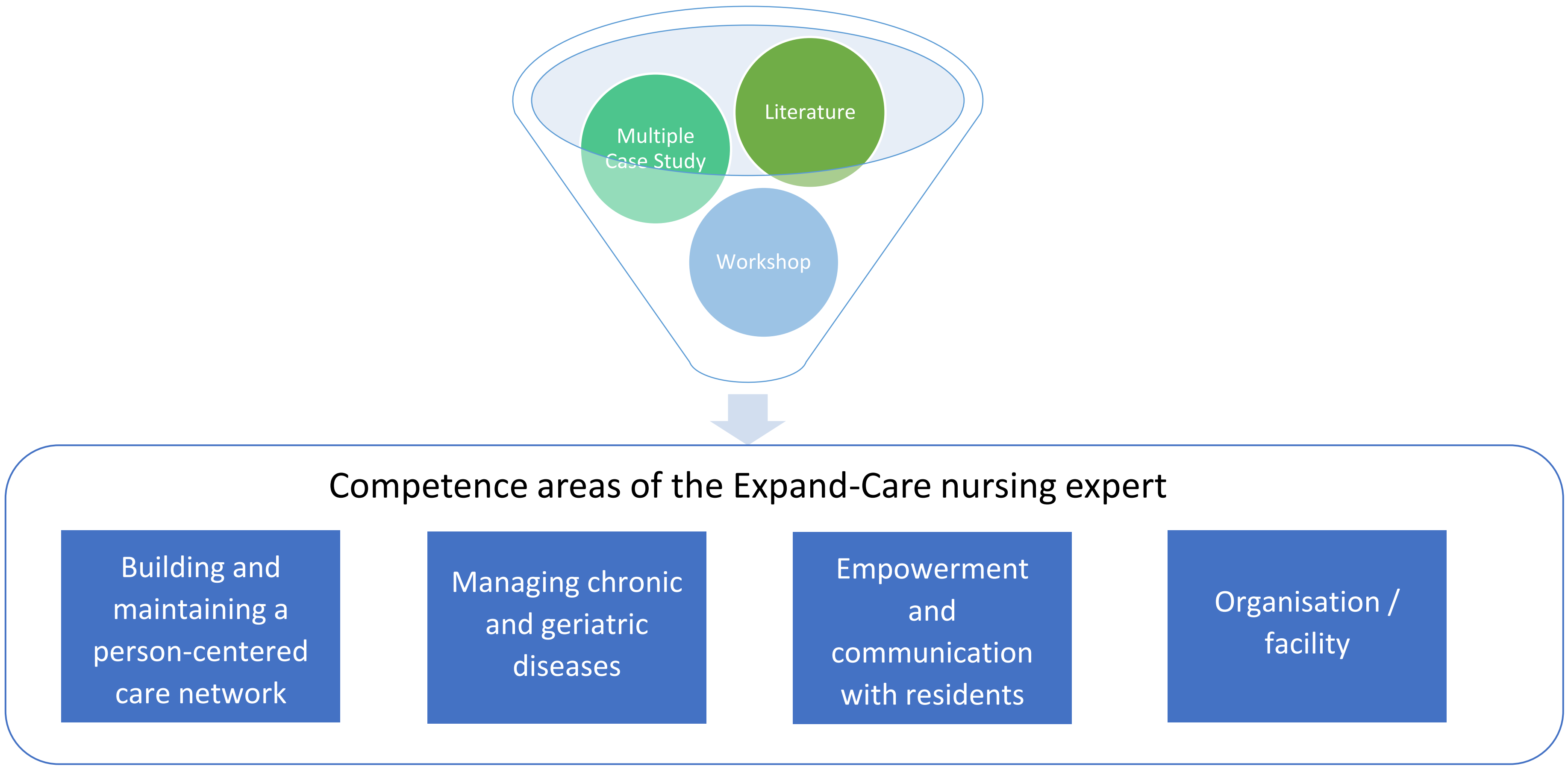
Multiple Case Study
Literature
Workshop
Competence areas of the Expand-Care nursing expert
Building and maintaining a person-centered care network
Managing chronic and geriatric diseases
Empowerment and communication with residents
Organisation / facility

This survey contains 8 questions.

Building and maintaining a person-centered care network

This competency area includes the fields of action of maintaining a medical care network, participation of relatives, discharge and transfer management with inpatient acute care providers, medicinal product management and timely prescription of services and devices.

The tasks listed below are assigned to these fields of action. Please rate which of these tasks you consider most important for implementation as part of the Expand-Care pilot study, i.e. which tasks the nursing professional with expanded competencies should take on.

Note on the meaning of the scale:

Levels 1 to 3 correspond to low relevance / priority

Levels 4 to 6 mean that these tasks are important but not of crucial importance.

Levels 7 to 9 correspond to the highest priority and crucial importance.

Please select the answer for each point:

#		1	2	3	4	5	6	7	8	9
1.1	Agreement with GPs and specialists that residents can receive appointments at a time appropriate to their needs.	○	○	○	○	○	○	○	○	○
1.2	Agreement with GPs and specialists on structured communication, e.g. developing pre-structured fax templates, enabling assessment of urgency levels.	○	○	○	○	○	○	○	○	○
1.3	Carry out case conferences by telephone / video with residents or patients and Expand-Care experts, GPs and specialists.	○	○	○	○	○	○	○	○	○
1.4	Develop a central documentation system within the NH for use by all medical disciplines involved: What changes (e.g. new diagnosis, medication) are made by whom.	○	○	○	○	○	○	○	○	○
1.5	Agreement with GPs and specialists on what ward rounds and specialist communication should be used for and create communication guidelines for ward rounds.	○	○	○	○	○	○	○	○	○

#		1	2	3	4	5	6	7	8	9
1.6	Ensure that GP and specialists' visits to the nursing home are accompanied by a nurse who has previously collected relevant information and concerns about residents.	◯	◯	◯	◯	◯	◯	◯	◯	◯
1.7	Create and maintain a resident-related quick overview of the most important aspects on one sheet: e.g. living will, hospital admission desired/rejected, emergency contact (accessible in the resident's room).	◯	◯	◯	◯	◯	◯	◯	◯	◯
2.1	Actively obtain the assessment of residents' well-being by relatives and surrogates in a structured manner and integrate them into the documentation as part of the care planning and the care process.	◯	◯	◯	◯	◯	◯	◯	◯	◯
2.2	If necessary, conduct case discussions or additional visits with residents, relatives, nursing staff and GPs and specialists and, if necessary, other stakeholders involved.	◯	◯	◯	◯	◯	◯	◯	◯	◯
2.3	Structured communication with residents and relatives about the distribution of tasks in medical care: defining responsibilities and the flow of information.	◯	◯	◯	◯	◯	◯	◯	◯	◯
3.1	Make an agreement with clinics that prescriptions for follow-up medication (including oxygen) can be prepared or requested as part of the discharge management.	◯	◯	◯	◯	◯	◯	◯	◯	◯
3.2	Arrangements with residents or carers so that the staff members of the NH are authorized to obtain information from the clinic or hospital.	◯	◯	◯	◯	◯	◯	◯	◯	◯
3.3	Visit residents who are hospitalized prior to discharge and speak to the nursing team to prepare for important adjustments to health care at the NH. Alternatively, arrange structured telephone consultation.	◯	◯	◯	◯	◯	◯	◯	◯	◯
3.4	Agree on a checklist for hospital stays with all service providers, for example:<br>1) what does a resident need for the hospital stay<br>2) are all prerequisites for the planned procedure met (e.g. medication or medication change)<br>3) what does the resident need for relocation to the NH	◯	◯	◯	◯	◯	◯	◯	◯	◯

#		1	2	3	4	5	6	7	8	9
4.1	Initiate, plan and implement multi-professional cooperation with service providers (e.g. with physiotherapists).	○	○	○	○	○	○	○	○	○
5.1	Establish cooperation with GPs to delegate medical activities: e.g. prescription of patient transport	○	○	○	○	○	○	○	○	○
5.2	Establish cooperation with GPs to delegate medical activities: e.g. prescription of medication	○	○	○	○	○	○	○	○	○
5.3	Establish cooperation with GPs to delegate medical activities: e.g. prescription of therapeutic measures	○	○	○	○	○	○	○	○	○

What other comments would you like to add to this competency area?

Are there any activities missing that you think could be of high importance?

Please enter your answer here:

Managing chronic and geriatric diseases

This area of expertise includes the fields of symptom control in chronic diseases, dealing with newly emerging symptoms, nursing support for medical care, evaluation of the care situation, health services management, and health promotion and prevention.

Note on the meaning of the scale:

Levels 1 to 3 correspond to low relevance / priority

Levels 4 to 6 mean that these tasks are important but not of crucial importance.

Levels 7 to 9 correspond to the highest priority and crucial importance.

Please select the answer for each point:

#		1	2	3	4	5	6	7	8	9
1.1	Establishment of standards specific to the NH (e.g. checklist or adaptation of existing instruments) for symptom control of chronic diseases.	◯	◯	◯	◯	◯	◯	◯	◯	◯
1.2	Implementation and interpretation of geriatric assessments, e.g.: mobility, falls, cognition, delirium, nutritional status, pain assessment in the case of cognitive impairments, skin condition, continence, wound assessment if necessary	◯	◯	◯	◯	◯	◯	◯	◯	◯
1.3	Comprehensive pain management for chronic pain: identification of needs, monitoring and initiation of pain therapy	◯	◯	◯	◯	◯	◯	◯	◯	◯
2.1	Establishment of decision-making paths for key symptoms: Assess clinical relevance and need for action, and initiate appropriate measures (e.g. in the case of abdominal pain, dyspnoea, fever, oedema, itching ...)	◯	◯	◯	◯	◯	◯	◯	◯	◯
3.1	Design and distribute information material and training courses on common side effects of medication for all nursing staff (e.g. posters on the subject of dizziness as a risk factor for falls).	◯	◯	◯	◯	◯	◯	◯	◯	◯
3.2	Targeted observation of residents when there is a change in medication.	◯	◯	◯	◯	◯	◯	◯	◯	◯

#		1	2	3	4	5	6	7	8	9
3.3	Promote safe medication administration: e.g. training of staff, development of information material, evaluation of the handling of medication (e.g. special administration).	○	○	○	○	○	○	○	○	○
3.4	Intravenous administration of medication (e.g. for fluid substitution)	○	○	○	○	○	○	○	○	○
3.5	Initiate medication review based on defined events and clinical assessment.	○	○	○	○	○	○	○	○	○
3.6	Independent treatment of chronic wounds and wound care in cooperation with certified wound experts.	○	○	○	○	○	○	○	○	○
4.1	Evaluate complex care situations through regular care visits while taking the care and health related history of residents into account (care anamnesis, care report, re-assessment...) and initiate appropriate measures.	○	○	○	○	○	○	○	○	○
4.2	Hold regular meetings with other care providers (GPs, therapists) about the status and further progress.	○	○	○	○	○	○	○	○	○
5.1	Check the need for mobility and communication aids and initiate supply: Use of the catalogue of aids, formulation of recommendations for aids, initiating provision (e.g. application for cost absorption, purchase).	○	○	○	○	○	○	○	○	○
6.1	Determine / formulate individual health values and realistic goals in a structured manner in dialogue with the residents.	○	○	○	○	○	○	○	○	○
6.2	Identify and plan nursing interventions to support individual health behaviour and support their implementation, for example through instruction, training, education, information and advice.	○	○	○	○	○	○	○	○	○
6.3	Apply nursing strategies to change health conditions, for example by promoting supportive social networks or health promotion measures related to the setting of the NH and for all people living and working there.	○	○	○	○	○	○	○	○	○
6.4	Lead multi-professional case reviews, in which the development processes of undesired or adverse events are reflected upon and preventive measures are initiated.	○	○	○	○	○	○	○	○	○

#		1	2	3	4	5	6	7	8	9
6.5	Derive recommendations and measures from the nursing visits beyond the nursing care service (risk factor-related measures of primary, secondary and tertiary prevention: e.g. prophylaxis).	◯	◯	◯	◯	◯	◯	◯	◯	◯

What other comments would you like to add to this competency area?

Are there any activities missing that you think could be of high importance?

Please enter your answer here:

Empowerment and communication with residents

This competency area includes the fields of action of communication with (relatives and) residents, advocacy, prioritization of care problems with resident preferences, psychosocial integration, and advance care planning.

Note on the meaning of the scale:

Levels 1 to 3 correspond to low relevance / priority

Levels 4 to 6 mean that these tasks are important but not of crucial importance.

Levels 7 to 9 correspond to the highest priority and crucial importance.

Please select the answer for each point:

#		1	2	3	4	5	6	7	8	9
1.1	Transparently present responsibilities in NH area:<br>1. Legible nameplates, overview of staff at a wall, laminated overviews with photos and names of staff per residential unit in the resident's room,<br>2. Information letter: Overview of the "most important" contact person for relatives, including responsibilities.	○	○	○	○	○	○	○	○	○
2.1	Informe the residents about nursing assessments as well as the benefits and risks of interventions in order to enable them to make an informed decision (= informed consent).	○	○	○	○	○	○	○	○	○
2.2	Consider person-centered communication in nursing visits or case discussions: Encourage residents to communicate. Design communication as a trialogue (equal cooperation between residents, relatives and professionals).	○	○	○	○	○	○	○	○	○
3.1	Regular nursing visits and case discussions involving residents, relatives or persons of trust. Clarify conflicting assessments of care problems with resident preferences.	○	○	○	○	○	○	○	○	○
4.1	Structured observation of psychosocial well-being after moving in. Individual support for integration into the NH and everyday life.	○	○	○	○	○	○	○	○	○

#		1	2	3	4	5	6	7	8	9
5.1	Offer structured advice on advance care planning for residents and their relatives and document the results of the advice (contents, decisions, powers of attorney).	○	○	○	○	○	○	○	○	○
5.2	Ensure that the presumed or documented will to initiate life-prolonging measures is known in the medical and nursing team and promote its consideration in decisions.	○	○	○	○	○	○	○	○	○

What other comments would you like to add to this competency area?

Are there any activities missing that you think could be of high importance?

Please enter your answer here:

Organization / Facility

This area of competence includes the fields of action of care quality based on current scientific standards, care level management, dealing with rules and standards, internal distribution of tasks in the care process: organizing skill mix, designing learning processes, internal communication / documentation, values and norms of the facility, and social network as a resource.

Note on the meaning of the scale:

Levels 1 to 3 correspond to low relevance / priority

Levels 4 to 6 mean that these tasks are important but not of crucial importance.

Levels 7 to 9 correspond to the highest priority and crucial importance.

Please select the answer for each point:

#		1	2	3	4	5	6	7	8	9
1.1	Reflect on nursing practice, identify problem areas and formulate research questions. Search for evidence, evaluate it, prepare it for practice and communicate it within the NH	○	○	○	○	○	○	○	○	○
1.2	Work with expert standards and nursing guidelines:<br>1. Adapt and prepare to the context of the NH<br>2. Evaluate application	○	○	○	○	○	○	○	○	○
1.3	Establish training and learning opportunities on evidence-based care, expert standards and nursing guidelines in NH	○	○	○	○	○	○	○	○	○
1.4	Communicate educational and networking offers from professional nurse associations in the NH.	○	○	○	○	○	○	○	○	○
2.1	Determine changes in the care needs of residents and initiate the need for a review of the degree of provided and refinanced care (if necessary, cooperation with relatives or carers).	○	○	○	○	○	○	○	○	○

#		1	2	3	4	5	6	7	8	9
3.1	Regular evaluation of the use of standards in the NH. Regular adjustment of standards based on current evidence.	○	○	○	○	○	○	○	○	○
3.2	Development of decision-making questions/aids for common phenomena that result in hospital admission (fall, dehydration, personality change).	○	○	○	○	○	○	○	○	○
4.1	Description of tasks, responsibilities and information distribution:<br>• within job descriptions<br>• through the appointment of the persons responsible for the support in the respective process.<br>Application and implementation:<br>• concepts for training of new nursing staff<br>• structured reflection and communication about teamwork with the team	○	○	○	○	○	○	○	○	○
5.1	Management of the reflection on decision-making with the nursing team within:<br>• Structured case reviews<br>• Collegial advice<br>• Staff supervision	○	○	○	○	○	○	○	○	○
6.1	Structure handover of care to ensure priority information transfer (e.g. "SBAR" concept)	○	○	○	○	○	○	○	○	○
7.1	Reflection on values that guide nursing and development of a "good care" model together with the NH team.	○	○	○	○	○	○	○	○	○
8.1	Working with social resources: Analysis of the social network, communication with the social network, maintaining and documenting contacts.	○	○	○	○	○	○	○	○	○

What other comments would you like to add to this competency area? Are there any activities missing that you think could be of high importance?

Please enter your answer here:

Submit your survey.

Thank you for completing the questionnaire.

Chapter C: Algorithm for decisions on intervention components

The algorithm defines key events and a pathway to include intervention components according to the respective event. Figure C6 shows the key events during residency at the nursing home.

Figure C7 shows the algorithm for the key event "Health deterioration".

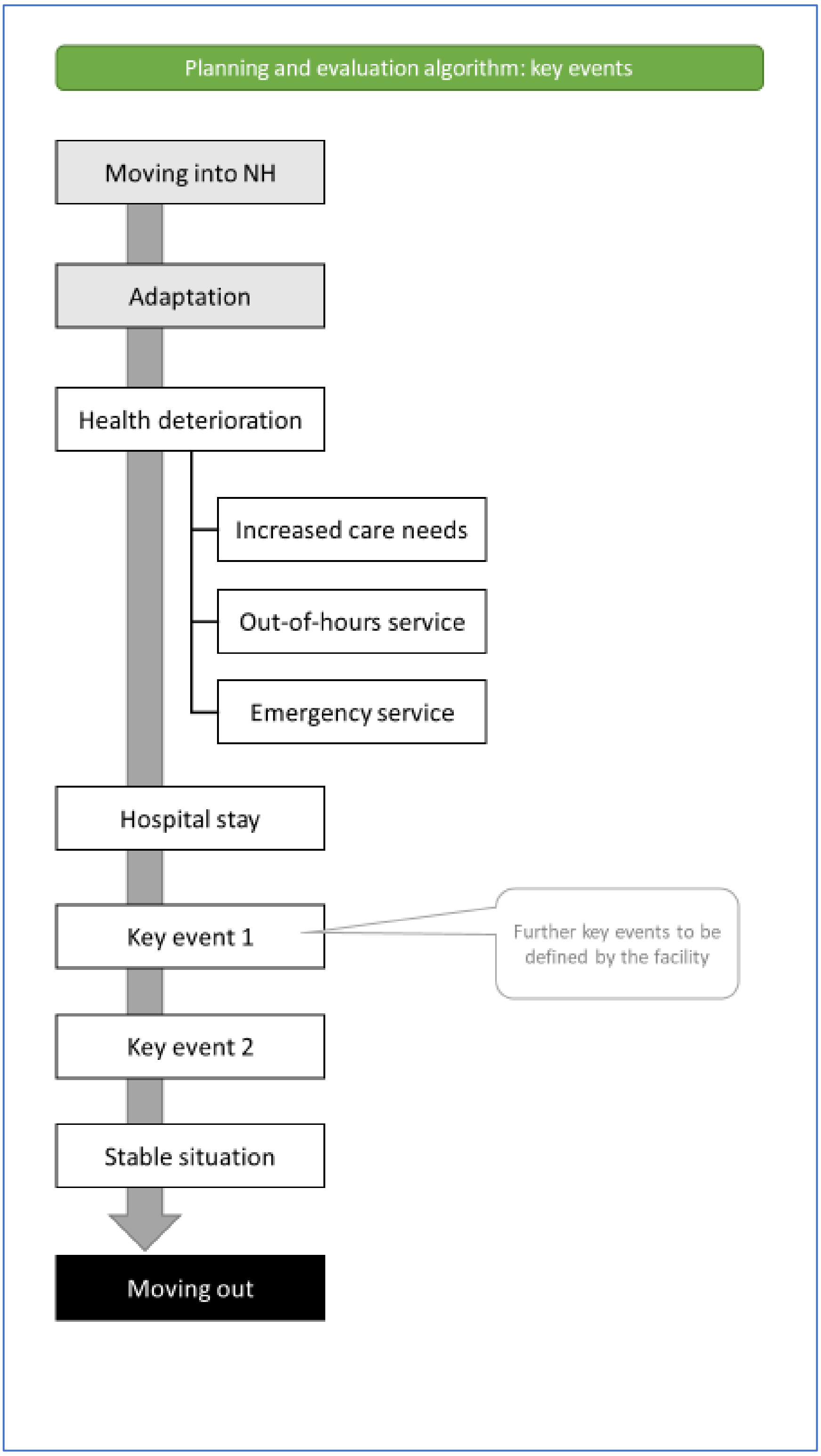

Figure C6 Key events for care planning and evaluation

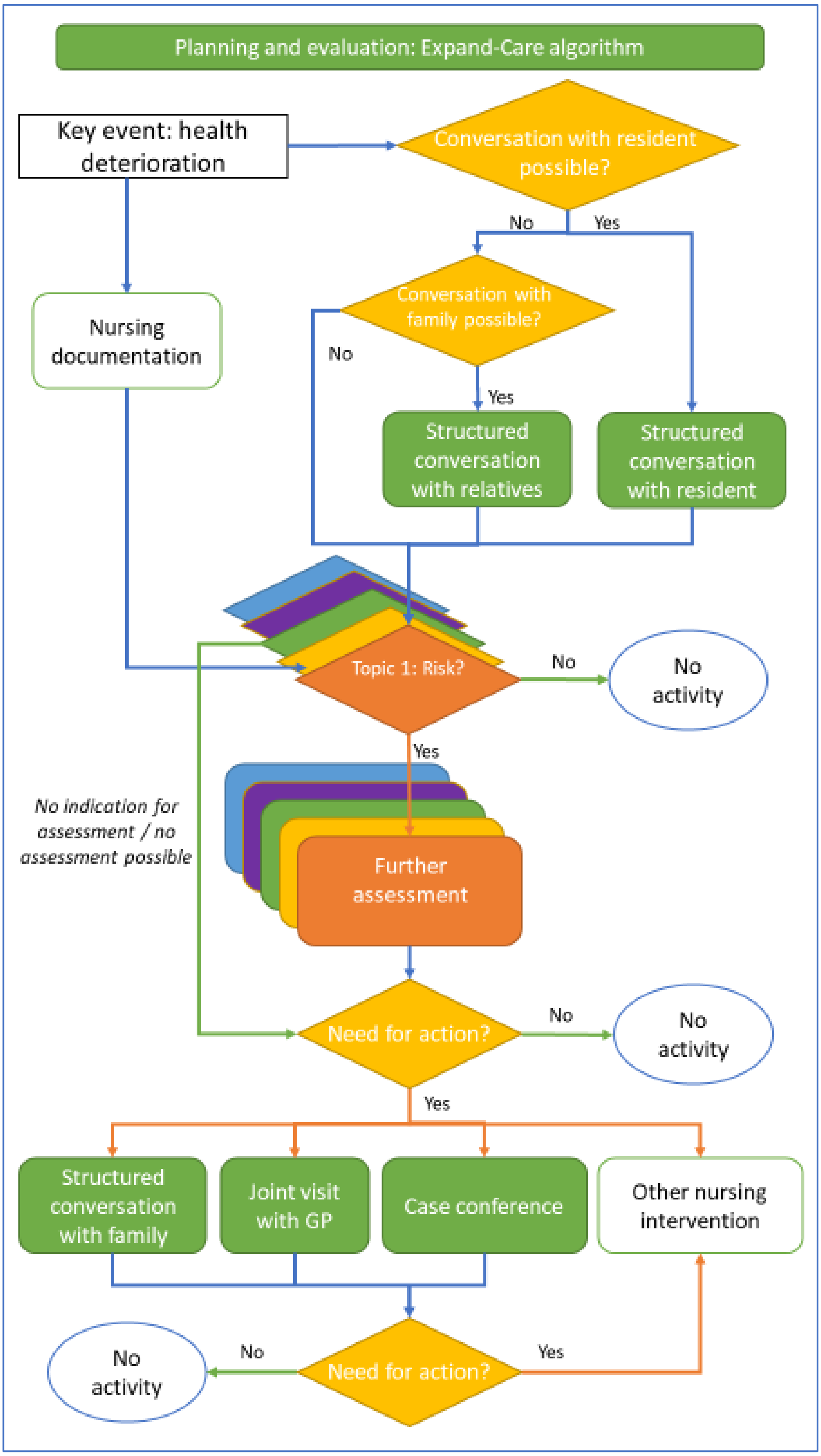

Figure C7 Planning and evaluation algorithm
