# Supplementary material for: A new role profile for nurses with expanded competencies promoting person-centered care in long-term care: a mixed-methods intervention development study
Source: BMC Geriatr. 2025 Jul 5;25:492. doi: 10.1186/s12877-025-06086-2 (PMC12228253; doi:10.1186/s12877-025-06086-2)
Supplement: Supplementary file 2 — Supplementary Material 2. [file 12877_2025_6086_MOESM2_ESM.pdf]

Supplement 2 to

A new role profile for nurses with expanded competencies promoting person-centered care in long-term care: a mixed-methods intervention development study.

Silies K, Huckle T, Pohontsch N, Jarchow AM, Schütz K, Müller M, Lühmann D, Balzer K

Curriculum

Nurse specialist with expanded competencies for person-centered care in long-term care (PEPA)

as part of the Expand-Care project:

Expanded nursing competencies to improve person-centered care for nursing home residents with complex health needs

May 2022

Contents

1. Profile and objectives of the qualification program 3

2. Fields of activity, skills and learning requirements 3

3. Classification of the qualification program in higher education in nursing 4

4. Learning objectives, content and modular structure 5

5. Learning and teaching formats and forms of examination 6

6. Teaching and organization (contact person) 7

7. Evaluation of the qualification program 7

8. Appendix 1: Participation requirements for nursing staff I

9. Appendix 2: Module Handbook PEPA Curriculum (10 CP) III

1. Profile and objectives of the qualification program

The curriculum for the additional qualification as a *nurse specialist with expanded competencies for person-centered care in long-term care (PEPA)* was developed as part of the Expand-Care project. [1]

The aim of the qualification program is to enable nursing staff with a bachelor's degree or comparable qualification to take on a new nursing role in long-term inpatient care facilities in the care of older people with complex health care needs. Resident-related tasks, particularly in the competence areas "Managing chronic and geriatric illnesses" and "Empowerment and communication " are intended to help shape health care in a person-centered way.

The PEPA role and its tasks are being piloted by the research group at the University Medical Center Hamburg-Eppendorf and the University of Lübeck.

If the pilot is successful, the program will be established as an extra-occupational qualification for nursing staff, thus ensuring the long-term transfer of scientific knowledge and higher education skills into nursing practice in long-term care facilities. At the same time, strategic cooperation between care facilities and universities is to be promoted.

2. Fields of activity, skills and learning requirements

The PEPA model includes components in the following areas of activity:

1. Planning, management, implementation and evaluation of care and multi-professional care processes based on the principles of person-centered care,
2. Evidence-based practice development and
3. Promotion of interprofessional collaboration.

The skills and learning requirements of the participants are considered through a participatory approach to selection by the cooperation partners of the long-term care facilities and the universities as providers of the educational program. It is assumed that, ideally, a nursing qualification at German Qualifications Framework (DQR) level 6 (Bachelor's degree or equivalent) is required for the professionally appropriate implementation of these tasks. This qualification level can be proven in various ways (Table 1, one criterion must be fulfilled).

A further requirement is employment in a long-term care facility with at least 80% of a full-time position.

Participants take part in an initial interview with representatives of the universities in order to be able to address individual strengths and learning needs as part of the qualification program.

[1] *Expand-Care (Expanded nursing competencies to improve person-centered care for nursing home residents with complex health needs) funded by the Federal Ministry of Education and Research (FKZ 01GY2003A and 01GY2003B)*

Table 1: Inclusion criteria for nurses to the Expand Care program (one criterion must be met)

	Criterion	Comment
1.	Bachelor's degree in nursing with at least one year of professional experience after the vocational graduation.	In dual study programs, professional experience can be credited after completion of the vocational graduation even before obtaining the Bachelor's degree.
2.	Three-year vocational training with additional specialist training in palliative care or geriatric psychiatry	
3.	Three-year vocational training with above-average further training activities totaling 300 hours in 2 years.	This corresponds to the scope required by the registration office for professional carers for registration. All (internal and external) training courses for which participation is documented can be credited. The training courses do not have to be recognized by the registration office for professional nurses.
4.	Three-year vocational training with above-average skills in the following areas<br>• Knowledge and skills<br>• Openness to innovation and further development of professional practice<br>• Personal skills	The generic descriptors for informal learning and the criteria of the DQR in the area of personal competencies serve as a guide (see appendix). Competencies are assessed by a superior who knows the employee concerned sufficiently.

3. Classification of the qualification program in higher education in nursing

The University of Lübeck currently offers two educational programs related to nursing. The dual Bachelor's degree program leads to a professional qualification in nursing and a Bachelor of Science within eight semesters. The Master's degree program in Health and Care Sciences leads to a Master of Science within four semesters. A part-time course leading to a Bachelor of Science degree for employed nurses is in preparation.

The qualification program presented here offers practicing nurses the opportunity to deepen their knowledge at a university education level and transfer it into practice. Thanks to modularization, the qualification program is compatible with existing university programs in the field of nursing and the credits earned can be partially credited when taking up a Bachelor's degree course, depending on the field of study.

The University of Lübeck strives to link research and practice in health science. The qualification program contributes to this goal by specifically targeting practitioners and contributing to evidence-based nursing care without having to take up a full study program. It is explicitly intended both as support for evidence-based practice and as an introduction to nursing science.

With this program, the University of Lübeck is responding to a need to support the professional development of professionals that long-term care facilities who are research and practice partners of the University have reported back. The qualification program, promotes the partnership of the university and practice facilities. Prerequisite for participation is therefore employment by a partner institution and support for participants in implementing the on-the-job training elements of the qualification program.

Individualized training and coaching of the PEPA by an experienced nurse with a qualification at DQR level 7 (Master's degree) or higher ensures that learners can apply their skills in professional practice.

4. Learning objectives, content and modular structure

Learning objectives of the qualification program are described in relation to the levels of knowledge and understanding, use, application and generation of knowledge, self-image / professionalization and communication and cooperation (module handbook, Annex 2).

Knowledge and understanding

Learning objectives in this area include, for example, classifying and describing the role as a PEPA, methods of evidence-based practice, (interprofessional) communication and collegial collaboration as well as supporting evidence-based patient information and decision-making. When dealing with chronic diseases, it includes, for example, explaining geriatric syndromes and selected chronic diseases as well as symptoms of exacerbation of these conditions.

Use, application and generation of knowledge

Learning objectives in this area include the self-organized application of learning strategies, the identification of communication structures within participants' own institution and reflection on sources of error. In Module 2, the learning objectives relate to the selection and application of clinical and geriatric assessment tools, the identification of acute symptoms and the derivation of action requirements. It also included the design of case reflections within the facility's internal team.

Self-image / professionalization

Part of this learning objective category is the goal of evaluating and further developing the role and limits of PEPA and being able to represent and classify one's own professional role both within the institution and in the multi-professional team.

Communication and cooperation

The learning objectives in this area are the resource- and person-centered design of conversations with those in need of care, relatives, nursing staff and members of other professions as well as the provision of advice in the sense of shared decision making. When dealing with chronic illnesses, the learning objectives include communicating the results of geriatric and clinical assessments in a target group-specific, professionally correct and precise manner.

The following teaching content is provided to achieve the learning objectives:

- General introduction to theories and models of extended practice, including aspects of role identification, implementation and cooperation with nursing colleagues in the facilities
- Care and target group-relevant topics (e.g. hospital admissions, nutritional status, wound development, chronic pain, mobility/falls, quality of life)
- The Expand Care intervention model

The course content is taught in two modules:

- Module 1: Expanded roles and competencies for care professionals
  - Introduction to the role of the care professional with expanded competencies in person-centered geriatric care
  - Communication and coaching
- Module 2: Person-centered care and support for people with chronic illnesses

- Diagnosis and treatment of chronic and geriatric diseases
- Person-centered care and support

Each module describes the topics/sub-topics, learning objectives, learning content, methods, workload and the form in which the results are to be secured or the performance certificates issued (module handbook, Annex 2).

The workload, which forms the basis for the allocation of European Credit Transfer and Accumulation System (ECTS) credits to the individual modules or courses, is expressed in hours. One credit point corresponds to an average workload of 30 hours. The workload is made up of the time that students have to spend preparing and following up the course material (classroom teaching and self-study), preparing and completing coursework, and on-the-job training in order to achieve the specified learning outcomes.

Five credit points can be earned in each module; the maximum possible number of 10 credit points leads to successful completion of the qualification program. A corresponding certificate of successful participation is the basis for recognition of the achievements as ECTS in a subsequent university degree course.

5. Learning and teaching formats and forms of examination

Four learning and teaching formats are defined as part of the qualification program:

1. **Contact hours / attendance**
   Contact hours include courses in which lecturers from the university or external lecturers are present and lead or accompany the course. These include
   - Lecture / Presentation
   - Supervision
   - Individual counseling or support sessions

2. **Self-study**
   Self-study is defined as learning formats that are organized and carried out independently by the learners. These include
   - Independent development or repetition of learning content (own research or with materials provided by the university)
   - Creating term papers
   - Working in learning groups
   - Preparation for performance assessments

3. **Training on the job**
   On-the-job training is defined as learning formats in which learners apply methods in their everyday practice and which are recorded, supervised or observed. This can include, for example
   - Case conferences
   - Team activities
   - Establishing new processes (e.g. restructured nursing handover or ward rounds)

6. Teaching and organization (contact person)

Teaching is organized by the project management, the academic staff and research assistants in the Expand-Care project. The organization includes the provision of curricula for participants, the recruitment of lecturers, the conduct of performance assessments and the coordination of various learning formats.

Responsible for the content of the teaching is: [Anonymized]

Responsible for the organization of the qualification offer and contact person for the participants and lecturers is: [Anonymized] (Research Assistant Expand-Care HL)

7. Evaluation of the qualification program

The evaluation of the qualification program will be part of the process evaluation in the Expand Care pilot study.

8. Appendix 1: Participation requirements for nursing staff

The Expand-Care project aims to develop and test new roles for academically qualified nursing staff in long-term inpatient care. In a participatory process, an intervention was developed to promote person-centered care for older people with complex care needs. This intervention is to be implemented by care professionals with a qualification level comparable to DQR 6, which corresponds to a Bachelor's degree. However, a comparable level of qualification can also be achieved through training with additional criteria. In addition, from the perspective of the research group and stakeholders involved in the research process, further personal skills are relevant prerequisites for the successful implementation of the intervention. For this reason, differentiated access options (participation requirements, inclusion criteria) were defined for care professionals in the Expand Care project at three levels.[2]

1. Knowledge and skills
2. Openness to innovation and further development of professional practice
3. Personal skills

On the one hand, this takes into account the actual low availability of bachelor-qualified nurses. On the other hand, the existing skills of nurses are also to be recognized and particularly qualified practitioners are to be given access to higher education.

Access options for care professionals to the Expand Care program

	Criterion	Comment
1.	Bachelor's degree in nursing with at least one year of professional experience after graduation.	In dual study programs, professional experience can be credited after completion of the vocational degree even before obtaining the Bachelor's degree.
	OR	
2.	Three-year vocational training with additional specialist training in palliative care or geriatric psychiatry	
	OR	
3.	Three-year vocational training with above-average further training activities totaling 300 hours in 2 years.	This corresponds to the scope required for registration by the registration office for professional carers.<br>All (internal and external) training courses for which participation is documented can be credited. The training courses do not have to be recognized by the Registration Office for Professional Nurses.
	OR	
4.	Three-year vocational training with above-average skills in the following areas<br>• Knowledge and skills<br>• Openness to innovation and further development of professional practice<br>• Personal skills	The generic descriptors for informal learning and the criteria of the DQR in the area of personal competencies serve as a guide (see appendix). Competencies are assessed by a superior who knows the employee concerned sufficiently.

[2] https://www.dqr.de/dqr/de/der-dqr/der-dqr_node.html, on 29.03.2022
https://uol.de/fileadmin/user_upload/anrechnungsprojekte/Download/Artikel_Generische_Deskriptoren_GDIL.pdf, on 29.03.2022

Generic descriptors for informal learning (based on the joint project "Development of part-time study programs in nursing and health sciences")

The following statements refer to an area of activity in which the nurse has acquired extensive skills without having participated in a formal training course (beyond basic training).

	Assessment: the majority of the criteria per area (A-C) should apply.	Applies	Does not apply
A)	***Knowledge and skills***		
1.	The level of knowledge of the nurse goes beyond the basic general knowledge of adults.		
2.	The nurse is familiar with the most important facts, principles, procedures and general terms that are relevant in the above-mentioned field of activity.		
3.	The knowledge of the nurse corresponds at least to the current level of text-book knowledge.		
4.	The nurse must be able to exchange information, ideas, problems and solutions with specialist representatives, for example with physicians.		
5.	The job requires the nurse to make extensive use of cognitive or practical skills.		
B)	***Openness to innovation and further development of professional practice***		
6.	The nurse is interested in the latest specialist knowledge and keeps up to date with it (e.g. in specialist journals).		
7.	The nurse shows willingness and active commitment to the implementation of expert standards and guidelines.		
8.	The nurse reflects critically on existing practice and is interested in adapting and developing routines in nursing practice.		
9.	The job confronts nurses with new challenges for which they develop their own solutions.		
10.	The area of activity includes dealing with ethical or social issues.		
11.	The nurse has experience in project work.		
C)	***Personal skills***		
12.	The nurse is characterized by above-average motivation and interest in profes-sional and personal development.		
13.	The nurse works responsibly in a team or takes responsibility for leading groups.		
14.	The nurse can guide the professional development of others and can deal with problems in the team proactively.		
15.	The nurse can argue complex problems and solutions to experts and develop them further with them.		
16.	The nurse can define, reflect on and evaluate objectives for learning and work processes and design learning and work processes independently and sustaina-bly.		

9. Appendix 2: Module handbook PEPA curriculum (10 CP)

<table>
<tr><th>Modules</th><th>Contents</th><th>Contact time (1 h = 1 teach-ing unit of 45 min)

Teaching formats</th><th>Training on the job</th><th>Self-study</th><th>CP
Total work-load</th><th>Academic achievements</th><th>Final module performance assessment</th></tr>
<tr><td>Module 1: Expanded roles and competencies for care professionals</td><td></td><td></td><td></td><td></td><td>5 CP
150 h</td><td></td><td>Presentation</td></tr>
<tr><td>Learning objectives Module 1</td><td colspan="7">Knowledge and understanding:
▪ Learners can describe the objectives and process of the Expand Care project as well as the core elements and objectives of the Expand Care curriculum
▪ They can categorize the role of the PEPA based on a role definition and the existing structures in care facilities as well as social expectations
▪ They can describe important legal framework conditions for nursing work
▪ They can describe the steps of the EBN method and various methods of gaining scientific knowledge
▪ They can describe the structure of scientific work and name access routes to care-related publications
▪ They can explain the communication model according to Watzlawick and Rogers as well as aspects of Rosenberg’s non-violence communica-tion theory
▪ They can name the potential uses of communication tools in healthcare and feedback methods
▪ They can state basic aspects of interprofessional communication
▪ They can describe the basics of didactic theories and methods of peer counseling and guidance
▪ They can describe different sources of errors in nursing care and strategies for dealing with errors in organizations

Use, application and generation of knowledge:
▪ They can apply self-organized learning strategies alone or in small groups
▪ They can align their daily professional activities with their knowledge and understanding of the scientific principles
▪ They can formulate a suitable question for a targeted search of scientific literature based on specific problems in their own professional prac-tice
▪ They can identify strategies and introduce tools or guidelines together with the care teams of the facilities in order to precisely formulate and document intra- and interprofessional reporting
▪ They can identify barriers, facilitating factors and problems with communication structures in their institution
▪ In conversational situations, they can reflect on the specific circumstances of the individual interlocutors and themselves and organize their conversations in an individually appropriate way, taking into account the views of other participants
▪ They can identify and reflect on organization-specific and their own sources of error in nursing processes

Self-image / professionalization:
▪ They can evaluate their role as PEPA and develop it further</td></tr>
</table>

Modules	Contents	Contact time (1 h = 1 teaching unit of 45 min)<br><br>Teaching formats	Training on the job	Self-study	CP<br>Total workload	Academic achievements	Final module performance assessment
	▪ They can recognize and further develop their own tasks and special contribution to the care of people in need of care as well as boundaries and interfaces with other professions<br><br>**Communication and cooperation:**<br>▪ They can conduct targeted and resource-oriented discussions with people in need of care, their relatives, nursing staff and members of other professions<br>▪ They can formulate professional and relevant nursing measures within the scope of nursing activities and justify these in discourse with nursing professionals and members of other professions using theoretically sound arguments						
Introduction to the role of the care professional with expanded competencies in person-centered geriatric care	Introduction Expand-Care<br>Learning strategies<br>IE applications basics<br>Role model/role development<br>Legal and ethical principles<br>Evidence-based practice	**Total: 30 h**<br>Seminars<br>Lectures<br>Exercises (EDP, literature research)	30 h	30 h	3 CP<br>90 h	Exercises<br>Practice protocols / Journal Writing	
Communication and coaching	Communication<br>Coaching and consulting<br>Conflict management	**Total: 19.5 h**<br>Seminars<br>Lectures<br>Exercises (skills training with simulated patients)	20 h	20,5 h	2 CP<br>60 h	Proof of practice (skills training)<br><br>Reflection protocol	

<table>
<tr><th>Modules</th><th>Contents</th><th>Contact time (1 h = 1 teaching unit of 45 min)

Teaching formats</th><th>Training on the job</th><th>Self-study</th><th>CP
Total workload</th><th>Academic achievements</th><th>Final module performance assessment</th></tr>
<tr><td>Module 2: Person-centered care and support for people with chronic illnesses</td><td></td><td></td><td></td><td></td><td>5 CP
150 h</td><td></td><td>Case documentation and reflection (in written with discussion in the colloquium)</td></tr>
<tr><td>Learning objectives Module 2</td><td colspan="7">Knowledge and understanding:

Learners can explain the different components and objectives of geriatric assessments and clinical assessments
They can explain the development and care-relevant characteristics of geriatric syndromes
They can explain the etiology, pathogenesis, main symptoms, secondary diseases and symptoms of exacerbation of selected chronic diseases
They can explain common errors in drug application and strategies for avoiding errors
They can describe intended and undesired effects, interactions and suitable or unsuitable forms of administration of frequently used drugs in the treatment of chronic diseases with high relevance in the care of people with geriatric diseases
They can describe basic concepts of health promotion and prevention as well as models of person-centered care
They can explain ethical principles in nursing and medicine as well as models of decision-making
They can reflect on challenging situations in day-to-day care and describe ethical dilemmas, particularly in connection with the principle of autonomy, and name strategies for dealing with them
They can describe the concepts of empowerment and participation
They can explain concepts of ACP and health care planning and classify residents' wishes for end-of-life care according to the situation
They can explain aspects, goals and barriers to self-care in the nursing care of older people and classify them in the care process
They can describe the theoretical foundations and principles of shared decision making and evidence-based patient information

Use, application and generation of knowledge:

They can select, apply and evaluate geriatric and clinical assessment instruments according to indication and use the results to develop nursing measures as part of the nursing process
They can identify geriatric requirements and burdens related to residents and integrate these into the care process
They can classify the progression of a chronic illness in a linear fashion in relation to residents and proactively identify key areas of care
They can identify acute symptoms and derive the need for action
They can formulate individual health goals with residents
They can plan nursing care on the basis of a person-centered understanding of nursing care
They can organize ethical case reflections in the team and assign the consideration of the individual perspectives of those involved in everyday nursing care
</td></tr>
</table>

<table>
<tr><td></td><td colspan="7">

- They can establish standards for the documentation and implementation of care planning in their own facilities and develop solutions that promote compliance with the wishes of older people in the provision of care

Communication and cooperation:

- They can present and justify the results of geriatric assessments in communication with members of the multi-professional team in a target group-specific, professionally correct and precise manner
- They can design communication in a target group-oriented manner and in interprofessional dialog and with the involvement of residents and relatives
- They can reflect and take into account the individual perspectives of those involved in decision-making in their communication
- They can organize discussions with professionals and with residents and relatives about ACP and end-of-life care

Self-image / professionalism:

- They can assign their nursing role and area of responsibility in the care of people with acute symptoms and in pharmacological care
- They can represent and further develop their professional role with extended areas of responsibility in the person-centered care of people in need of care with chronic illnesses within the organizational structure of the care facility and the multi-professional care team
- They can critically reflect on the role of professionals in decision-making and represent an understanding of their role as advocates for residents
- They can categorize their professional role as being supportive of people's decision-making and responsible for implementing their wishes

</td></tr>
<tr><td>Diagnosis and treatment of chronic and geriatric diseases</td><td>Medical basics of common chronic diseases and geriatric syndromes, geriatric assessment, pharmacological and non-pharmacological therapy (focus on potentially inappropriate medication, common side effects), secondary prevention and early detection of acute deterioration, models of self-care and self-care promotion in chronic diseases (influencing factors, course of disease management, etc.)</td><td>Total: 35 h
Seminars
Lectures
Exercises (skills training with simulated patients)
Supervision (practical support)</td><td>20 h</td><td>20 h</td><td>2.5 CP
75 h</td><td>Online test certificates
OSCE</td><td></td></tr>
<tr><td>Person-centered care and support</td><td>Models, methods and instruments of person-centered nursing and care, case management, shared decision making, advance care planning, joint</td><td>Total: 23.5 h
Seminars
Lectures
Exercises (skills training with simulated patients)</td><td>25 h</td><td>30 h</td><td>2.5 CP
75 h</td><td>Minutes of case conferences or similar.</td><td></td></tr>
</table>

	goal-setting discussions (incl. involvement of relatives)	Supervision (practical sup-port)					
